# Palladium-Catalyzed Selective Amino- and Alkoxycarbonylation of Iodoarenes with Aliphatic Aminoalcohols as Heterobifunctional O,N-Nucleophiles

**DOI:** 10.1021/acs.joc.2c02712

**Published:** 2023-04-13

**Authors:** László Kollár, Attila Takács, Csilla Molnár, Andrew Kovács, László T. Mika, Péter Pongrácz

**Affiliations:** † ELKH-PTE Research Group for Selective Chemical Syntheses, Ifjúság u. 6, Pécs H-7624, Hungary; ‡ János Szentágothai Research Centre, University of Pécs, Ifjúság u. 20, Pécs H-7624, Hungary; § Department of General and Inorganic Chemistry, University of Pécs, Ifjúság u. 6, Pécs H-7624, Hungary; ∥ Department of Chemical and Environmental Process Engineering, Faculty of Chemical Technology and Biotechnology, Budapest University of Technology and Economics, Müegyetem rkp. 3, Budapest H-1111, Hungary

## Abstract

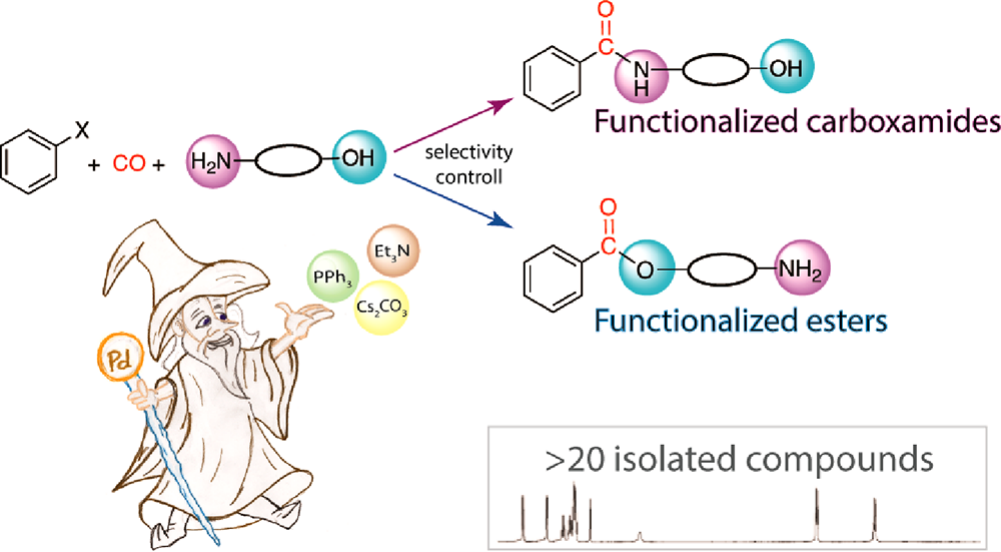

Palladium-catalyzed amino- and alkoxycarbonylation reactions of aryl iodides were investigated in the presence of aliphatic heterobifunctional N,O-nucleophiles. Selective synthesis of amide alcohols and amide esters was realized, controlled by the base and substrate ratio. The effect of iodobenzene substituents was also studied with surprising results in terms of product selectivity. In addition to the model ethanolamine/iodobenzene system, various heteroaromatic substrates and numerous related nucleophiles were tested under optimized conditions, providing moderate to good yields of the target compounds. Reactions of serinol and 1,3-diamino-2-propanol as model trifunctional compounds showed particularly high chemoselectivity on amide ester products. Considering the coordinative properties of the applied nucleophiles, a rational catalytic cycle was proposed.

## Introduction

Since the discovery of hydroformylation^1^ and the Reppe reaction,^2^ the transition metal-catalyzed carbonylation reactions, providing facile and even atom-economic methods for the incorporation of the C=O functionality into various skeletons, have become tremendously important in numerous fields of synthetic organic chemistry from laboratories to industrial applications. While several functional groups, i.e., C=C, C≡C, C–OH, etc., can be subjected to carbonylation,^3^ the transformation of aryl–X compounds (X = I, Cl, or Br) and cheap and readily available CO in the presence of a nucleophile(s) represents a fundamental catalytic procedure for manufacturing various amides, carboxamides, carboxylic acids, esters, heterocycles, azides, etc.^4^ However, it should be noted that several successful attempts were made to utilize an alternative CO source such as formaldehyde/paraformaldehyde,^5^ formic acid,^6^ etc.,^7^ due to the high toxicity of CO.

**Scheme 1:**
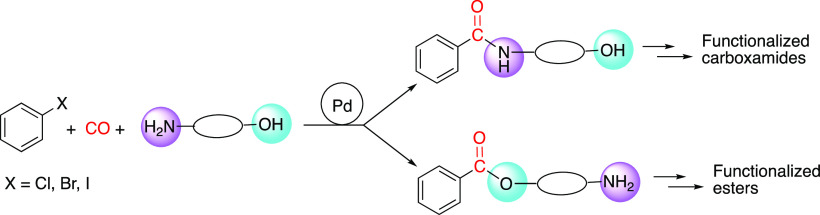
Synthesis of Functionalized Carboxamides and Esters via Carbonylation of Aryl–X Substrates

Among the broad scope of nucleophiles such as alcohols and phenols,^5a,8^ amines,^9^ thiols,^10^ or even water^11^ and selenols,^12^ O- and N-nucleophiles have been the most extensively studied for the manufacturing of the corresponding esters and amides that are significantly important in fine chemical and pharmaceutical industries.^13^ While several studies have been reported for the utilization of simple amines and alcohols as model substrates, only a few works focused on the investigation of carbonylation reactions in the presence of homobifunctional and even heterobifunctional nucleophiles. However, the applications of the latters could open alternative and easier synthetic routes for the construction of complex molecular structures (Scheme 1).

Alper and co-workers examined in detail the reaction of *p*- and *m*-aminophenols as nucleophiles in the carbonylation of unsaturated C,C bonds^14^ and iodoarenes.^15^ In the ligand- and additive-controlled aminocarbonylations of styrene^14a^ and alkynes,^14b^ high regioselectivity was achieved for linear and branched isomers equally. While aminophenols did not act as O-nucleophiles under these conditions, in the presence of iodoarenes, both amino- and phenoxycarbonylation could proceed with good selectivity toward amides and esters, respectively. It should be noted that only small amounts of amide ester derivatives were formed under all conditions. A different product distribution was detected for the reactions of *o*-aminophenols. It was shown that the aminocarbonylation step was followed by dehydrative cyclization, providing benzoxazoles in excellent yields.^16^

Similarly, benzimidazoles and benzthiazoles were formed by the reaction of *o*-phenylenediamine^17^ and *o*-aminothiophenol.^18^ Various diamines were introduced as N-nucleophiles into the carbonylation of iodoalkenyl and iodoaryl compounds by Pereira and co-workers.^19^ Good isolated yields of dicarboxamides were obtained with aromatic and aliphatic diamines, as well. Recently, we investigated the carbonylation of iodoalkenes in the presence of heterobifunctional ethanolamines as O,N-nucleophiles.^20^ Carboxamides were isolated as major products, but the corresponding amide ester derivatives were also detected in the reaction mixture.

On the basis of the interesting results with aliphatic heterobifunctional nucleophiles and due to sporadic preliminary studies in the literature, the evaluation of the carbonylation reactions of iodoarenes using various aminoethanols is highly desirable.

Herein, we report the investigation of the carbonylation reactions of iodoarenes using various aminoethanols to produce functionalized carboxamides and esters that could be promising intermediates in the production of complex molecular structures, especially in the pharmaceutical industry.

## Experimental Section

Palladium precursors, phosphine ligands, solvents, and other additives were purchased from Sigma-Aldrich Kft. (Budapest, Hungary) and used without further purification. The reactions were performed under an inert argon atmosphere using standard Schlenk techniques.

Conversion and selectivity were determined by a Shimadzu Nexis 2030 gas chromatograph, using splitless injection and a FID detector [column, DB-1MS (30 m × 0.250 mm × 0.25 μm); inlet temperature, 250 °C; starting oven temperature, 50 °C; rate, 15 °C/min; final temperature, 320 °C; carrier gas, He at 1.30 mL/min].

For routine MS measurements, a Shimadzu SPL-2010Plus gas chromatograph with a GCMS-QP2020 mass spectrometer with electrospray ionization (ESI) was used. The data are given for the corresponding compounds as mass unit per charge (*m*/*z*), and intensities are given in brackets in the Supporting Information.

HRMS experiments were performed on an orbitrap mass spectrometer. Exact mass measurements were performed on a high-resolution Q-Exactive Focus hybrid quadrupole-orbitrap mass spectrometer (Thermo Fisher Scientific, Bremen, Germany) equipped with a heated electrospray ionization source. Samples were dissolved in a 1:1 (v/v) acetonitrile/water solvent mixture containing 0.1% (v/v) formic acid. Flow injection analysis was performed using a 50 μL/min eluent flow. Under the applied conditions, the compounds form protonated molecules, [M + H]^+^, or sodium adduct ions, [M + Na]^+^, in positive ionization ESI.

The ^1^H and ^13^C NMR spectra were recorded on a Bruker Avance-III 500 spectrometer. Chemical shifts δ (parts per million) are given relative to solvent. References for CDCl_3_ were 7.26 ppm (^1^H NMR) and 77.16 ppm (^13^C NMR). Multiplets were assigned as s (singlet), d (doublet), t (triplet), q (quartet), dd (doublet of doublets), or m (multiplet).

In a typical experiment performed under atmospheric CO pressure, the catalyst precursor [Pd(OAc)_2_] (2 mol %, 2.25 mg) and ligand {4 mol % monophosphine [e.g., 5.24 mg of triphenylphoshine (TPP)] and 2 mol % diphosphine (e.g., 5.79 mg of XantPhos)} were placed in a three-neck flask and refilled with argon gas three times. DMF (10 mL), iodobenzene (0.5 mmol. 56 μL), ethanolamine (0.5 mmol, 30 μL), and Et_3_N (2.0 mmol, 278 μL) were transferred to the flask. The atmosphere was changed to carbon monoxide (1 bar). The mixture was heated (100 °C) and stirred with a magnetic stirrer for 24 h. After the reaction was completed, the mixture was cooled, filtered, and immediately analyzed by GC and GC-MS. GC yields were determined by using ethylparabene as the external standard.

In a typical high-pressure experiment, the catalyst precursor [Pd(OAc)_2_] (2 mol %, 2.25 mg) and TPP (4 mol %, 5.24 mg) were placed in a stainless steel autoclave and the atmosphere was refilled with argon gas three times. DMF (10 mL), iodobenzene (0.5 mmol, 56 μL), cysteamine (0.5 mmol, 38.6 mg), and Et_3_N (2.0 mmol, 278 μL) were transferred to the autoclave. The reaction vessel was pressurized to 60 bar of CO and heated with a heat-on block system (100 °C), and the mixture was stirred with a magnetic stirrer for 24 h. After the autoclave had been cooled and vented, the pale-yellow solution was removed and immediately analyzed by GC. GC yields were determined using ethylparabene as the external standard.

The details of the screening of the reaction conditions for carbonylation of iodobenzene and aminoethanol as well as details of the investigation of the *para*-substituent effect and competitive reactions are given in the Supporting Information.

The general procedure of product isolation and/or purification, the general procedure for the hydrolysis of amide esters to amide alcohols and carboxylic acids, and characterization and spectroscopic data (^1^H, ^13^C, and MS) of prepared compounds are given in the Supporting Information.

## Results and Discussion

2-Aminoethanol (**2a**) was selected as model heterobifunctional nucleophile for the optimization of the palladium-catalyzed carbonylation of iodobenzene (**1a**). The initial catalyst system was selected as the generally used Pd(OAc)_2_, 2 equiv of triphenylphosphine, triethylamine as a base, and DMF as a solvent under atmospheric carbon monoxide pressure (Table 1, entry 1). In addition to a nucleophile:substrate ratio of 1:1, the amide (**3a**) and amide ester (**4a**) products were formed in favor of the doubly carbonylated derivative. Single alkoxycarbonylation was not obtained; thus, ester amine derivatives cannot be detected in the reaction mixture. First, the effect of phosphine ligands was monitored keeping the Pd:P donor ratio of 1:2. Comparable activity and selectivity resulted from changing TPP to tricyclohexyl phosphine or DPEPhos (Table 1, entry 2 or 5, respectively), but much lower GC yields were obtained with most of the diphosphines (Table 1, entries 3, 4, 7, and 8). 1,2-Bis(di-*tert*-butylphosphinomethyl)benzene (Table 1, entry 6) was the only ligand, which reversed the selectivity by an acceptable product yield. The effects of bases and solvents were subsequently investigated using TPP as the ligand. Inorganic Na_2_CO_3_ gave results similar to those of Et_3_N, but almost complete amide selectivity was achieved in the presence of Cs_2_CO_3_ with a 67% yield of **3a** (Table 1, entry 10). Similar amide selectivity occurred with other phosphines using Cs_2_CO_3_ as the base (Table 1, entries 12–14). When 1,1,3,3-tetramethylguanidine was used, no carbonylation reaction occurred. Likewise, significant activity loss was observed with other solvents (Table 1, entries 15–17). Because no double CO insertion occurred under the applied conditions, a higher-pressure experiment was conducted (Table 1, entry 18). Under 60 bar of CO, only 11% ketocarboxamide and 2% ketocarboxamide ester derivative were detected by GC-MS. With an increase in the iodobenzene:aminoethanol ratio to 2:1, the level of esterification was increased as expected irrespective of the base or ligands (Table 1, entries 19–22), achieving a 90% yield of amide ester derivative **4a**. As a result of the optimization of the reactions, two conditions were selected: condition A for selective aminocarbonylation and condition B for amino-alkoxycarbonylation targeting the amide and ester amide products, respectively.

**Table 1:**
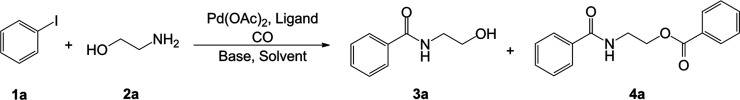
Optimization of the Reaction Conditions for Carbonylation of Iodobenzene and Aminoethanol^a^

					GC yield (%)^b^	
	ligand	base	solvent	**1a**	**3a**	**4a**
1	TPP	Et_3_N	DMF	1 equiv	11	62
2	Cy_3_P	Et_3_N	DMF		21	66
3	DPPB	Et_3_N	DMF		3	1
4	DCyPB	Et_3_N	DMF		7	0
5	DPEPhos	Et_3_N	DMF		18	52
6	D*t*BuPBenz	Et_3_N	DMF		42	16
7	Xantphos	Et_3_N	DMF		8	9
8	DPPF	Et_3_N	DMF		8	12
9	TPP	Na_2_CO_3_	DMF		18	55
10	TPP	Cs_2_CO_3_	DMF		67 (56)^c^	**2**
11	TPP	TMG	DMF		0	0
12	Cy_3_P	Cs_2_CO_3_	DMF		57	2
13	DPEPhos	Cs_2_CO_3_	DMF		60	1
14	D*t*BuPBenz	Cs_2_CO_3_	DMF		46	1
15	TPP	Et_3_N	MeCN^d^		10	0
16	TPP	Et_3_N	GVL		0	0
17	TPP	Et_3_N	NMP		8	1
18^e^	TPP	Et_3_N	DMF		57 (11)^f^	23 (2)^f^
						
19	TPP	Et_3_N	DMF	2 equiv	10	90 (69)^c^
20	TPP	Cs_2_CO_3_	DMF		15	85
21	Xantphos	Et_3_N	DMF		17	82
22	Xantphos	Cs_2_CO_3_	DMF		20	80

a Abbreviations: Cy_3_P, tricyclohexylphosphine; DPPB, 1,4-bis(diphenylphosphino)butane; DCyPB, 1,4-bis(dicyclohexylphosphino)butane; DPEPhos, bis[(2-diphenylphosphino)phenyl]ether; DtBuPBenz, 1,2-bis(di-*tert*-butylphosphinomethyl)benzene; DPPF, 1,1′-bis(diphenylphosphino)ferrocene; Xantphos, 4,5-bis(diphenylphosphino)-9,9-dimethylxanthene; GVL, γ-valerolactone; NMP, *N*-methyl-2-pyrrolidone. Reaction conditions: aminoethanol (0.2 mmol), iodobenzene (1 or 2 equiv), Pd(OAc)_2_ (2 mol %), 1:2 Pd/P, base (0.4 mmol), solvent (2 mL), *p*_CO_ = 1 bar, *T* = 100 °C, *t* = 24 h.

b GC yields were determined with an external standard (ethylparabene), calculated for iodobenzene.

c Isolated yields in parentheses.

d Reaction temperature of 82 °C.

e *p*_CO_ = 60 bar.

f GC yield of ketocarboxamide. Complete conversions were detected in all cases.

Additional carbonylation reactions were performed in the presence of aminoethanol with a set of substituted iodobenzenes in a ratio of 1:1 (Table 2). The results showed that substituent electronic properties have a significant effect on product selectivity that varied between 12% and 78%. The results with electron-donating groups (EDGs) provided results comparable to those for iodobenzene (Table 2, entries 1–3), but electron-withdrawing (EWG) chloro- and trifluoromethyl substituents significantly changed the product ratios (Table 2, entries 4 and 5, respectively). The increased amount of the “monocarbonylated” amides can be explained by the higher reaction rate of EWG-substituted derivatives in aminocarbonylation reaction; thus, the faster consumption of the substrate prevents the esterification step. The amide product especially prevails with 1-iodo-3,5-bis(trifluoromethyl)benzene (Table 2, entry 6).

**Table 2:**
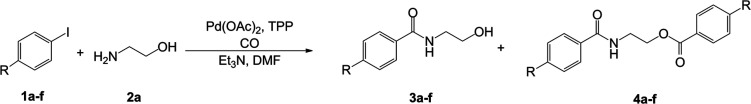
Effects of Substituents on the Carbonylation with Aminoethanol^a^

		GC yield (%)^b^		
	R	**3**	**4**	amide selectivity (%)
1	H (**1a**)	11 (**3a**)	62 (**4a**)	15
2	Me (**1b**)	12 (**3b**)	80 (**4b**)	13
3	OMe (**1c**)	10 (**3c**)	72 (**4c**)	12
4	Cl (**1d**)	20 (**3d**)	62 (**4d**)	24
5	CF_3_ (**1e**)	48 (**3e**)	46 (**4e**)	51
6	3,5-CF_3_ (**1f**)	82 (**3f**)	18 (**4f**)	78

a Reaction conditions: substrate (0.2 mmol), ethanolamine (**2a**, 0.2 mmol), Pd(OAc)_2_ (0.004 mmol), 1:2 Pd/TPP, Et_3_N (0.4 mmol), DMF (2 mL), *p*_CO_ = 1 bar, *T* = 100 °C, *t* = 24 h.

b GC yields were determined with an external standard (ethylparabene), calculated on iodobenzene. Complete conversions were detected in all cases.

Further competitive experiments were conducted in the presence of two different *para*-substituted iodobenzenes in equimolar amounts, while the substrate:aminoethanol ratio was kept at 2:1 (Table 3). As mentioned, the “dicarbonylated” amide ester products dominated all reactions. Only traces or small amounts of amides were formed after 24 h. Upon comparison of the substrate pairs, it can be stated that the amides substituted with EDGs were alkoxycarbonylated totally, while some EWG-containing amides remained in the reaction mixture. In other words, the chloro- and trifluoromethyl-substituted (substrates) amide derivatives tend to resist the alkoxycarbonylation step. The total ester–amide selectivities show that the more EWG derivatives participate dominantly in the aminocarbonylation step. For example, in the presence of H/OMe and Me/OMe substrate pairs, 2 times more benzamide derivatives were formed (Table 3, entries 1 and 3, respectively).

**Table 3:**
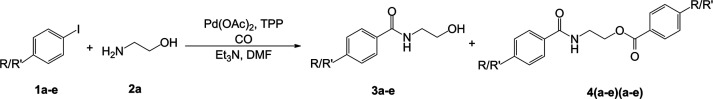

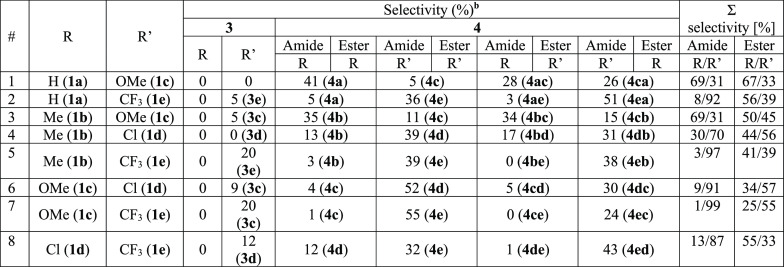
Competitive Carbonylation Reactions of Substituted Iodobenzenes^a^

a Reaction conditions (B): substrate (R + R′, 0.2 + 0.2 mmol), ethanolamine (**2a**, 0.2 mmol), Pd(OAc)_2_ (0.004 mmol), 1:2 Pd/TPP, Et_3_N (0.4 mmol), DMF (2 mL), *p*_CO_ = 1 bar, *T* = 100 °C, *t* = 24 h.

b Selectivities were determined by GC-MS. The selectivity of mixed (**4a–e**) products was determined after hydrolysis of the ester moiety. Complete conversions were detected in all cases.

Similarly, chlorobenzamide formation prevailed using Cl/Me- and Cl/OMe-substituted iodobenzenes (Table 3 entries 4 and 6, respectively). This effect is especially pronounced in the presence of 4-trifluoromethylbenzene, where 92–99% of products bear the CF_3_ derivative in the amide side (Table 3, entries 2, 5, and 7). Practically, no aminocarbonylation occurs with 4-iodoanisole in the presence of 4-trifluoromethyl iodobenzene (Table 3, entry 7). With regard to the esterification step, we can say that the product ratios are much more equalized. We can suppose that the substrates possessing more electron-withdrawing groups are more reactive in alkoxycarbonylation, as well. More benzoate and chlorobenzoate derivatives are formed using Me and OMe derivatives as substrate pairs (Table 3, entries 1 and 3 and entries 4 and 6, respectively). Interestingly, the ester selectivity is reversed in the presence of CF_3_, which can be explained by the fast consumption of this reagent in the aminocarbonylation step (entries 2, 5, and 8).

Our investigations were subsequently focused on the reactions of different heteroaromatic substrates and various N,O-nucleophiles (Tables 4 and 5). 3-Iodopyridine, 2-iodothiophene, and 1-iodoisoquinoline were involved in the aminocarbonylation reaction with aminoethanol as a heterobifunctional nucleophile to form the corresponding amides (**3g**–**i**, respectively). On the basis of the optimization of the reactions, Cs_2_CO_3_ was used to attain the amide alcohols under a 1:1 nucleophile:substrate ratio. In addition, using Et_3_N as the base and an increased amount of substrate led to good isolated yields of the ester amide products (**4g** and **4h**). The dicarbonylated derivative of isoquinoline (**4i**) cannot be detected in the reaction mixture.

**Table 4:**
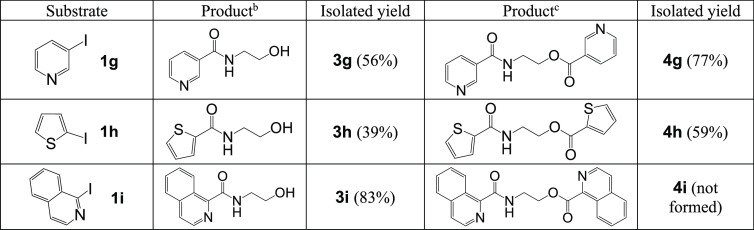
Carbonylation of Iodo Heteroaromatics in the Presence of Aminoethanol^a^

a Reaction conditions: substrate, ^b^0.5 mmol or ^c^1.0 mmol; ethanolamine (**2a**), 0.5 mmol; Pd(OAc)_2_, 0.01 mmol; 1:2 Pd/TPP; base, ^b^Et_3_N (2.0 mmol), ^c^Cs_2_CO_3_ (2.0 mmol); DMF (10 mL), *p*_CO_ = 1 bar, *T* = 100 °C, *t* = 24 h.

**Table 5:**
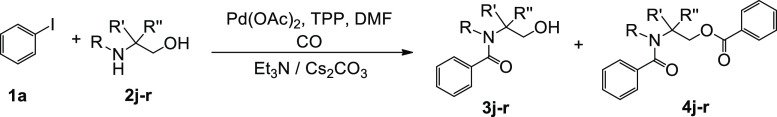

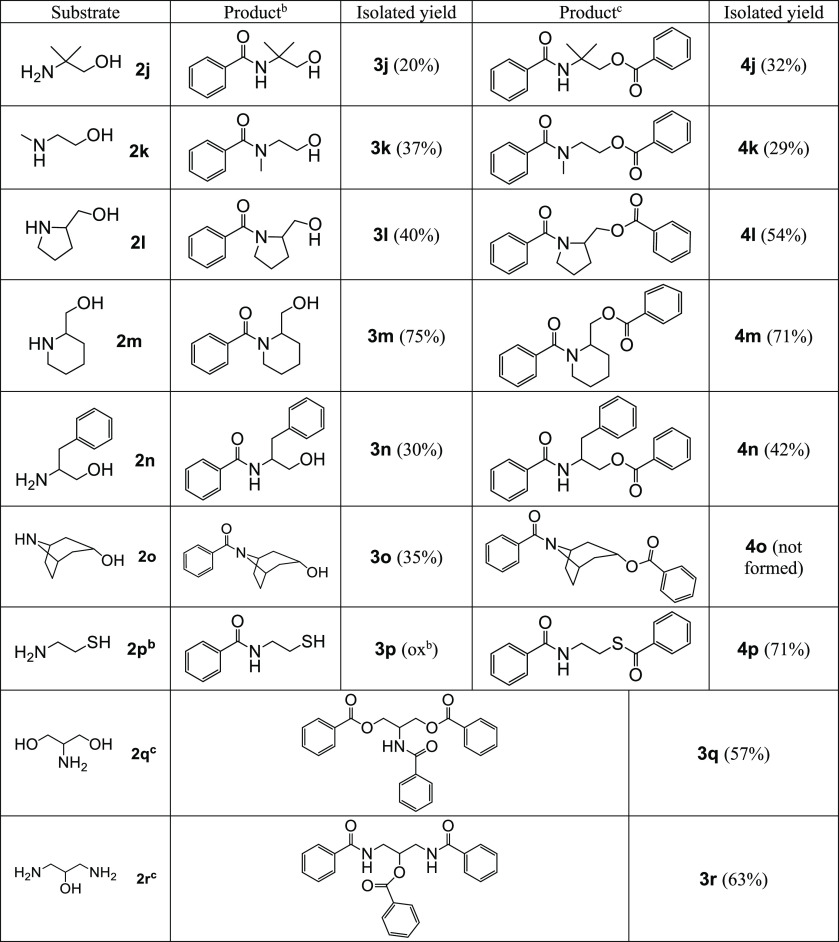
Carbonylation of Iodobenzene in the Presence of Heterobifunctional Nucleophiles^a^

a Reaction conditions for amide alcohols: nucleophile (0.5 mmol); iodobenzene (0.5 mmol); Pd(OAc)_2_ (0.01 mmol); 1:2 Pd/TPP; base, Cs_2_CO_3_ (2.0 mmol); DMF (10 mL); *p*_CO_ = 1 bar; *T* = 100 °C; *t* = 24 h. Reaction conditions for amide esters: nucleophile (0.5 mmol); iodobenzene (1.0 mmol); Pd(OAc)_2_ (0.01 mmol); 1:2 Pd/TPP; base, Et_3_N (2.0 mmol); DMF (10 mL); *p*_CO_ = 1 bar; *T* = 100 °C; *t* = 24 h.

b *p*_CO_ = 60 bar; oxidation of the -SH group, not isolated.

c With 3 equiv of iodobenzene (isolated yields in parentheses).

To expand the scope of the reaction and demonstrating of applicability of the protocol, numerous related aliphatic nucleophiles were further subjected in the carbonylation with iodobenzene as a model aromatic iodide. The previously optimized conditions were applied for the synthesis of amide alcohols (**3j**–**r**) or amide esters (**4j**–**p**). It was revealed that most of the nucleophiles were capable to form the target compounds in moderate to good yields. Complete conversion of iodobenzene was observed in all reactions, but some benzoic acid formation decreased the chemoselectivities in some cases. In the presence of 2,2-dimethylethanolamine (**2j**) and phenylalaninol (**2n**), the yields of carbonylation products decreased compared to that of the parent ethanolamine (**2a**) (Table 1, entries 10 and 19). Secondary aminoalcohols such as *N*-methyl ethanolamine (**2k**) and cyclic pyrrolidine or piperidine derivatives (**2l** and **2m**) also successfully provided the targeted compounds. A moderate isolated yield was obtained in the aminocarbonylation of nortropine (**2o**). Alkoxycarbonylation cannot be achieved with this nucleophile, and the secondary alcohol moiety remained unchanged even under increased iodobenzene:nucleophile ratios and reaction times. Cysteamine as an analogous N,S-heterobifunctional nucleophile proved to be a suitable reactant in the amino- and thiocarbonylation of iodobenzene, providing the dicarbonylated compound (**4p**). An increased carbon monoxide pressure (60 bar) was applied in these reactions to achieve acceptable product yields. On the basis of GC-MS measurements, the selective formation of the amide thiol product was detected, as well, but the chromatographic isolation failed due to the oxidation of the thiol group.

**Scheme 2:**
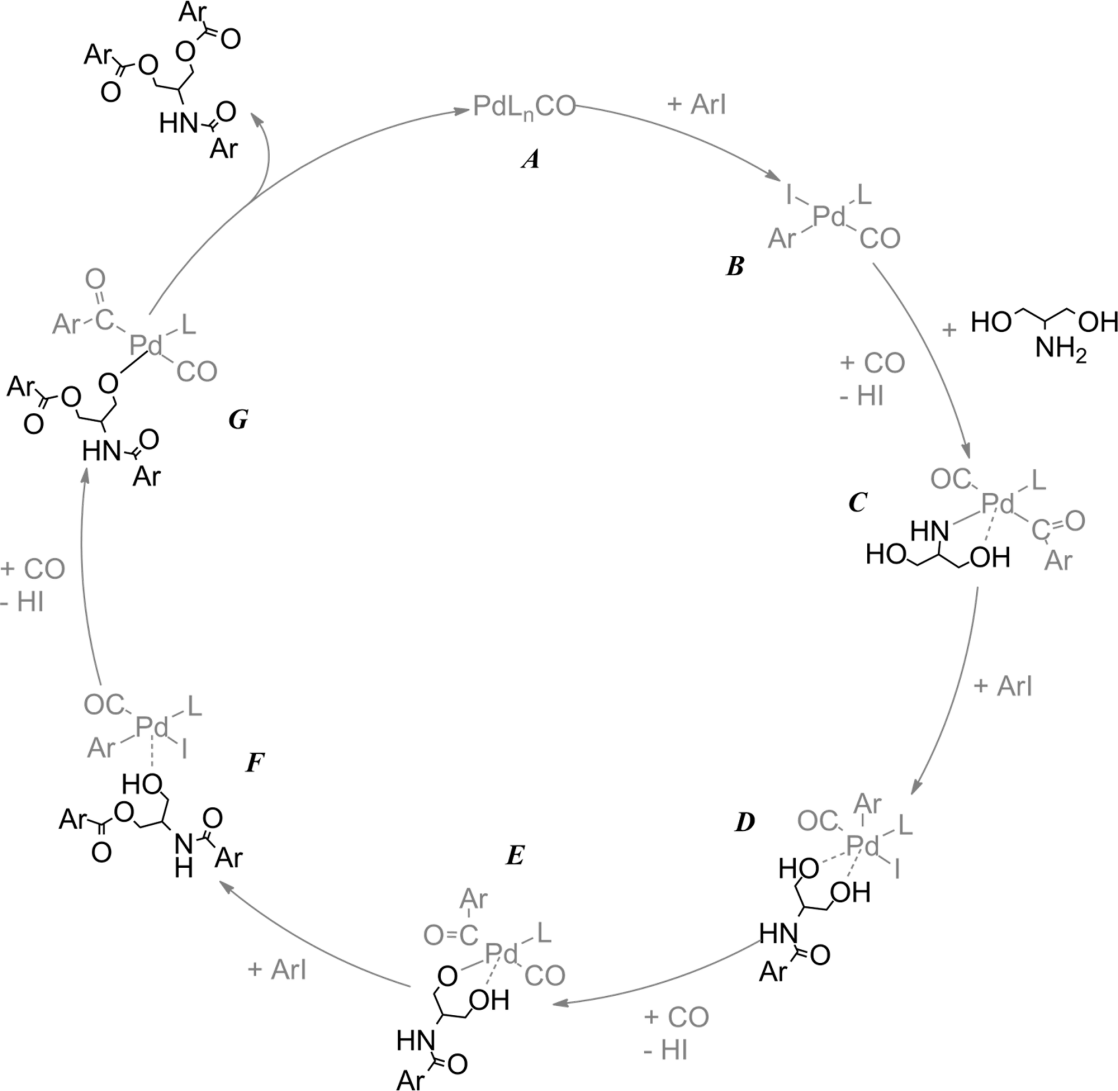
Proposed Simplified Catalytic Cycle of the Carbonylation of Iodobenzene in the Presence of Serinol as a Nucleophile (“rationalization” of the formation of amide diester product)

Additional experiments were performed with trifunctional serinol (**2q**) and 1,3-diaminopropanol (**2r**) using various substrate nucleophile ratios of 1:1, 2:1, and 3:1, with Et_3_N as the base. Unexpectedly, all of the nucleophile groups took part in the carbonylation reaction, providing the “tricarbonylated” compound as the main product, irrespective of the iodobenzene:nucleophile ratio. To explain this unusual behavior, a mechanism is proposed for the carbonylation reaction of iodobenzene in the presence of serinol as the model trifunctional nucleophile (Scheme 2). The initial step is the oxidative addition of iodoarene to the Pd^0^ carbonyl complex (**A** and **B**). In the next three steps, an acyl-amine complex is forming (**C**), by the insertion of CO into the Pd–Ar bond, followed by the coordination of serinol and the removal of HI (supported by the base). After the formation of the amide functionality, the product, as a chelating diol, remains in the coordination sphere of palladium, and the reductive elimination step allows the oxidative addition of another (“second”) aryl iodide (**D**). The reaction continues by the alkoxycarbonyation rounds with the same catalytic steps [CO insertion, removal of HI (**E**), and reductive elimination (**F**)] to form the amide ester and, by activating the “third” iodobenzene, amide diester compounds. Similar to the amide diol “intermediate”, the amide ester alcohol is still a potential ligand with various coordination possibilities (monodentate or chelating). The coordinative feature of trifunctional nucleophiles can explain the exclusive formation of amide diester (and diamide ester) product(s), formed by the serial carbonylation of iodobenzene.

## Conclusions

In conclusion, the selective amino- and alkoxycarbonylation reactions of iodobenzene and related substrates were achieved in the presence of heterobifunctional N,O-nucleophiles. Using triethylamine as the base and ethanolamine as the model reaction partner, the dominance of amide ester formation was observed even at a nucleophile:substrate ratio of 1:1. The level of amide alcohol selectivity can be successfully increased by the application of Cs_2_CO_3_ as the base. Various heteroaromatic substrates and related nucleophiles were tested under optimized reaction conditions to form the targeted compounds in moderate to good yields. Competitive reactions of substituted aryl iodides showed that electron-withdrawing groups increase the rate of aminocarbonylation reaction, resulting in a significant change in product selectivity. Additional experiments were conducted with serinol and 1,3-diamino propanol as exemplar trifunctional nucleophiles with various iodobenzene amounts. The results showed increased affinity for amide diester and diamide ester formation, and these phenomena were explained by mechanistic considerations.

## Data Availability

The data underlying this study are available in the published article and its Supporting Information.

## Supporting Information Available

General information about the screening of the carbonylation reaction, details of screening of the *para* substituent effect and competitive reactions of iodobenzene and aminoethanol, general information about product isolation, general procedure for the hydrolysis of amides, MS data of ring-substituted amides (**3b–f**) and amide esters (**4b–f**), and characterization and spectroscopic data (^1^H, ^13^C, and MS) of prepared compounds (PDF)

## References

[ref1] aMika L. T.; Ungváry F. Hydroformylation - Homogeneous. In Encyclopedia of Catalysis, 2nd ed.; Horváth I. T., Ed.; John Wiley & Sons. 10.1002/0471227617.eoc108.pub2

[ref2] aKiss G. Palladium-catalyzed Reppe carbonylation. Chem. Rev. 2001, 101, 3435–3456. 10.1021/cr010328q. 11840990

[ref3] aDong K.; Fang X.; Gülak S.; Franke R.; Spannenberg A.; Neumann H.; Jackstell R.; Beller M. Highly Active and Efficient Catalysts for Alkoxycarbonylation of Alkenes. Nat. Commun. 2017, 8, 14117 10.1038/ncomms14117. 28120947 PMC5288498

[ref4] aBrennführer A.; Neumann H.; Beller M. Palladium-Catalyzed Carbonylation Reactions of Aryl Halides and Related Compounds. Angew. Chem., Int. Ed. Engl. 2009, 48, 4114–4133. 10.1002/anie.200900013. 19431166

[ref5] aSeni A. A.; Kollár L.; Mika L. T.; Pongrácz P. Rhodium-Catalysed Aryloxycarbonylation of Iodo-Aromatics by 4-Substituted Phenols with Carbon Monoxide or Paraformaldehyde. Mol. Catal. 2018, 457, 67–73. 10.1016/j.mcat.2018.07.021.

[ref6] aHussain N.; Chhalodia A. K.; Ahmed A.; Mukherjee D. Recent Advances in Metal-Catalyzed Carbonylation Reactions by Using Formic Acid as CO Surrogate. Chem. Select. 2020, 5, 11272–11290. 10.1002/slct.202003395.

[ref7] aCao J.; Zheng Z.-J.; Xu Z.; Xu L.-W. Transition-Metal-Catalyzed Transfer Carbonylation with HCOOH or HCHO as Non-Gaseous C1 Source. Coord. Chem. Rev. 2017, 336, 43–53. 10.1016/j.ccr.2017.01.005.

[ref8] aTukacs J. M.; Marton B.; Albert E.; Tóth I.; Mika L. T. Palladium-Catalyzed Aryloxy- and Alkoxycarbonylation of Aromatic Iodides in γ-Valerolactone as Bio-Based Solvent. J. Organomet. Chem. 2020, 923, 121407 10.1016/j.jorganchem.2020.121407.

[ref9] aWu X.-F.; Neumann H.; Beller M. Palladium-catalyzed carbonylative coupling reactions between Ar-X and carbon nucleophiles. Chem. Soc. Rev. 2011, 40, 4986–5009. 10.1039/c1cs15109f. 21792459

[ref10] aBurhardt M. N.; Ahlburg A.; Skrydstrup T. Palladium-Catalyzed Thiocarbonylation of Aryl, Vinyl, and Benzyl Bromides. J. Org. Chem. 2014, 79, 11830–11840. 10.1021/jo5009965. 24919457

[ref11] Schmidt M.; Urban C.; Schmidt S.; Schomäcker R. Palladium-Catalyzed Hydroxycarbonylation of 1-Dodecene in Microemulsion Systems: Does Reaction Performance Care about Phase Behavior?. ACS Omega 2018, 3, 13355–13364. 10.1021/acsomega.8b01708. 31458049 PMC6644908

[ref12] Darbem M. P.; Esteves H. A.; Oliveira I. M.; Pimenta D. C.; Stefani H. A. Palladium-Catalyzed Thio- and Selenocarbonylation of 2-Iodoglycals. Chem. Catal. Chem. 2020, 12, 576–583. 10.1002/cctc.201901403.

[ref13] aZhu X.; Yu L.; Zhang M.; Xu Z.; Yao Z.; Wu Q.; Du X.; Li J. Design, Synthesis and Biological Activity of Hydroxybenzoic Acid Ester Conjugates of Phenazine-1-Carboxylic Acid. Chem. Cent. J. 2018, 12, 111 10.1186/s13065-018-0478-2. 30386935 PMC6768031

[ref14] aXu T.; Sha F.; Alper H. Highly Ligand-Controlled Regioselective Pd-Catalyzed Aminocarbonylation of Styrenes with Aminophenols. J. Am. Chem. Soc. 2016, 138, 6629–6635. 10.1021/jacs.6b03161. 27159663

[ref15] Xu T.; Alper H. Pd-catalyzed chemoselective carbonylation of aminophenols with iodoarenes: alkoxycarbonylation vs aminocarbonylation. J. Am. Chem. Soc. 2014, 136, 16970–16973. 10.1021/ja508588b. 25283812

[ref16] Perry R. J.; Wilson B. D.; Miller R. J. Synthesis of 2-arylbenzoxazoles via the palladium-catalyzed carbonylation and condensation of aromatic halides and o-aminophenols. J. Org. Chem. 1992, 57, 2883–2887. 10.1021/jo00036a025.

[ref17] Kebede E.; Tadikonda R.; Nakka M.; Inkollu B.; Vidavalur S. Construction of Benzimidazoles and Benzoxazoles through the Molybdenum-Mediated Carbonylation of Aryl Halides. Eur. J. Org. Chem. 2015, 2015, 5929–5933. 10.1002/ejoc.201500803.

[ref18] Lin W.; Wu W.; Selvaraju M.; Sun C. One-pot synthesis of benzazoles and quinazolinones via iron pentacarbonyl mediated carbonylation of aryl iodides under microwave irradiation. Org. Chem. Front. 2017, 4, 392–397. 10.1039/C6QO00733C.

[ref19] Carrilho M. B.; Almeida A. R.; Kiss M.; Kollár L.; Skoda-Földes R.; Da̧brovski J. M.; Moreno M. J. S. M.; Pereira M. M. One-Step Synthesis of Dicarboxamides through Pd-Catalysed Aminocarbonylation with Diamines as N-Nucleophiles. Eur. J. Org. Chem. 2015, 2015, 1840–1847. 10.1002/ejoc.201403444.

[ref20] Mikle G.; Noveczky P.; Mahó S.; Kollár L. Palladium-catalysed amino-vs. Alkoxycarbonylation of iodoalkenes using bifunctional N,O-nucleophiles. Tetrahedron 2021, 85, 132050 10.1016/j.tet.2021.132050.

