# Supplementary material for: Palladium-Catalyzed Selective Amino- and Alkoxycarbonylation of Iodoarenes with Aliphatic Aminoalcohols as Heterobifunctional O,N-Nucleophiles
Source: J Org Chem. 2023 Apr 13;88(8):5172–9. doi: 10.1021/acs.joc.2c02712 (PMC10127279; doi:10.1021/acs.joc.2c02712)
Supplement: Supplementary file 1 — jo2c02712_si_001.pdf [file jo2c02712_si_001.pdf]

Supporting Information

Palladium-catalyzed selective amino- and alkoxycarbonylation of iodoarenes with aliphatic aminoalcohols as heterobifunctional O,N-nucleophiles

László Kollár,[a,b] Attila Takács,[b] Csilla Molnár,[b] Andrew Kovács,[b]

László T. Mika,[c,*] and Péter Pongrácz [b,*]

[a] ELKH-PTE Research Group for Selective Chemical Syntheses, Ifjúság u. 6., Pécs, H-7624 Hungary and János Szentágothai Research Centre, University of Pécs, Ifjúság u. 20., Pécs, H-7624 Hungary

[b] Department of General and Inorganic Chemistry, University of Pécs, Ifjúság u. 6., Pécs, Hungary, H-7624.

[c] Department of Chemical and Environmental Process Engineering, Faculty of Chemical Technology and Biotechnology, Budapest University of Technology and Economics, Műegyetem rkp. 3., H–1111 Budapest, Hungary

*e-mail László T. Mika: laszlo.t.mika@edu.bme.hu

*e-mail Péter Pongrácz: pongracz@gamma.ttk.pte.hu

Table of contents

1. **Typical carbonylation experiments performed under atmospheric CO pressure** **S2**
2. **Screening of the reaction conditions for carbonylation of iodobenzene and aminoethanol** **S2**
3. **Screening of the *para*-substituent effect and competitive reactions** **S3**
4. **General procedure of product isolation/purification** **S4**
5. **MS-data table of ring substituted amides (3b-f) and amide-esters (4b-f)** **S4**
6. **General procedure for the hydrolysis of amide-esters to amide-alcohols and carboxylic acids** **S4**
7. **Characterisation and spectroscopic data (^{1}H, ^{13}C, MS) of prepared compounds** **S5**
8. **^{1}H, $^{13}C\{^{1}H\}$, MS spectrum of the isolated compounds** **S11**

1. **Typical carbonylation experiments performed under atmospheric CO pressure**

   a. **for the synthesis of amide-alcohols**

Catalyst precursor [$Pd(OAc)_2$] (2 mol %, 2.25 mg) and triphenylphosphine (4 mol % , 5.24 mg), were placed in a three-necked flask and refilled with argon gas three times. DMF (10 mL), iodobenzene (0.5 mmol. 56 µL), ethanolamine (0.5 mmol, 30 µL) and Et_3N (2.0 mmol, 278 µL) were transferred to the flask. The atmosphere was changed to carbon monoxide (1 bar). The mixture was heated with heat-on block system (100 ˚C) and stirred with magnetic stirrer for 24 hours. After the reaction was completed, the mixture was cooled, filtered and immediately analysed by GC and GC–MS. GC yields were determined by using ethylparabene as external standard.

   b. **for the sysnthesis of amide-esters**

Catalyst precursor [$Pd(OAc)_2$] (2 mol %, 2.25 mg) and triphenylphosphine (4 mol % , 5.24 mg), were placed in a three-necked flask and refilled with argon gas three times. DMF (10 mL), iodobenzene (0.5 mmol. 56 µL), ethanolamine (1.0 mmol, 60 µL) and Cs_2CO_3 (2.0 mmol, 651.6 mg) were transferred to the flask. The atmosphere was changed to carbon monoxide (1 bar). The mixture was heated with heat-on block system (100 ˚C) and stirred with magnetic stirrer for 24 hours. After the reaction was completed, the mixture was cooled, filtered and immediately analysed by GC and GC–MS. GC yields were determined by using ethylparabene as external standard.

2. **Screening of the reaction conditions for carbonylation of iodobenzene and aminoethanol**

For optimisation reactions, 12 places parallel reaction station was used. Experimental setting is detailed in the following tables. Stock solutions of unvaried ingredients were prepared and divided into the vessels, than varied reagents were measured separately to the vessels.

Table S1. Experimental setup of round I.

Vessel	Iodobenzene [mmol]	Aminoethanol [mmol]	$Pd(OAc)_2$ [mmol]	Ligand [0.008 mmol/P donor]	Base [0.4 mmol]	Solvent [2 mL]		Measured separately into the vessels		
1	0.2	0.2	0.004	TPP	Et_3N	DMF		TPP 2 mg	Et_3N 55 µL	
2	0.2	0.2	0.004	TPP	Cs_2CO_3	DMF		TPP 2 mg	Cs_2CO_3 130 mg	
3	0.2	0.2	0.004	TPP	Na_2CO_3	DMF		TPP 2 mg	Na_2CO_3 44 mg	
4	0.2	0.2	0.004	TPP	TMG	DMF		TPP 2 mg	DABCO 45 mg	
5	0.2	0.2	0.004	DPPB	Et_3N	DMF		DPPB 1.7 mg	Et_3N 55 µL	
6	0.2	0.2	0.004	DCyPB	Et_3N	DMF		DCyPB 1.8 mg	Et_3N 55 µL	
7	0.2	0.2	0.004	DPPF	Et_3N	DMF		DPPF 2.2 mg	Et_3N 55 µL	
8	0.2	0.2	0.004	Xantphos	Et_3N	DMF		Xantphos 2.3 mg	Et_3N 55 µL	
9	0.2	0.2	0.004	-	Et_3N	DMF		-	Et_3N 55 µL	
10	0.2	0.2	0.004	TPP	Et_3N	MeCN		-	Et_3N 55 µL	MeCN 2 mL
11	0.2	0.2	0.004	TPP	Et_3N	NMP		-	Et_3N 55 µL	NMP 2 mL
12	0.2	0.2	0.004	TPP	Et_3N	GVL		-	Et_3N 55 µL	GVL 2 mL

Stock solution I.	Iodobenzene	Aminoethanol	$Pd(OAc)_2$	DMF
divided into 9 parts	1.8 mmol	1.8 mmol	0.036 mmol	18 mL
Vessel 1-9	200 µL	110 µL	8 mg	

Stock solution II.	Iodobenzene	Aminoethanol	$Pd(OAc)_2$	TPP	CH_2Cl_2
divided into 3 part	0.6 mmol	0.6 mmol	0.012 mmol	0.024 mmol	6 mL
Vessel 10-12	67 µL	37 µL	2.7 mg	6.2 mg	

In vessel 10-12 dichloromethane was used as solvent to transfer the unvaried reagents (2-2 mL in each vessels). The solvent was then evaporated under vacuum and 2 mL of MeCN, NMP and GVL was added separately (to vessels 10-12, respectively). The atmosphere of the vessels was changed to carbon monoxide (1 bar) and reaction mixtures were heated with heat-on block system to 100 °C and stirred

with magnetic stirrer for 24 hours. After the reactions were completed, the mixtures were cooled, filtered and immediately analysed by GC and GC–MS. GC yields were determined by using ethylparabene as external standard.

Table S2. Experimental setup of round II.

Vessel	Iodobenzene [mmol]	Aminoethanol [mmol]	$Pd(OAc)_2$ [mmol]	Ligand [0.008 mmol/P donor]	Base [0.4 mmol]	Solvent [2 mL]		Measured separately into the vessels		
1	0.2	0.2	0.004 mmol	TPP	Et_3N	DMF		TPP 2 mg	Et_3N 55 µL	
2	0.2	0.2	0.004 mmol	TPP	Na_2CO_3	DMF		TPP 2 mg	Na_2CO_3 44 mg	
3	0.2	0.2	0.004 mmol	Cy_3P	Et_3N	DMF		Cy_3P 2.2 mg	Et_3N 55 µL	
4	0.2	0.2	0.004 mmol	DPEPhos	Et_3N	DMF		DPEPhos 2.2 mg	Et_3N 55 µL	
5	0.2	0.2	0.004 mmol	DtBuPBenz	Et_3N	DMF		DtBuPBenz 1.6 mg	Et_3N 55 µL	
6	0.2	0.2	0.004 mmol	Cy_3P	Cs_2CO_3	DMF		Cy_3P 2.2 mg	Cs_2CO_3 130 mg	
7	0.2	0.2	0.004 mmol	DPEPhos	Cs_2CO_3	DMF		DPEPhos 2.2 mg	Cs_2CO_3 130 mg	
8	0.2	0.2	0.004 mmol	DtBuPBenz	Cs_2CO_3	DMF		DtBuPBenz 1.6 mg	Cs_2CO_3 130 mg	
9	0.4	0.2	0.004 mmol	TPP	Et_3N	DMF		TPP 2 mg	Et_3N 55 µL	I-Ph 22 µL
10	0.4	0.2	0.004 mmol	TPP	Cs_2CO_3	DMF		TPP 2 mg	Cs_2CO_3 130 mg	I-Ph 22 µL
11	0.4	0.2	0.004 mmol	Xantphos	Et_3N	DMF		Xantphos 2.3 mg	Et_3N 55 µL	I-Ph 22 µL
12	0.4	0.2	0.004 mmol	Xantphos	Cs_2CO_3	DMF		Xantphos 2.3 mg	Cs_2CO_3 130 mg	I-Ph 22 µL

Stock solution I.	Iodobenzene	Aminoethanol	$Pd(OAc)_2$	DMF
divided into 12 parts	2.4 mmol	2.4 mmol	0.048 mmol	24 mL
Vessel 1-12	270 µL	147 µL	10.85 mg	

Stock solution of iodobenzene, aminoethanol, palladium(II) acetate and DMF was transferred to the vessels. Variable reagents (ligands and base) were measured separately directly to the vessels. Additional iodobenzene was measured into vessels 9-12 providing the substrate/nucleophile ratio 2:1. The atmosphere of the vessels was changed to carbon monoxide (1 bar) and reaction mixtures were heated with heat-on block system to 100 ^{o}C and stirred with magnetic stirrer for 24 hours. After the reactions were completed, the mixtures were cooled, filtered and immediately analysed by GC and GC–MS. GC yields were determined by using ethylparabene as external standard.

3. Screening of the *para*-substituent effect and competitive reactions

For these reactions, 12 places parallel reaction station was used. Experimental setting is detailed in the following table. Stock solution of unvaried ingredients was prepared and divided into the vessels, then varied reagents were measured separately to the vessels.

Table S3. Experimental setup

Vessel	Aminoethanol [mmol]	$Pd(OAc)_2$ [mmol]	Ligand [0.008 mmol/P donor]	Base [0.4 mmol]	Solvent [2 mL]	Substrate 1 [0.2 mmol]	Substrate 2 [0.2 mmol]		Measured separately into vessels	
									Substrate 1	Substrate 2
1	0.2	0.004 mmol	TPP	Et_3N	DMF	Iodobenzene	-		23 µL	-
2	0.2	0.004 mmol	TPP	Et_3N	DMF	4-Me-Iodobenzene	-		43.6 mg	-
3	0.2	0.004 mmol	TPP	Et_3N	DMF	4-OMe-Iodobenzene	-		46.8 mg	-
4	0.2	0.004 mmol	TPP	Et_3N	DMF	4-Cl-Iodobenzene	-		47.8 mg	-
5	0.2	0.004 mmol	TPP	Et_3N	DMF	4-CF_3-Iodobenzene	-		29 µL	-
6	0.2	0.004 mmol	TPP	Et_3N	DMF	3,5-bis-CF_3-iodobenzene	-		35 µL	-
7	0.2	0.004 mmol	TPP	Et_3N	DMF	4-Me-Iodobenzene	4-OMe-Iodobenzene		43.6 mg	46.8 mg
8	0.2	0.004 mmol	TPP	Et_3N	DMF	4-Me-Iodobenzene	4-Cl-Iodobenzene		43.6 mg	47.8 mg
9	0.2	0.004 mmol	TPP	Et_3N	DMF	4-Me-Iodobenzene	4-CF_3-Iodobenzene		43.6 mg	29 µL
10	0.2	0.004 mmol	TPP	Et_3N	DMF	4-OMe-Iodobenzene	4-Cl-Iodobenzene		46.8 mg	47.8 mg
11	0.2	0.004 mmol	TPP	Et_3N	DMF	4-OMe-Iodobenzene	4-CF_3-Iodobenzene		46.8 mg	29 µL
12	0.2	0.004 mmol	TPP	Et_3N	DMF	4-Cl-Iodobenzene	4-CF_3-Iodobenzene		47.8 mg	29 µL

Stock solution I.	Aminoethanol	$Pd(OAc)_2$	TPP	Et_3N	DMF
divided into 12 parts	2.4 mmol	0,048 mmol	0.096 mmol	4.8 mmol	24 mL
	147 µL	10.8 mg	25.2 mg	668 µL	

In vessels 1-6 aminoethanol-substrate ratio was kept at 1:1. In experiments 7-12 aminoethanol substrate ratio was increased to 1:2 by adding 0.2 mmol of both substrates. The atmosphere of the vessels was changed to carbon monoxide (1 bar) and reaction mixtures were heated with heat-on block system to 100 °C and stirred with magnetic stirrer for 24 hours. After the reactions were completed, the mixtures were cooled, filtered and immediately analysed by GC and GC–MS. GC yields were determined by using ethylparabene as external standard.

4. General procedure of product isolation/purification

The reaction mixture was concentrated under vacuum and the crude product was purified by column chromatography on silica gel using chloroform/ethyl acetate eluent to afford the corresponding products.

5. MS-data table of ring substituted amides (3b-f) and amide-esters (4b-f)

Table S4. MS data of ring substituted derivatives

Amides		
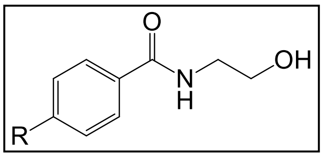		
Compound	***R***	***MS m/z (rel. int.)***
**3b**	Me	179 (3, M^+), 161 (10), 136 (16), 119 (100), 91 (36), 65 (14), 39 (3).
**3c**	OMe	195 (6, M+), 177 (5), 151 (17), 135 (100), 107 (12), 92 (9), 77 (17), 64 (5).
**3d**	Cl	199 (1, M^+), 181 (11), 156 (21), 139 (100), 111 (35), 75 (20), 50 (5).
**3e**	CF_3	233 (1, M^+), 215 (12), 202 (12), 190 (28), 173 (100), 145 (51), 95 (8), 75 (5), 44 (3).
**3f**	3,5-CF_3	283 (9), 270 (15), 258 (26), 241 (100), 213 (37), 194 (4), 163 (7), 144 (4).
Amide-Esters		
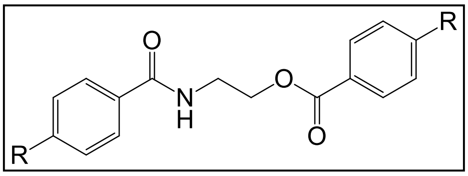		
Compound	***R***	***MS m/z (rel. int.)***
**4b**	Me	254 (3), 178 (4), 161 (19), 119 (100), 91 (22), 65 (6).
**4c**	OMe	286 (1), 194 (3), 177 (17), 135 (100), 107 (7), 92 (5), 77 (10).
**4d**	Cl	294 (3), 198 (5), 181 (23), 139 (100), 111 (24), 75 (10), 50 (2).
**4e**	CF_3	386 (2), 363 (2), 232 (5), 215 (23), 173 (100), 145 (30), 95 (4).
**4f**	3,5-CF_3	300 (3), 283 (19), 270 (5), 241 (100), 213 (21), 194 (3), 163 (4).

Mixed products cannot be isolated by chromatography.

6. General procedure for the hydrolysis of amide-esters to amide-alcohols and carboxylic acids

The crude reaction mixture (10 mL) of carbonylation reactions was evaporated under vacuum to remove DMF. The residue was dissolved in ethanol (4 mL) and deionized water (2 mL), then sodium hydroxide was added (~100 mg). The solution was heated with heat-on block system to 80°C and stirred for 2 hours. After cooling down the solution with ice bath concentrated HCl was added to reach

acidic pH. The solution was washed with diethyl ether (10 mL) two times and the organic phase was dried over anhydrous sodium sulphate. After filtration the solvent was evaporated and the product was analysed by GC-MS.

7. Characterization, ^{1}H, ^{13}C NMR and MS data of the synthesized compounds

Multiplets were assigned as s (singlet), br s (broad singlet), d (doublet), t (triplet), m (multiplet), dd (doublet of doublet).

N-(2-hydroxyethyl)-benzamide **(3a)**

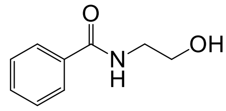

Reference [1]; White solid; R_f ($CHCl_3$/IPA = 9:1) 0.83; Yield: 56 % (46.2 mg); 1H NMR ($CDCl_3$, 500 MHz): δ 7.80 (d, 2H, J = 7.0 Hz), 7.51 (m, 1H), 7.42 (m, 1H), 6.89 (s, 1H), 3.83 (t, 2H, J = 5.0 Hz), 3.63 (m, 2H), 3.20 (s, 1H). 13C{1H} NMR ($CDCl_3$, 125 MHz): δ 168.6, 134.1, 131.6, 128.6, 127.0, 62.2, 42.9. MS m/z (rel int.): 165 (1, M^+)147 (11), 134 (9), 122 (24), 105 (100), 77 (48), 51 (14).

2-Benzamidoethyl benzoate **(4a)**

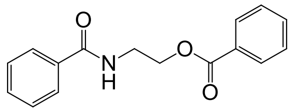

Reference [2];White solid; R_f ($CHCl_3$/EtAc = 9:1) 0.51; Yield: 69 % (92.8 mg); 1H NMR ($CDCl_3$, 500 MHz): δ 8.08 (d, 2H, J = 7.3 Hz), 7.81 (d, 2H, J = 7.1 Hz), 7.61 (t, 1H, J = 7.4 Hz), 7.53 (t, 1H, J = 7.4 Hz), 7.53-7.46 (m, 4H), 6.69 (s, 1H), 4.59 (t, 2H, J = 5.2 Hz), 3.90 (dt, 2H, J = 5.2 Hz, J = 5.5 Hz). 13C{1H} NMR ($CDCl_3$, 125 MHz): δ 167.6, 167.1, 134.3, 134.0, 133.3, 131.6, 129.7, 128.6, 128.5, 126.9, 63.9, 39.8. MS m/z (rel int.): 226 (3), 164 (4), 147 (22), 105 (100), 77 (31), 51 (7).

N-*(2-Hydroxyethyl)nicotinamide* **(3g)**

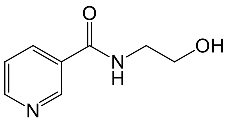

Reference [3]; Yellow solid; R_f ($CHCl_3$/IPA = 9:1) 0.24; Yield: 56 % (46.5 mg); 1H NMR ($CDCl_3$, 500 MHz): δ 9.10 (s, 1H), 8.74 (d, 1H, J = 3.4 Hz), 8.21 (d, 1H, J = 7.8 Hz), 7.44-7.41 (m, 1H), 7.03 (s, 1H), 3.90-3.88 (m, 2H), 3.70-3.67 (m, 2H). 13C{1H} NMR ($CDCl_3$, 125 MHz): δ 166.2, 151.8, 147.9, 135.7, 130.2, 123.6. m/z (rel int.): 148 (80), 118 (100), 106 (9), 92 (9), 91 (8), 78 (30), 51 (16).

2-[(3-Pyridinylcarbonyl)amino]ethyl 3-pyridinecarboxylate **(4g)**

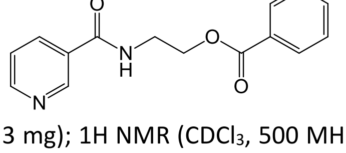

Reference [4];White solid; Rf ($CHCl_3$/EtAc = 9:1) 0.61; Yield: 77 % (104.3 mg); 1H NMR ($CDCl_3$, 500 MHz): δ 9.20 (s, 1H), 9.10 (s, 1H), 8.73 (d, 1H, J = 3.4 Hz), 8.67 (d, 1H, J = 3.4 Hz), 8.30 (d, 1H, J = 7.8 Hz), 8.21 (d, 1H, J = 7.8 Hz), 7.53 (s, 1H,), 7.39-7.35 (m, 2H), 4.58 (t, 2H, J = 5.2 Hz), 3.90-3.86 (m, 2H) 13C{1H} NMR ($CDCl_3$, 125 MHz): δ 165.9, 165.5, 153.5, 152.0, 150.9, 148.4, 137.3, 135.4, 129.9, 125.8, 123.5, 123.4, 64.1, 39.2. MS m/z (rel int.): 271 (1, M^+), 228 (2), 165 (17), 148 (29), 135 (18), 124 (7), 106 (100), 78 (48), 51 (15).

***N-(2-Hydroxyethyl)-2-thiophenecarboxamide* (3h)** 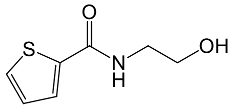

Reference [5];White solid; R_f ($CHCl_3$/IPA = 9:1) 0.48; Yield: 39 % (33.3 mg); 1H NMR ($CDCl_3$, 500 MHz): δ 7.56 (d, 1H, J = 3.7 Hz), 7.49 (d, 1H, J = 4.9 Hz), 7.09-7.07 (m, 1H), 6.82 (s, 1H), 3.83 (t, 2H, J = 4.9 Hz), 3.60, (dt, 2H, J = 4.9 Hz). 13C{1H} NMR ($CDCl_3$, 125 MHz): δ 163.0, 138.6, 130.2, 128.4, 127.7, 62.0, 42.7. MS m/z (rel int.): 171 (3, M^+), 153 (4), 140 (7), 138 (7), 128 (16), 120 (7), 111 (100), 83 (8), 39 (13).

***2-[(2-Thienylcarbonyl)amino]ethyl 2-thiophenecarboxylate* (4h)** 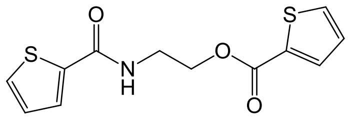

Reference [6]; White solid; R_f ($CHCl_3$/EtAc = 9:1) 0.29; Yield: 59 % (82.9 mg); 1H NMR ($CDCl_3$, 500 MHz): δ 7.84 (d, 1H, J = 3.7 Hz), 7.59 (d, 1H, J = 4.9 Hz), 7.55 (d, 1H, J = 3.7 Hz), 7.49 (d, 1H, J = 4.9 Hz), 7.13-7.11 (m, 1H), 7.09-7.07 (m, 1H), 6.65 (s, 1H), 4.52 (t, 2H, J = 5.4 Hz),3.80 (dt, 2H, J = 5.4 Hz). 13C{1H} NMR ($CDCl_3$, 125 MHz): δ 162.5, 162.1, 145.8, 138.7, 134.0, 132.9, 130.1, 128.2, 127.9, 127.6, 63.9, 39.6. MS m/z (rel int.): 280 (1, M^+), 238 (3), 170 (3), 153 (20), 140 (5), 120 (14), 111 (100), 83 (6), 39 (9).

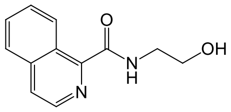

***N-(2-Hydroxyethyl)-1-isoquinolinecarboxamide* (3i)**

Yellow oil; R_f ($CHCl_3$/IPA = 9:1) 0.61; Yield: 83 % (89.6 mg); 1H NMR ($CDCl_3$, 500 MHz): δ 9.53 (d, 1H, J = 8.4 Hz), 8.60 (s, 1H), 8.49 (d, 1H, J = 5.5 Hz), 7.88 (d, 1H, J = 8.4 Hz), 7.84 (d, 1H, J = 5.5 Hz), 7.78-7.75 (m, 1H), 7.73-7.70 (m, 1H), 3.93 (t, 2H, J = 5.0 Hz), 3.74 (dt, 2H, J = 5.0 Hz). 13C{1H} NMR ($CDCl_3$, 125 MHz): δ 166.9, 148.1, 139.7, 137.6, 130.8, 128.8, 127.9, 127.0, 126.8, 124.6, 62.6, 42.7. MS m/z (rel int.): 216 (4, M^+), 198 (4), 185 (44), 156 (23), 128 (100), 101 (15), 77 (7). HRMS (ESI-orbitrap) m/z: $[M + H]^+$ Calcd for C12H13N2O2 217.0972; Found 217.0968

***N-(2-hydroxyl-1,1-dimethylethyl)-benzamide* (3j)** 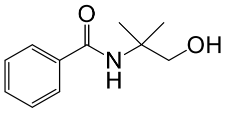

Reference [7]; White solid; R_f ($CHCl_3$) 0.25; Yield: 20 % (19.3 mg); 1H NMR ($CDCl_3$, 500 MHz): δ 7.72 (d, 2H, J = 7.6 Hz), 7.47 (t, 1H, J = 7.3 Hz), 7.39 (t, 2H, J = 7.6 Hz), 6.52 (s, 1H), 3.64 (d, 2H, J = 5.6 Hz), 1.40 (s, 6H). 13C{1H} NMR ($CDCl_3$, 125 MHz): δ 168.4, 135.0, 131.5, 128.5, 127.0, 70.6, 56.2, 24.4. MS m/z (rel int.): 177 (11), 176 (35), 148 (2), 105 (100), 77 (30), 51 (7).

***2-Benzamido-2-methylpropyl benzoate* (4j)** 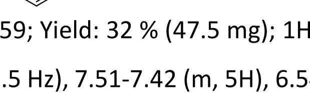

Reference [7];White solid; R_f ($CHCl_3$/EtAc = 9:1) 0.59; Yield: 32 % (47.5 mg); 1H NMR ($CDCl_3$, 500 MHz): δ (d, 2H, J = 7.3 Hz), 7.77 (d, 2H, J = 7.3 Hz), 7.59 (t, 1H, J = 7.5 Hz), 7.51-7.42 (m, 5H), 6.54 (s, 1H), 4.57 (s, 2H), 1.61 (s, 6H). 13C{1H} NMR ($CDCl_3$, 125 MHz): δ 167.1, 166.9, 135.3, 133.3, 131.3, 129.9, 129.7, 128.6, 128.5, 126.8, 70.3, 54.3, 24.0. MS m/z (rel int.): 226 (1), 175 (4), 162 (38), 122 (1), 105 (100), 77 (22), 51 (3), 42 (1).

***N-(2-hydroxyethyl)-N-methylbenzamide* (3k)** 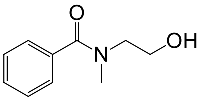

Reference [8]; Colorless oil; R_f ($CHCl_3$/IPA = 95:5) 0.32; Rotamers of amide product obtained in the NMR specta. Yield: 37 % (33.1 mg); 1H NMR ($CDCl_3$, 500 MHz): δ 7.43-7.41 (m, 5H), 3.88 (s, 1.6H), 3.71 (s, 1.6H), 3.64 (s, 0.6H), 3.39 (s, 0.6H), 3.11 (s, 1H), 3.03 (s, 2H). 13C{1H} NMR ($CDCl_3$, 125 MHz): δ 173.2, 130.0, 129.9, 128.4, 127.1, 60.9, 59.6, 53.1, 51.0, 38.8, 33.2. MS m/z (rel int.): 179 (3, M^+), 148 (16), 105 (100), 77 (39), 51 (11).

***2-(N-methylbenzamidoethyl) benzoate* (4k)** 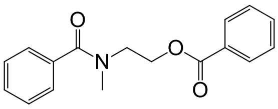

Yellow oil; R_f ($CHCl_3$/EtAc/Acetone = 3:5:2) 0.78; Rotamers of amide product obtained in the NMR specta Yield: 29 % (41.0 mg); 1H NMR ($CDCl_3$, 500 MHz): δ 8.08-8.02 (m, 2H), 7.60-7.58 (m, 1H), 7.47 (t, 2H, J = 7.7 Hz), 7.42-7.36 (m, 5H), 4.65 (s, 1.2H), 4.42 (s, 0.8H), 3.98 (s, 1.2H), 3.72 (s, 0.8H), 3.21 (s, 1.2H), 3.09 (s, 1.8H). 13C{1H} NMR ($CDCl_3$, 125 MHz): δ 171.9, 166.5, 136.2, 133.2, 129.7, 129.6, 128.5, 128.4, 126.9, 126.3, 62.4, 61.9, 50.1, 46.8, 38.7, 33.5. MS m/z (rel int.): 283 (1, M^+), 184 (2), 161 (15), 133 (5), 105 (100), 77 (36), 51 (9). HRMS (ESI-orbitrap) m/z: $[M + H]^+$ Calcd for C17H18NO3 284.1281; Found 284.1276

***1-(Benzoylpyrrolidin-2-yl)methanol* (3l)** 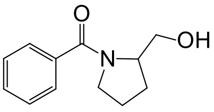

Reference [9]; Yellow oil; R_f ($CHCl_3$/EtAc = 7:3) 0.47; Yield: 40 % (41.0 mg); 1H NMR ($CDCl_3$, 500 MHz): δ 7.52-7.41 (m, 5H), 4.42-4.42 (m, 1H), 3.84-3.82 (m, 1H), 3.77-3.76 (m, 1H), 3.51-3.48 (m, 2H), 2.19-2.17 (m, 1H), 1.89-1.87 (m, 1H), 1.76-1.66 (m, 2H). 13C{1H} NMR ($CDCl_3$, 125 MHz): δ 172.2, 130.1, 130.0, 128.4, 127.0, 67.0, 61.5, 51.1, 28.5, 25.0. MS m/z (rel int.): 187 (3), 175 (4), 174 (25), 105 (100), 77 (31), 51 (6).

***N,O-Dibenzoyl-prolinol* (4l)** 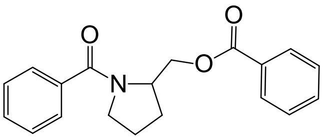

Yellow oil; R_f ($CHCl_3$/EtAc = 7:3) 0.69; Yield: 54 % (83.4 mg); 1H NMR (DMSO-d_6, 500 MHz): δ 8.02-8.00 (m, 2H), 7.67 (t, 1H, J = 7.3 Hz), 7.54 (t, 2H, J =7.3 Hz), 7.46-7.42 (m, 5H), 4.56-5.52 (m, 2H), 4.49-4.45 (m, 1H), 3.50-3.46 (m, 1H), 2.14-2.12 (m, 1H), 1.95-1.90 (m, 3H), 1.78-1.73 (m, 1H). 13C{1H} NMR (DMSO-d_6, 125 MHz): δ 169.4, 166.1, 137.6, 133.8, 130.2, 130.1, 129.7, 129.2, 128.7, 127.4, 64.9, 55.9, 50.3, 27.7, 25.0. MS m/z (rel int.): 204 (2), 187 (9), 186 (7), 174 (25), 159 (3), 105 (100), 77 (29), 51 (4). HRMS (ESI-orbitrap) m/z: $[M + H]^+$ Calcd for C19H20NO3 310.1438; Found 310.1432

(1-Benzoylpiperidin-2-yl)methanol **(3m)**

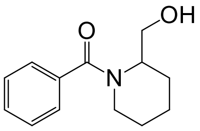

Reference [7]; Yellow oil; R_f ($CHCl_3$/EtAc = 9:1) 0.57; Yield: 75 % (82.1 mg); 1H NMR ($CDCl_3$, 500 MHz): δ 8.23 (d, 2H, J = 7.6 Hz), 7.58 (t, 1H, J = 7.6 Hz), 7.45 (t, 2H, J = 7.6 Hz), 4.72-4.70 (m, 2H), 3.72-3.70 (m, 1H), 3.59-3.57 (m, 1H), 2.97-2.90 (m, 1H), 2.09-2.06 (m, 1H), 2.01-1.95 (m, 3H), 1.62-1.55 (m, 1H). 13C{1H} NMR ($CDCl_3$, 125 MHz): δ 166.2, 133.6, 130.6, 128.6, 128.5, 63.8, 56.5, 45.3, 25.2, 21.8, 21.6. MS m/z (rel int.): 105 (10), 97 (10), 84 (100), 77 (10), 56 (8), 41 (3).

N-Benzoyl-2-piperidinemethyl benzoate **(4m)**

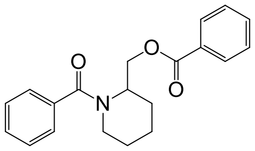

Reference [7]; White solid; R_f ($CHCl_3$/EtAc = 8:2) 0.61; Yield: 71 % (114.7 mg); 1H NMR ($CDCl_3$, 500 MHz): δ 8.05-8.01 (m, 2H), 7.56 (t, 1H, J = 7.3 Hz), 7.43 (t, 2H, J = 7.3 Hz), 7.37-7.38 (m, 5H), 5.36 (br s, 1H), 4.80-4.76 (m, 1H), 3.37-3.32 (m, 1H), 3.62 (br s, 1H), 3.14 (br s, 1H), 1.86-1.63 (m, 5H), 1.54-1.46 (m, 1H). 13C{1H} NMR ($CDCl_3$, 125 MHz): δ 171.3, 166.3, 136.4, 133.1, 129.8, 129.7, 129.4, 129.3, 128.4, 126.6, 62.2, 52.9, 47.2, 43.6, 37.8, 25.7, 19.7. MS m/z (rel int.): 218 (1), 201 (3), 188 (48), 173 (5), 105 (100), 77 (24), 51 (3).

N-Benzoylphenylalaninol **(3n)**

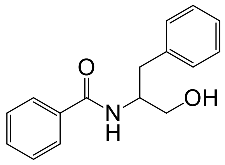

Reference [10]; White solid; R_f ($CHCl_3$/EtAc = 9:1) 0.12; Yield: 30 % (38.3 mg); 1H NMR (DMSO-d_6, 500 MHz): δ 8.16 (d, 1H, J = 8.4 Hz), 7.79 (d, 2H, J = 7.4 Hz), 7.50 (t, 1H, J = 7.4 Hz), 7.44 (t, 2H, J = 7.4 Hz), 7.27-7.25 (m, 4H), 7.17-7.14 (m, 1H), 4.19-4.14 (m, 1H), 3.51 (dd, 1H, J = 5.4 Hz, J = 10.7 Hz), 3.43 (dd, 1H, J = 6.15 Hz, J = 10.7 Hz) 2.96 (dd, 1H, J = 5.1 Hz, J = 13.5 Hz), 2.80 (dd, 1H, J = 9.1 Hz, J = 13.5 Hz). 13C{1H} NMR (DMSO-d_6, 125 MHz): δ 166.5, 139.9, 135.2, 131.4, 129.5, 128.6, 128.5, 127.7, 126.3. MS m/z (rel int.): 224 (5), 164 (31), 146 (4), 105 (100), 77 (30), 51 (4).

N-[1-[(Benzoyloxy)methyl]-2-phenylethyl] benzamide **(4n)**

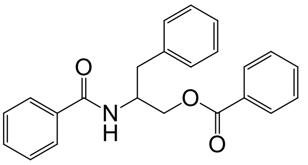

White solid; R_f ($CHCl_3$/EtAc = 9:1) 0.77; Yield: 42 % (75.4 mg); Melting point: 170 °C; 1H NMR ($CDCl_3$, 500 MHz): δ 8.08 (d, 2H, J = 7.6 Hz), 7.75 (d, 2H, J = 7.5 Hz), 7.61 (t, 1H, J = 7.5 Hz), 7.51-7.43 (m, 5H), 7.38-7.32 (m, 5H), 6.55 (d, 1H, J = 7.8 Hz), 4.79-4.77 (m, 1H), 4.48-4.46 (m, 2H), 3.18 (dd, 1H, J = 5.6 Hz, J = 13.5 Hz), 3.03 (dd, 1H, J = 8.0 Hz, J = 13.5 Hz). 13C{1H} NMR ($CDCl_3$, 125 MHz): δ 167.1, 166.9, 136.9, 134.4, 134.3, 133.3, 131.6, 129.7, 129.4, 128.8, 128.6, 128.5, 126.91, 126.87, 65.3, 50.8, 37.6. MS m/z (rel int.): 268 (2), 238 (8), 224 (2), 146 (53), 105 (100), 77 (27), 51 (3). HRMS (ESI-orbitrap) m/z: $[M + H]^+$ Calcd for C23H22NO3 360.1594; Found 360.1588

N-benzoyl nortropine **(3o)** 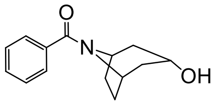

Reference [11]; White solid; R_f ($CHCl_3$/MeOH = 9:1) 0.17; Yield: 35 % (40.4 mg); 1H NMR ($CDCl_3$, 500 MHz): δ 7.49-7.46 (m, 2H), 7.44-7.40 (m, 3H), 4.83 (s, 1H), 4.22-4.20 (m, 1H), 4.07 (s, 1H), 2.35-2.24 (m, 3H), 2.02-2.00 (m, 3H), 1.89 (d, 1H, J = 14.2 Hz) 1.77 (d, 1H, J = 14.2 Hz). 13C{1H} NMR ($CDCl_3$, 125 MHz): δ 167.9, 136.4, 133.3, 128.4, 126.9, 65.1, 56.0, 51.0, 40.3, 38.7, 28.6, 27.3. MS m/z (rel int.): 231 (19), 186 (3), 126 (15), 105 (100), 77 (41), 68 (15), 51 (5).

N-(2-mercaptoethyl)-benzamide **(3p)** 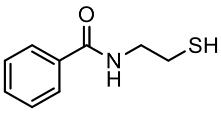

MS m/z (rel int.): 181 (6, M^+), 134 (9), 122 (41), 105 (100), 77 (50), 51 (15), 43 (6).

2-Benzamidoethyl benzcarbothioate **(4p)** 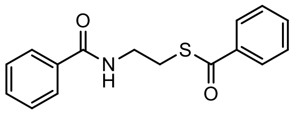

Reference [12]; Colorless solid; R_f ($CHCl_3$/EtAc = 9:1) 0.52; Yield: 71 % (101.2 mg); 1H NMR ($CDCl_3$, 500 MHz): δ 7.98 (d, 2H, J = 8.0 Hz), 7.80 (d, 2H, J = 7.5 Hz), 7.60 (t, 1H, J = 7.5 Hz), 7.51-7.41 (m, 5H), 6.92 (s, 1H), 3.77 (m, 2H), 3.38 (t, 2H, J = 6.5 Hz). 13C{1H} NMR ($CDCl_3$, 125 MHz): δ 192.7, 167.7, 136.7, 134.2, 133.8, 131.5, 128.7, 128.6, 127.3, 127.0, 40.4, 28.6. MS m/z (rel int.): 226 (10), 180 (13), 148 (8), 105 (100), 77 (31), 51 (6).

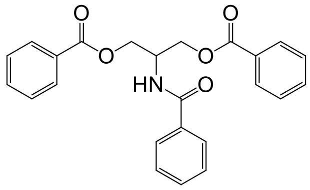

2-(Benzoylamino)propane-1,3-diyl dibenzoate **(3q)**

White solid; R_f ($CHCl_3$/EtAc = 8:2) 0.56; Yield: 57% (114.9 mg); Melting point: 157 °C; 1H NMR ($CDCl_3$, 500 MHz): δ 8.07 (d, 4H, J = 7.3 Hz), 7.81 (d, 2H, J = 7.3 Hz), 7.60 (t, 2H, J = 7.4 Hz), 7.53 (t, 1H, J = 7.4 Hz), 7.48-7.45 (m, 6H), 6.88 (d, 1H, J = 7.8 Hz), 4.99-4.96 (m, 1H), 4.75 (dd, 2H, J = 5.6 Hz, J = 11.5 Hz), 4.62 (dd, 2H, J = 5.0 Hz, J = 11.5 Hz). 13C{1H} NMR ($CDCl_3$, 125 MHz): δ 167.3, 166.7, 133.9, 133.4, 131.8, 129.7, 129.5, 128.7, 128.5, 127.0, 63.7, 50.9, 48.9. MS m/z (rel int.): 159 (100), 146 (78), 130 (14), 118 (36), 105 (37), 91 (39), 77 (37), 51 (10). HRMS: M(mo): 403.14197, HRMS (ESI-orbitrap) m/z: $[M + Na]^+$ Calcd for $C_{24}H_{21}NO_5Na$ 426.1312; Found 426.1302

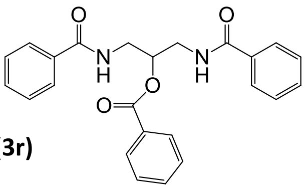

N,N'-[2-(benzoyloxy)-1,3-propanediyl]bis benzamide **(3r)**

White solid; R_f ($CHCl_3$/EtAc = 8:2) 0.8; Yield: 63 % (126.6 mg); Melting point: 143°C; 1H NMR ($CDCl_3$, 500 MHz): δ 8.05 (d, 2H, J = 7.3 Hz), 7.91 (d, 4H, J = 7.3 Hz), 7.59 (t, 1H, J = 7.3 Hz), 7.54 (t, 2H, J = 7.3 Hz), 7.49-7.42 (m, 8H), 5.32-5.29 (m, 1H), 3.97-3.92 (m, 2H), 3.81-3.76 (m, 2H). 13C{1H} NMR ($CDCl_3$, 125 MHz): δ 168.3, 166.1, 133.9, 133.4, 131.8, 129.8, 129.7, 128.7, 128.5, 127.2, 72.1, 39.6, 35.4. MS m/z (rel int.): 280 (4), 262 (13), 175 (90), 146

(100), 134 (54), 105 (89), 91 (43), 77 (59), 51 (13). HRMS (ESI-orbitrap) m/z: $[M + H]^+$ Calcd for C24H23N2O4 403.1652; Found 403.1649

[1] Movassaghi, M.; Schmidt, M. A. *Org. Lett.* **2005***, 7, 12*, 2453–2456. https://doi.org/10.1021/ol050773y
[2] Nechab, M.; Kumar, D. N.; Philouze, C.; Einhorn, C.; Einhorn, *J. Angew. Chem. Int. Ed.* **2007**, *46, 17*, 3080-3083. https://doi.org/10.1002/anie.200603780
[3] Mahmoud, S.; Muhammad, A. *Arch. Pharm. Chem. Life Sci.* **2010,** *10*, 639-647. 10.1002/ardp.201000105
[4] Seki, K.; Yamashita, T.; Isegawa, J.; Fukuda, M.; Shimamura, H.; Ohki, M. *Chem. Pharm. Bull.* **1983**, *31*, 4116-4126. https://doi.org/10.1248/cpb.31.4116
[5] Caldwell, N.; Jamieson, C.; Simpson, I.; Watson, A. J. B. *ACS Sustainable Chem. Eng.* **2013**, *1, 10*, 1339-1344. https://doi.org/10.1021/sc400204g
[6] Hirvelae, L. (2005). U.S. Patent *No. 20050176742 A1.*
[7]Morcuende, A.; Ors, M.; Valverde, S.;Herradón, B. J. Org. Chem. 1996, 61, 5264-5270. https://doi.org/10.1021/jo9605511
[8] Nishii, Y.; Hirai, T.; Fernanez, S.; Knochel, P.; Mashima, K. *Eur. J. Org. Chem.* **2017**, *34, 15*, 5010-5014. https://doi.org/10.1002/ejoc.201700748
[9] Boto, A.; Hernández, D.; Hernández, R.; Montoya, A.; Suárez, E. *Eur. J. Org. Chem.* **2007**, *2*, 325-334. https://doi.org/10.1002/ejoc.200600720

[10] Mamaghami, M.; Mahmoodi, N. O.; Ghasemi, S. F. J. *Iran. Chem. Soc*. **2010**, *7*, 972-977. https://doi.org/10.1007/bf03246093
[11] Kollár, L.; Erdélyi, Á.; Rasheed, H.; Takács, A. Molecules, 2021, 26, 1813. 10.3390/molecules26061813
[12] Arisawa, M.; Yamada, T.; Yamaguchi, M. *Tetrahedron Lett*. **2010**, *51, 47*, 6090-6092. https://doi.org/10.1016/j.tetlet.2010.09.009

8 ^{1}H, $^{13}C\{^{1}H\}$ and MS spectrum of isolated compounds

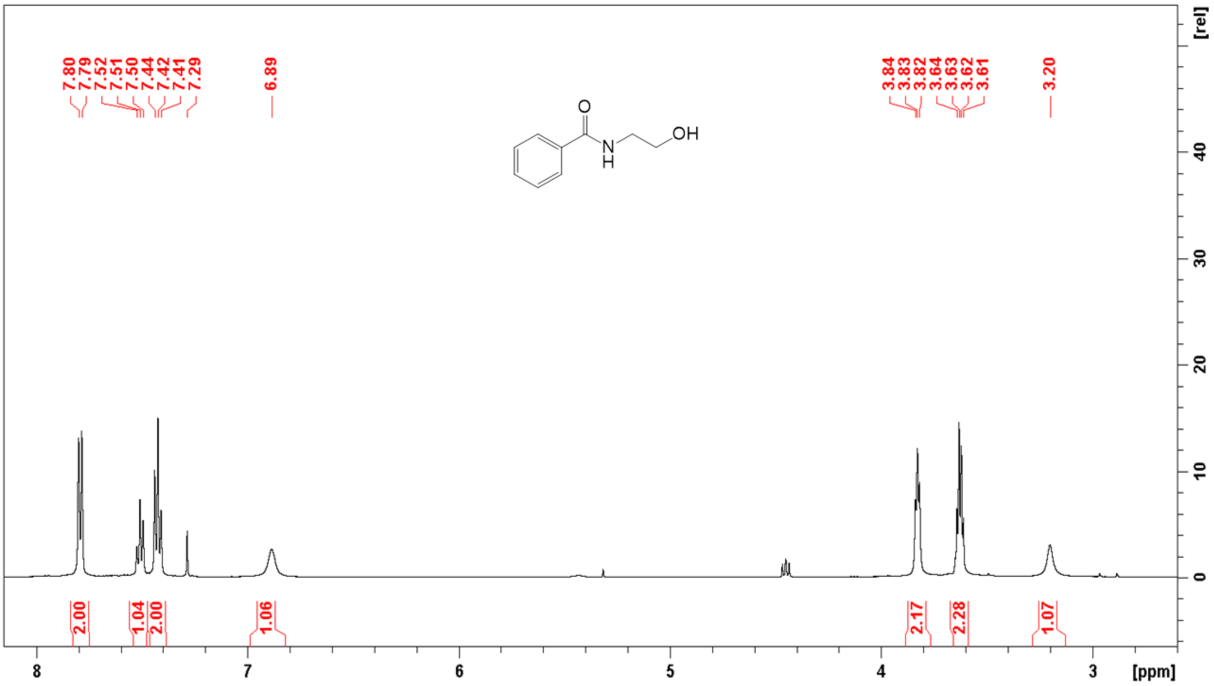

Figure S1. ^{1}H NMR spectrum of **3a** (500 MHz, $CDCl_3$)

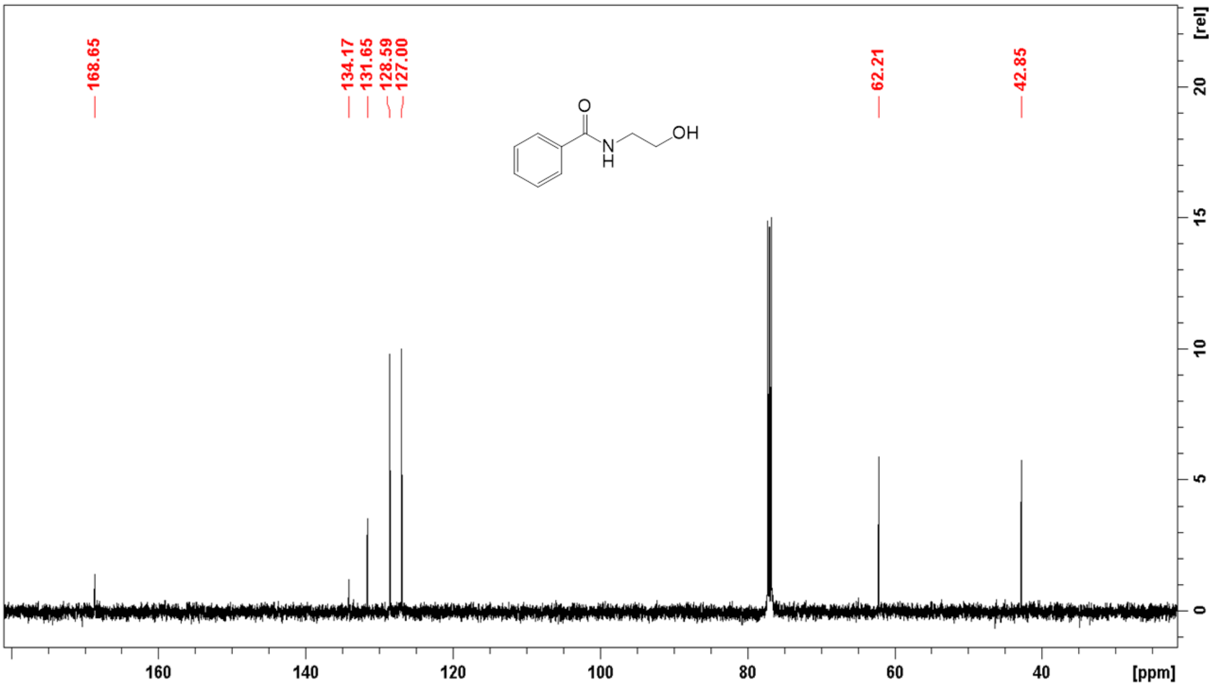

Figure S2. $^{13}C\{^{1}H\}$ NMR spectrum of **3a** (125 MHz, $CDCl_3$)

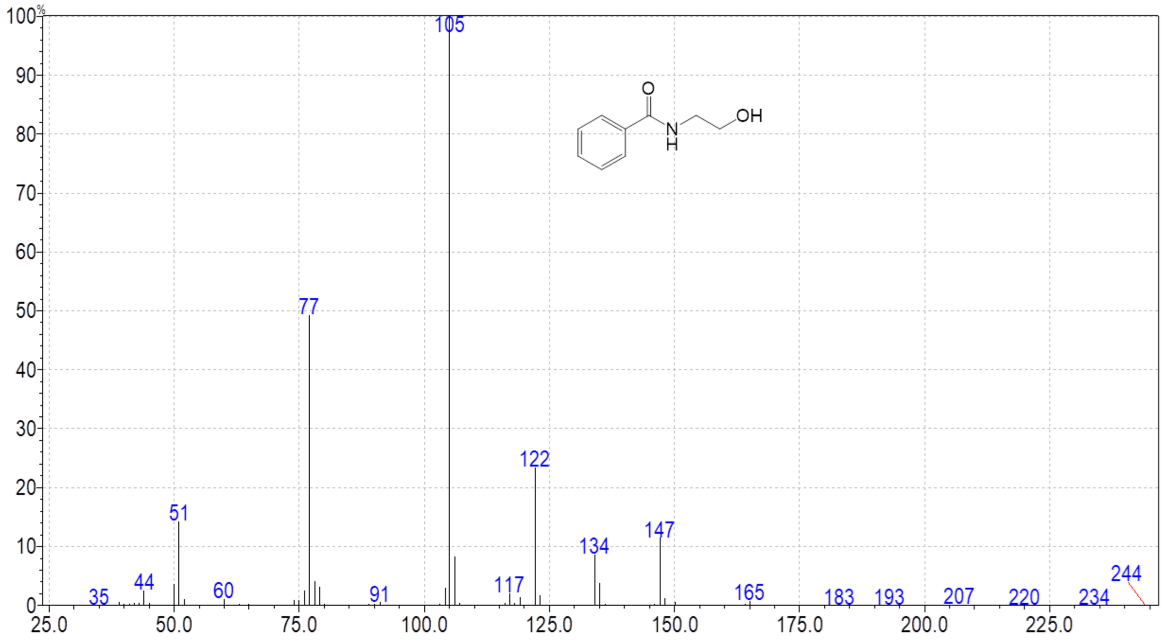

Figure S3. MS spcetrum of **3a** (m/z)

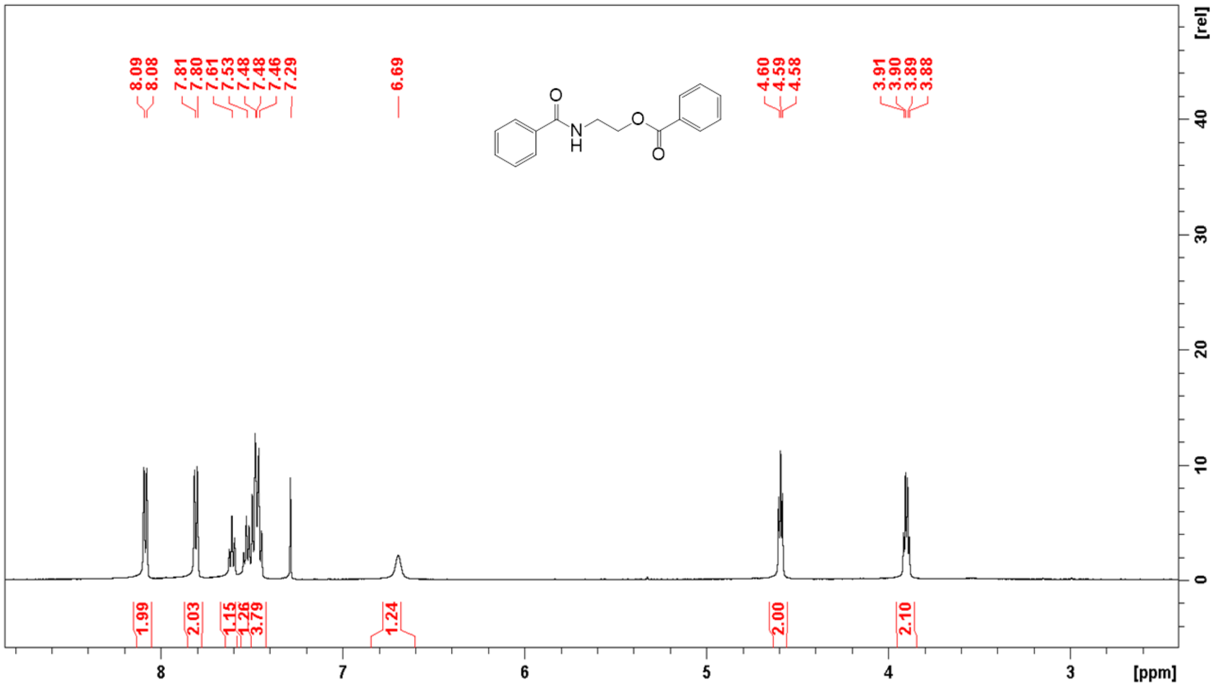

Figure S4. ^{1}H NMR spectrum of 4**a** (500 MHz, $CDCl_3$)

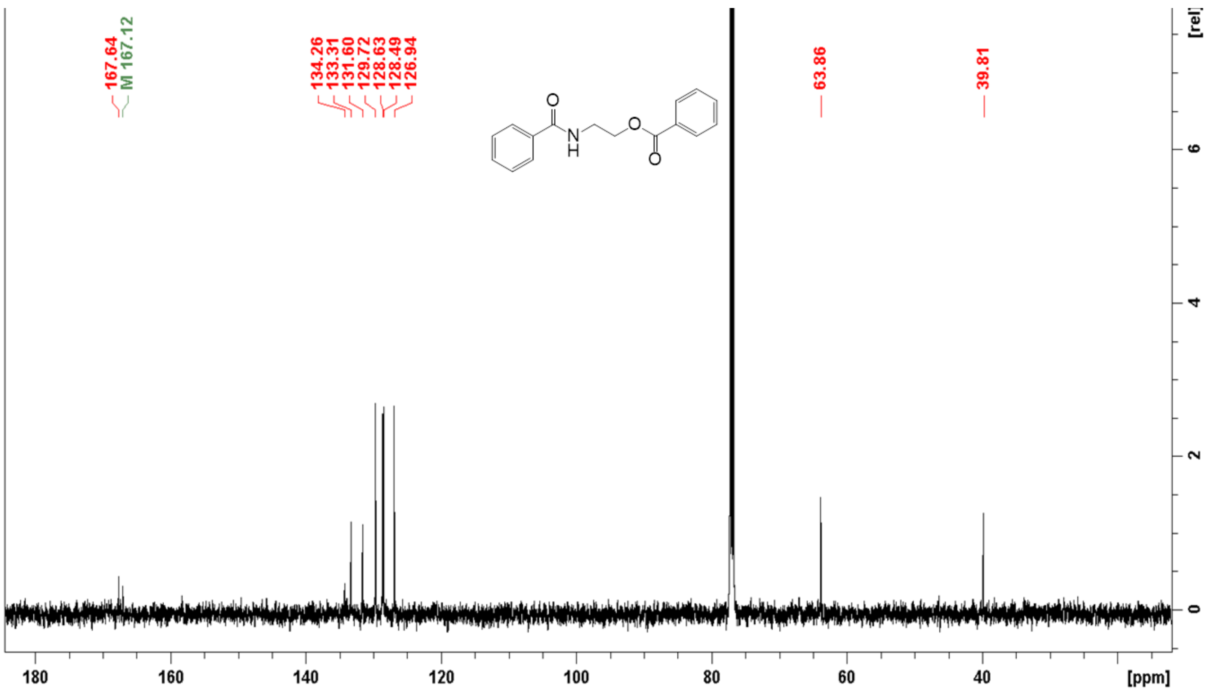

Figure S5. $^{13}C\{^1H\}$ NMR spectrum of **4a** (125 MHz, $CDCl_3$)

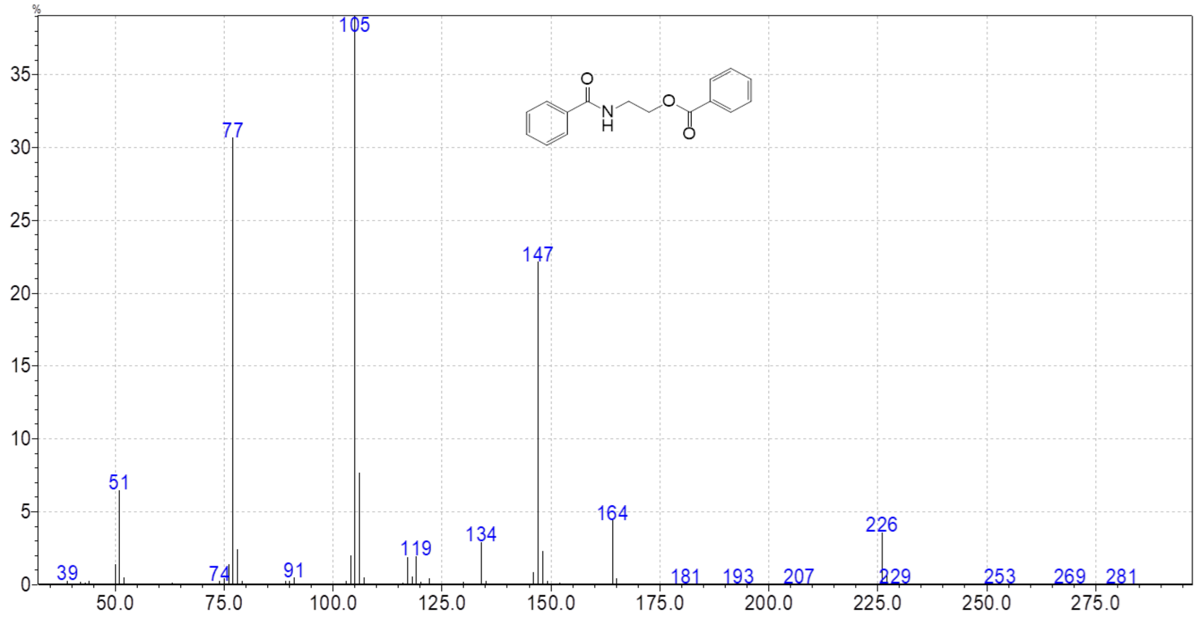

Figure S6. MS spcetrum of **4a** (m/z)

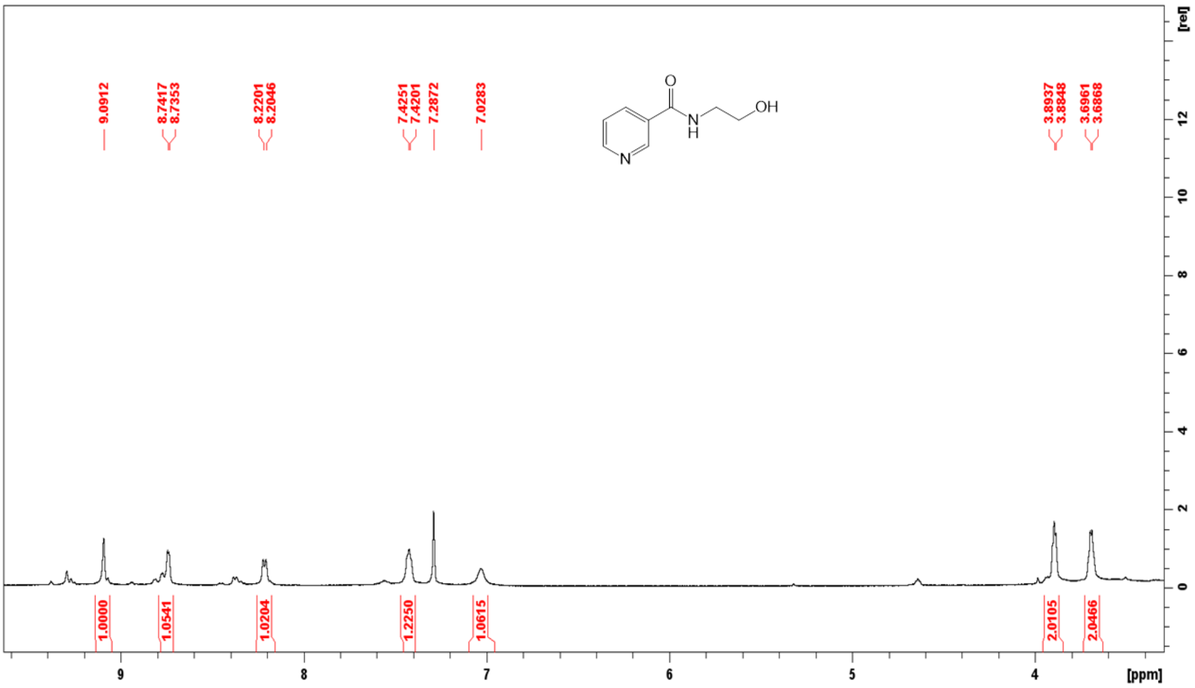

Figure S7. ^{1}H NMR spectrum of **3g** (500 MHz, $CDCl_3$)

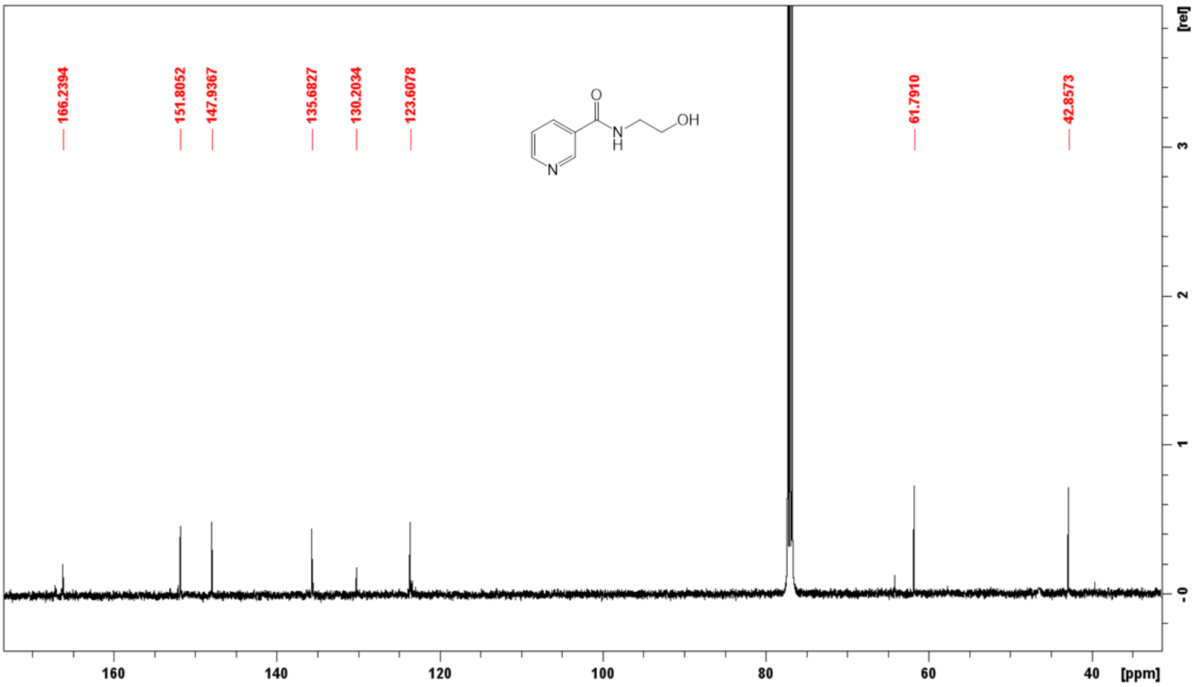

Figure S8. $^{13}C\{^{1}H\}$ NMR spectrum of **3g** (125 MHz, $CDCl_3$)

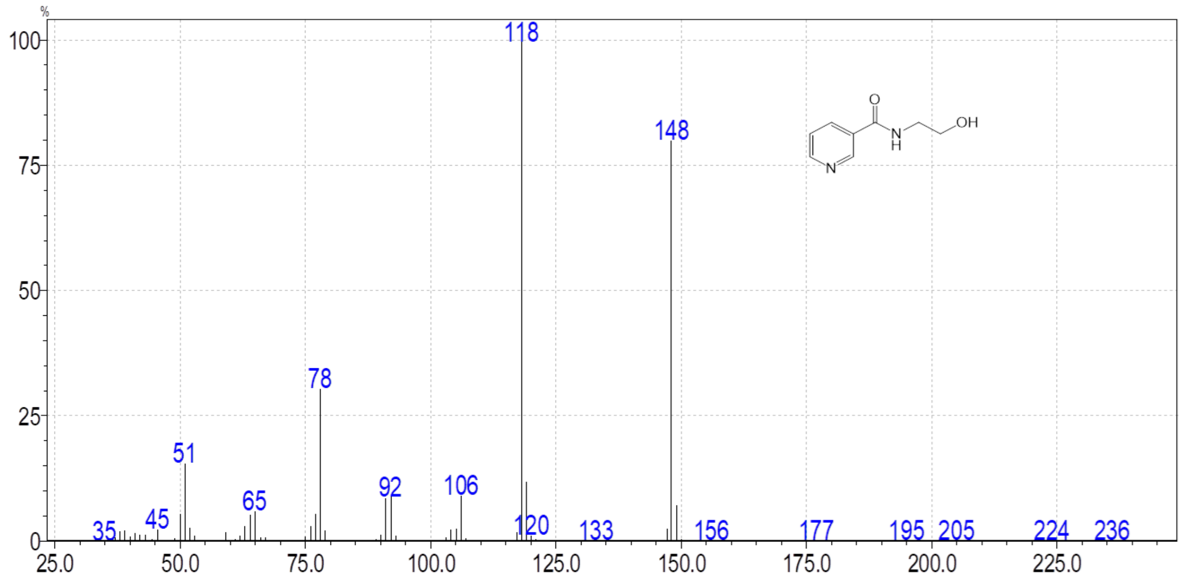

Figure S9. MS spcetrum of **3g** (m/z)

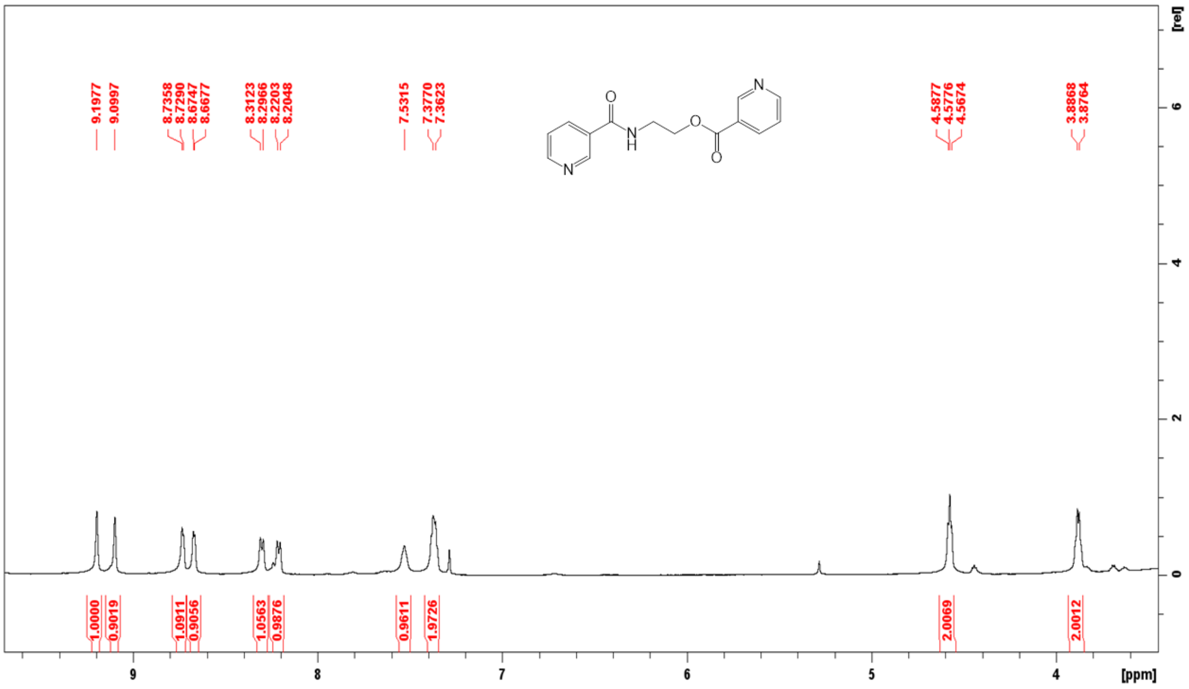

Figure S10. ^{1}H NMR spectrum of **4g** (500 MHz, $CDCl_3$)

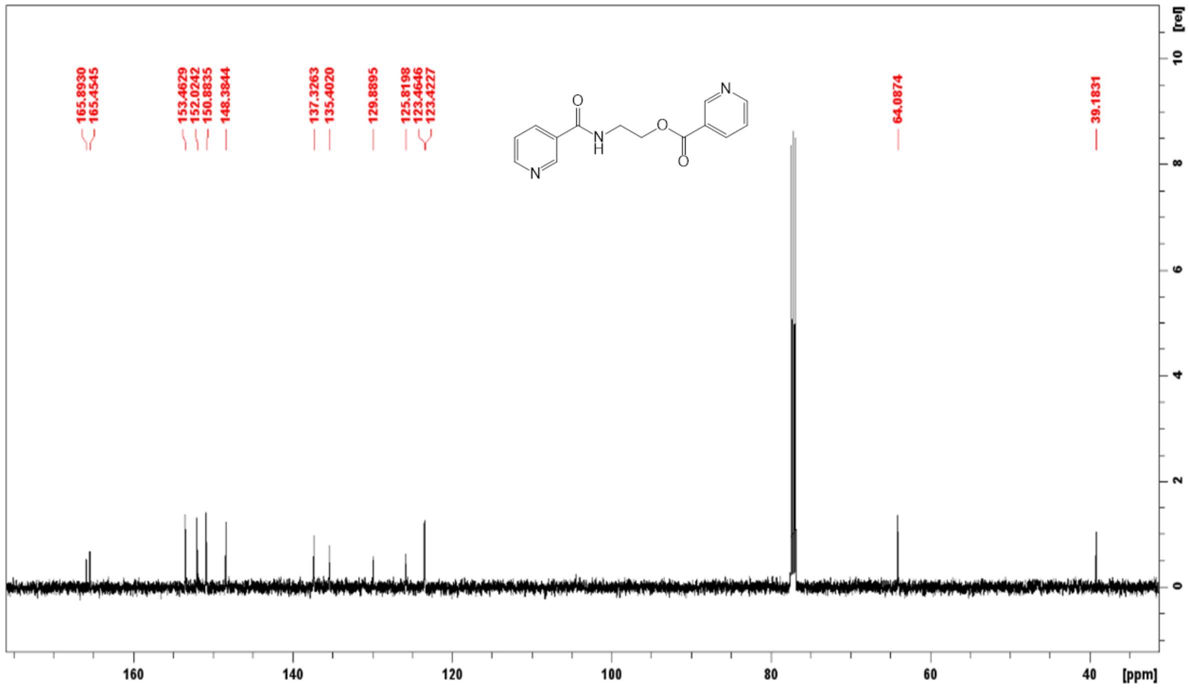

Figure S11. $^{13}C\{^{1}H\}$ NMR spectrum of **4g** (125 MHz, $CDCl_3$)

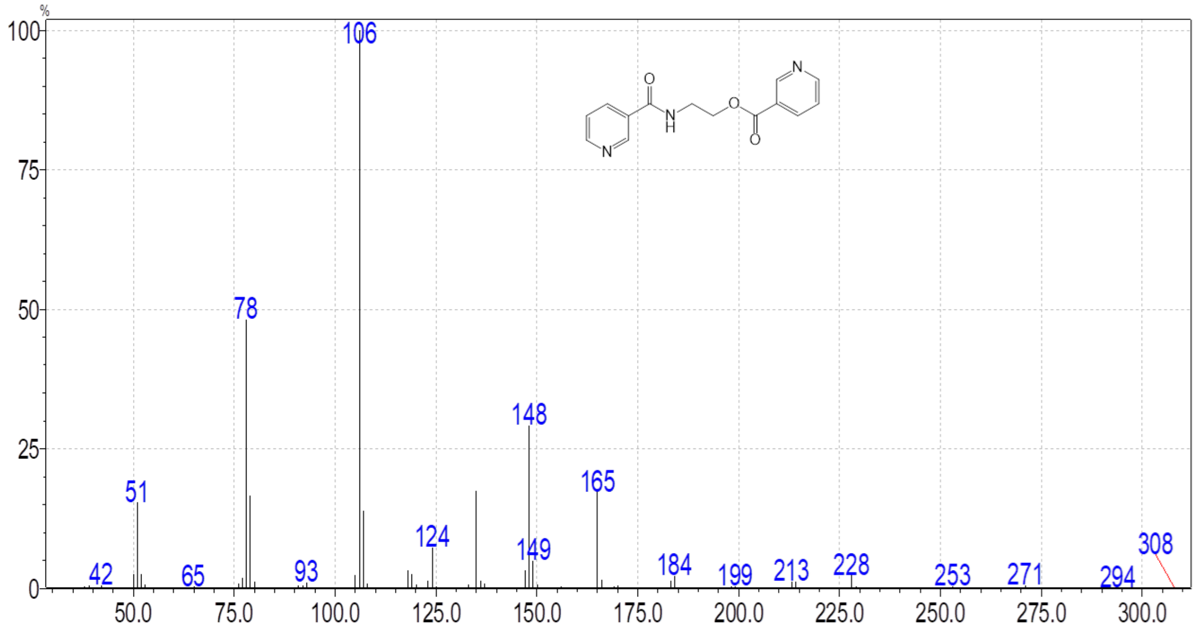

Figure S12. MS spcetrum of **4g** (m/z)

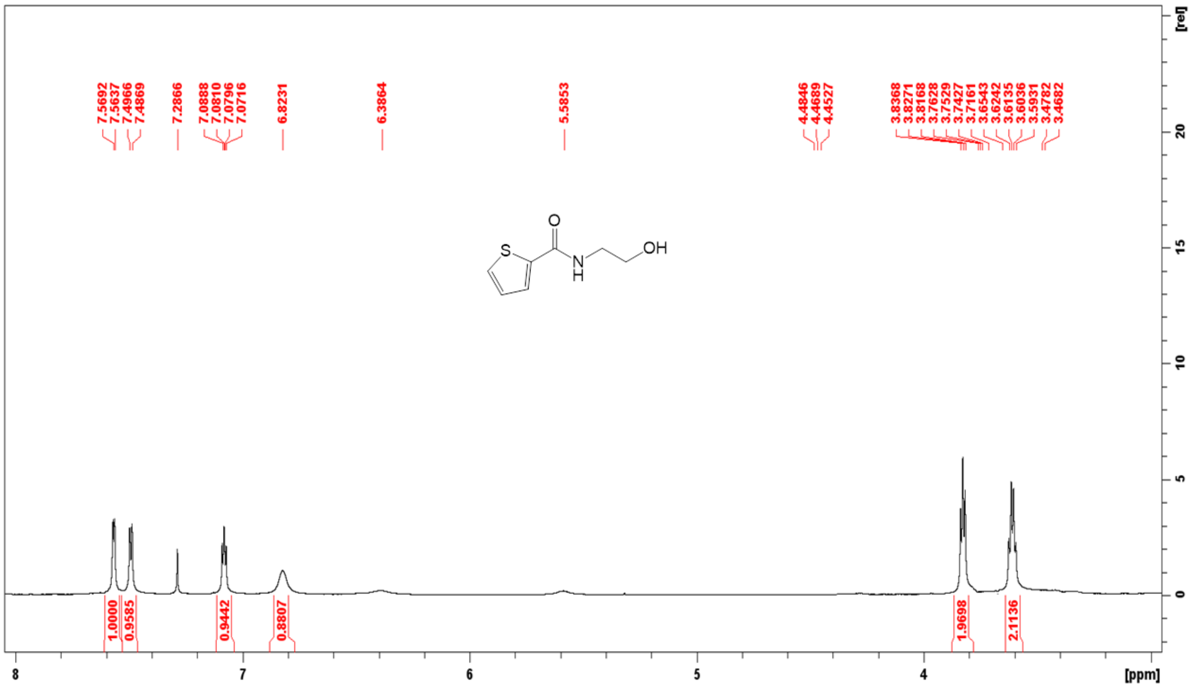

Figure S13. ^{1}H NMR spectrum of **3h** (500 MHz, $CDCl_3$)

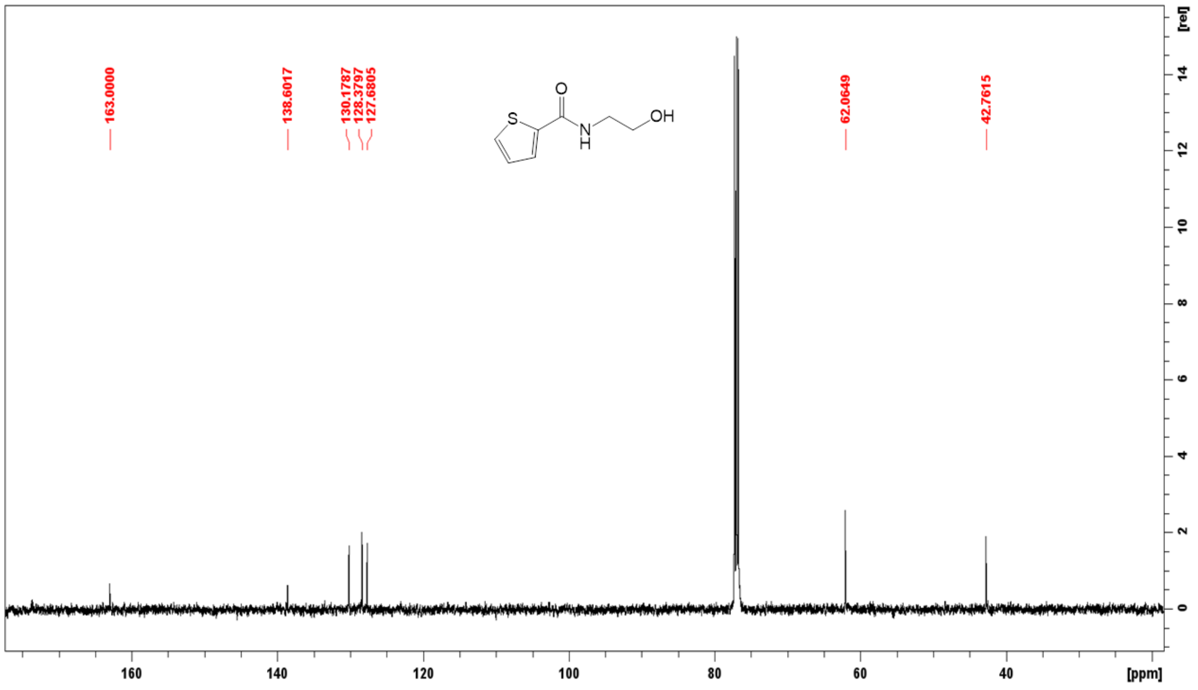

Figure S14. $^{13}C\{^{1}H\}$ NMR spectrum of **3h** (125 MHz, $CDCl_3$)

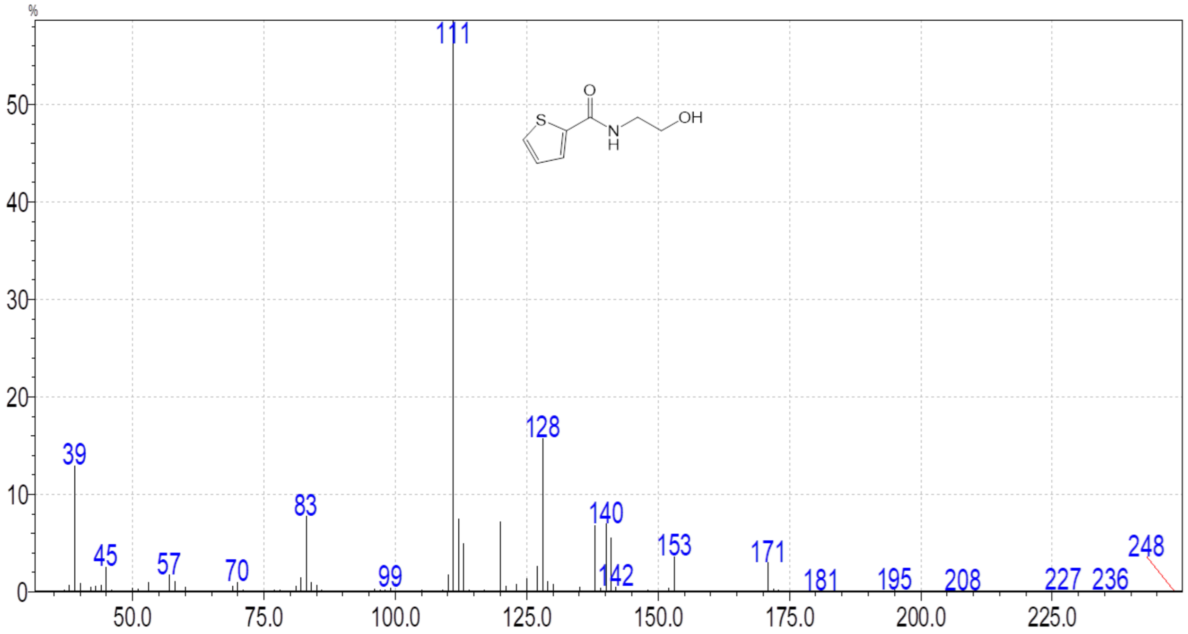

Figure S15. MS spcetrum of **3h** (m/z)

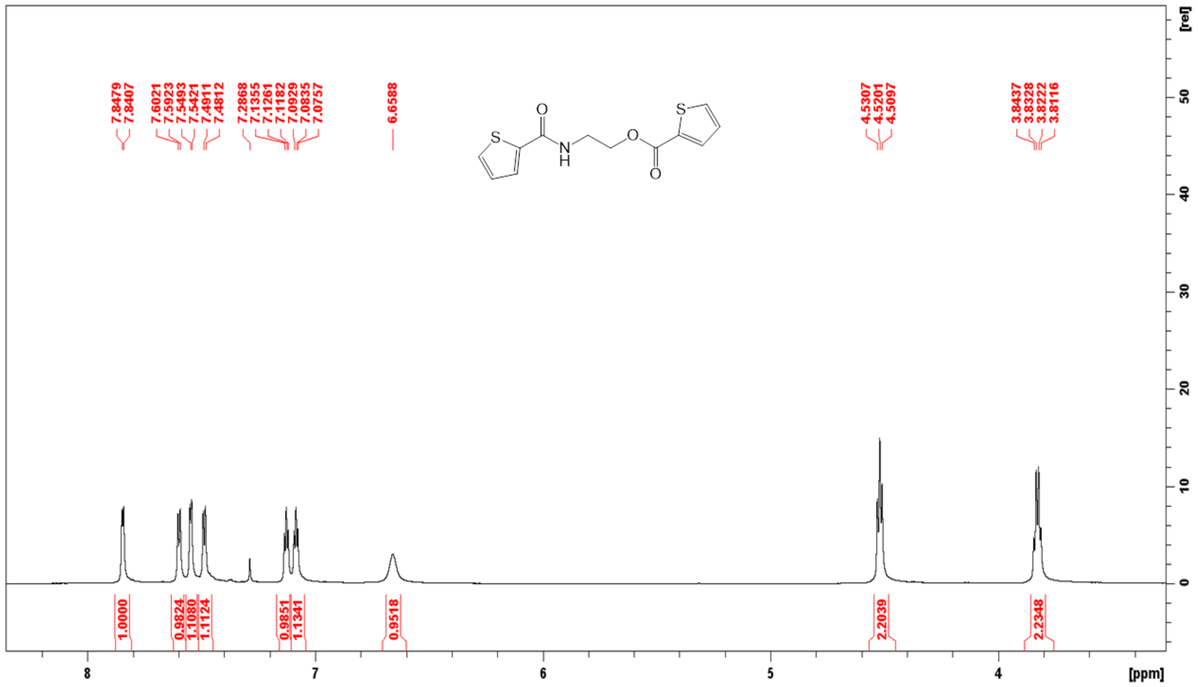

Figure S16. ^{1}H NMR spectrum of **4h** (500 MHz, $CDCl_3$)

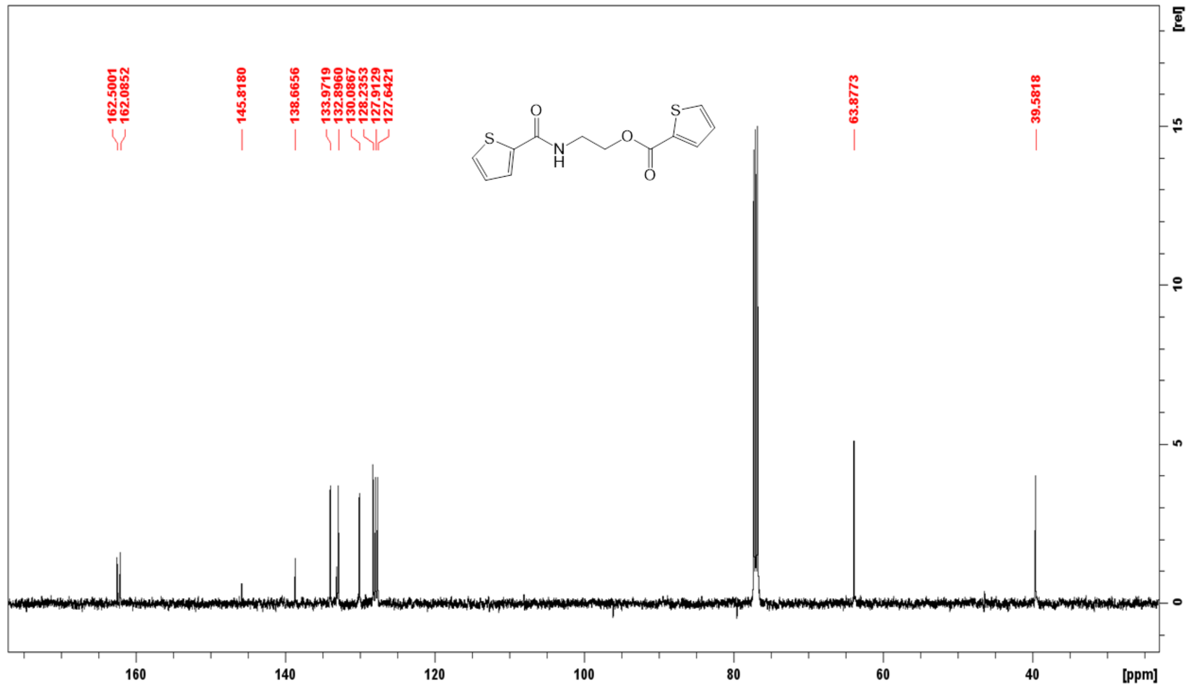

Figure S17. $^{13}C\{^{1}H\}$ NMR spectrum of **4h** (125 MHz, $CDCl_3$)

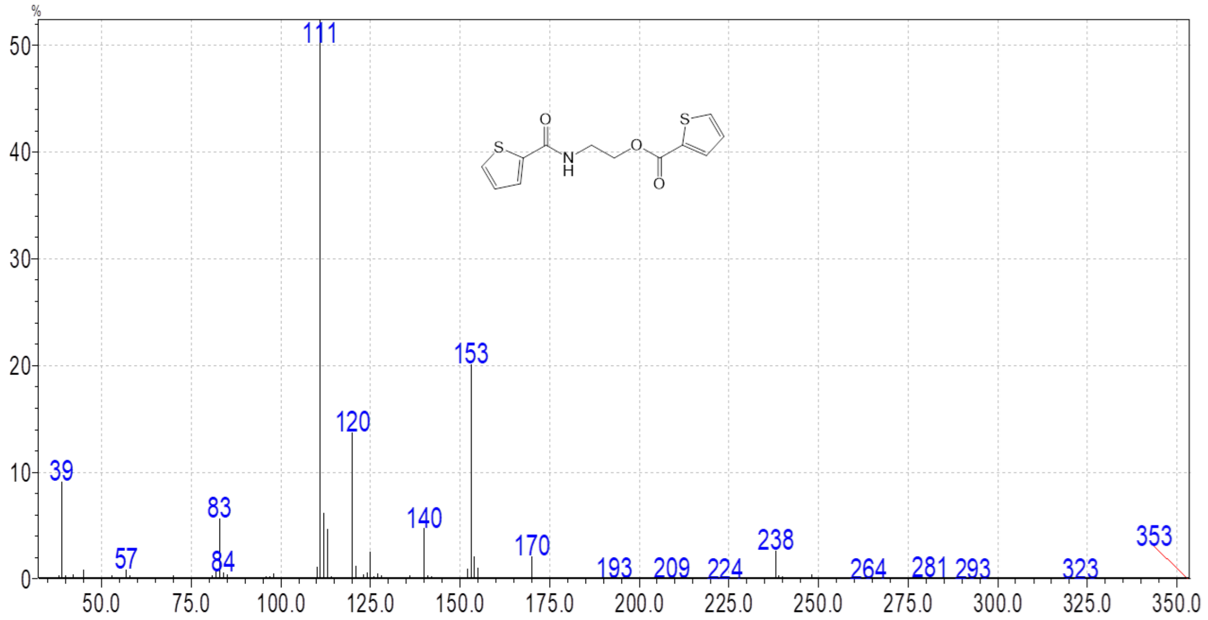

Figure S18. MS spcetrum of **4h** (m/z)

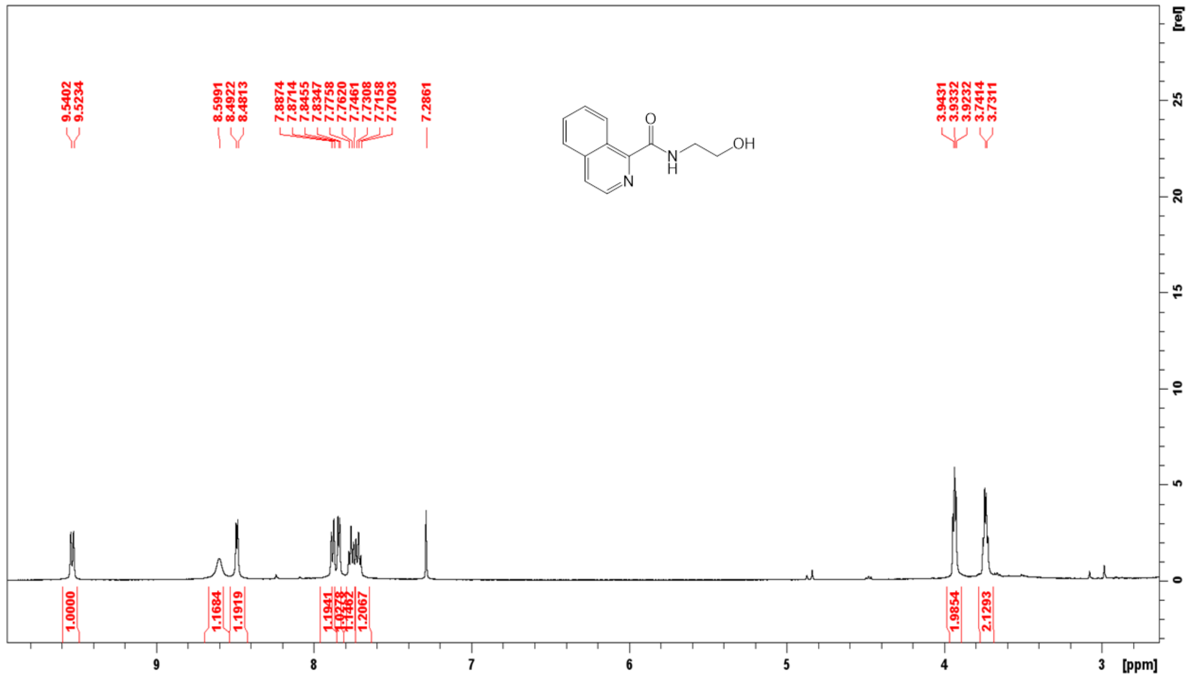

Figure S19. ^{1}H NMR spectrum of **3i** (500 MHz, $CDCl_3$)

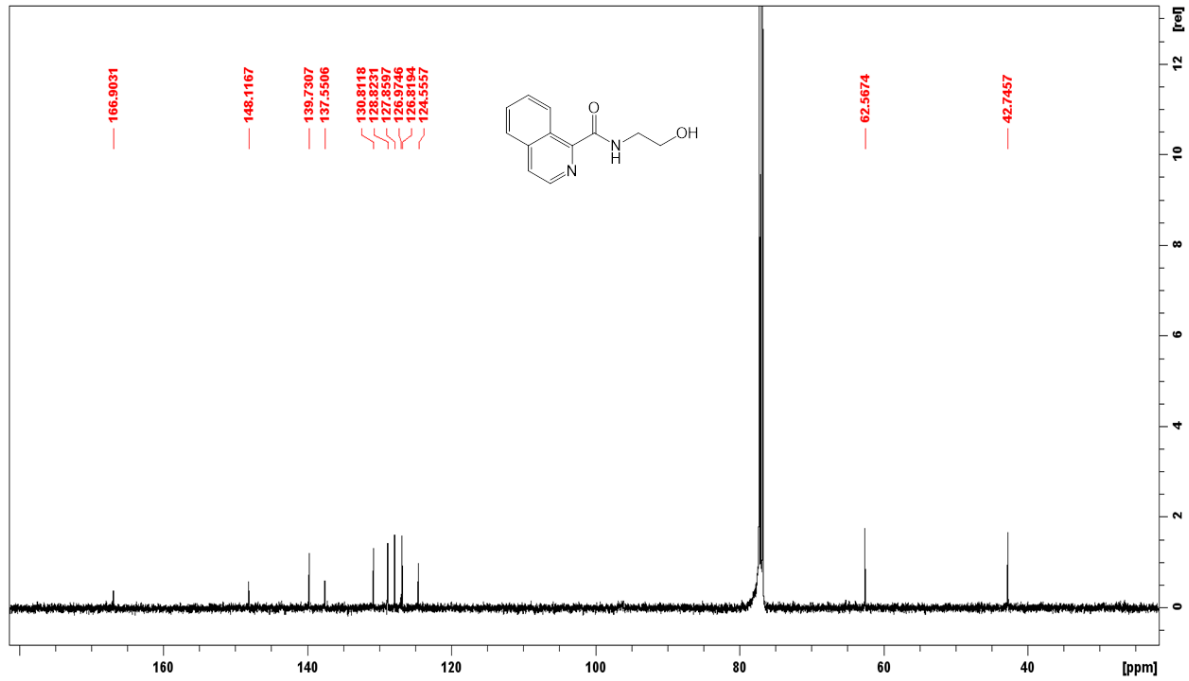

Figure S20. ^{13}C{^{1}H} NMR spectrum of **3i** (125 MHz, $CDCl_3$)

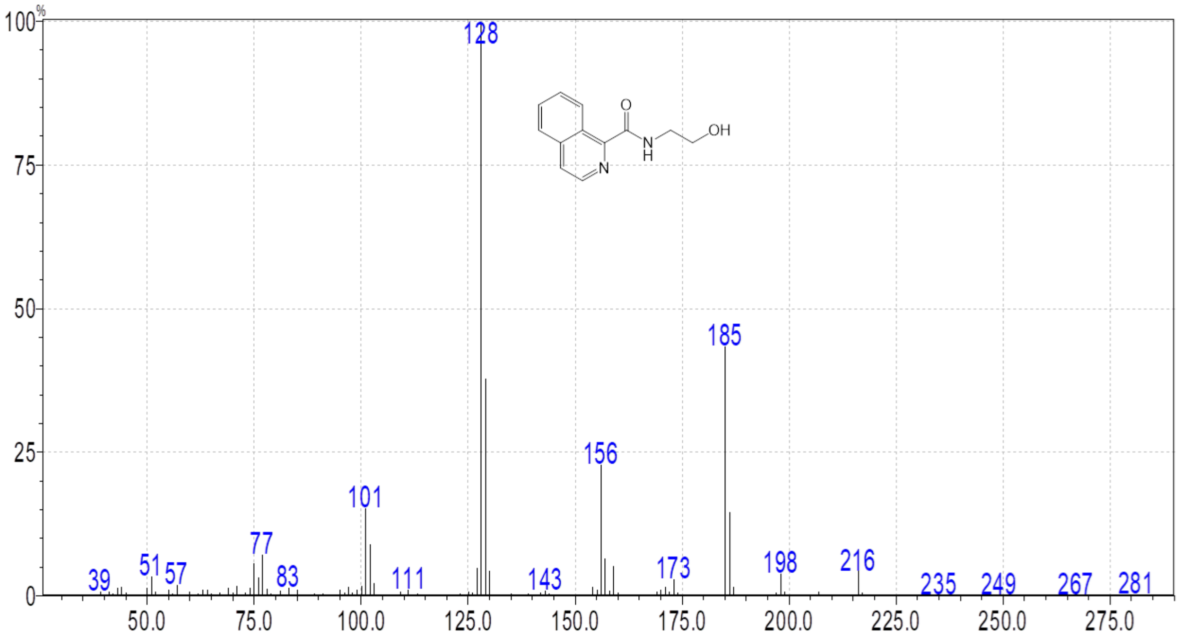

Figure S21. MS spcetrum of **3i** (m/z)

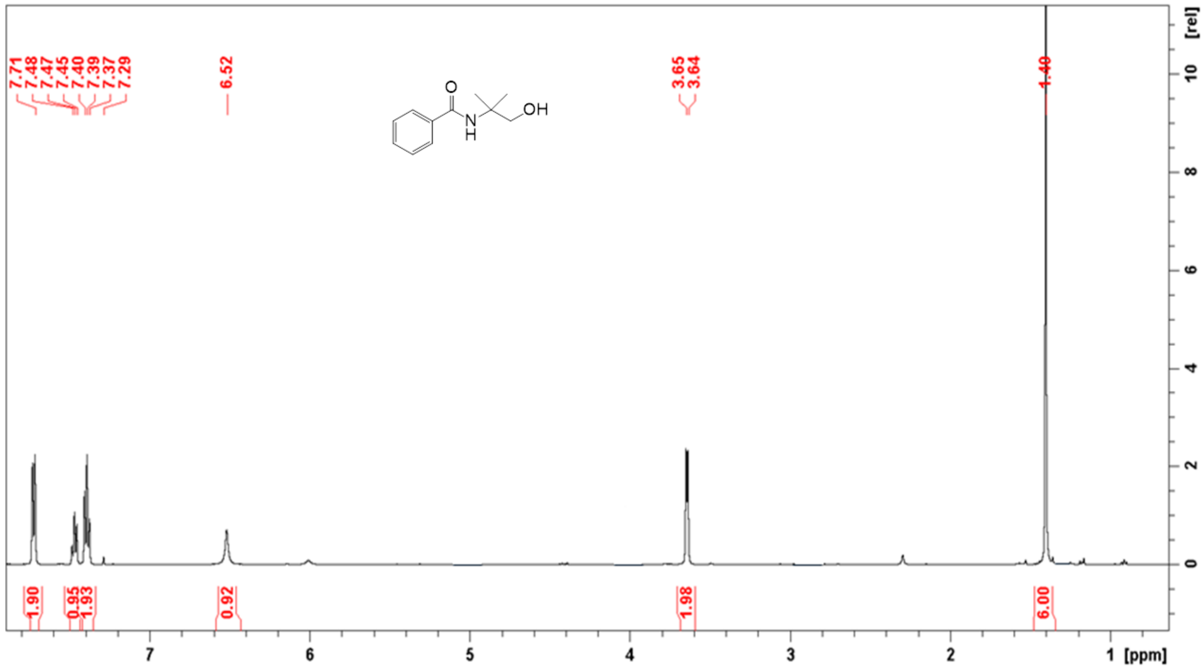

Figure S22. ^{1}H NMR spectrum of **3j** (500 MHz, $CDCl_3$)

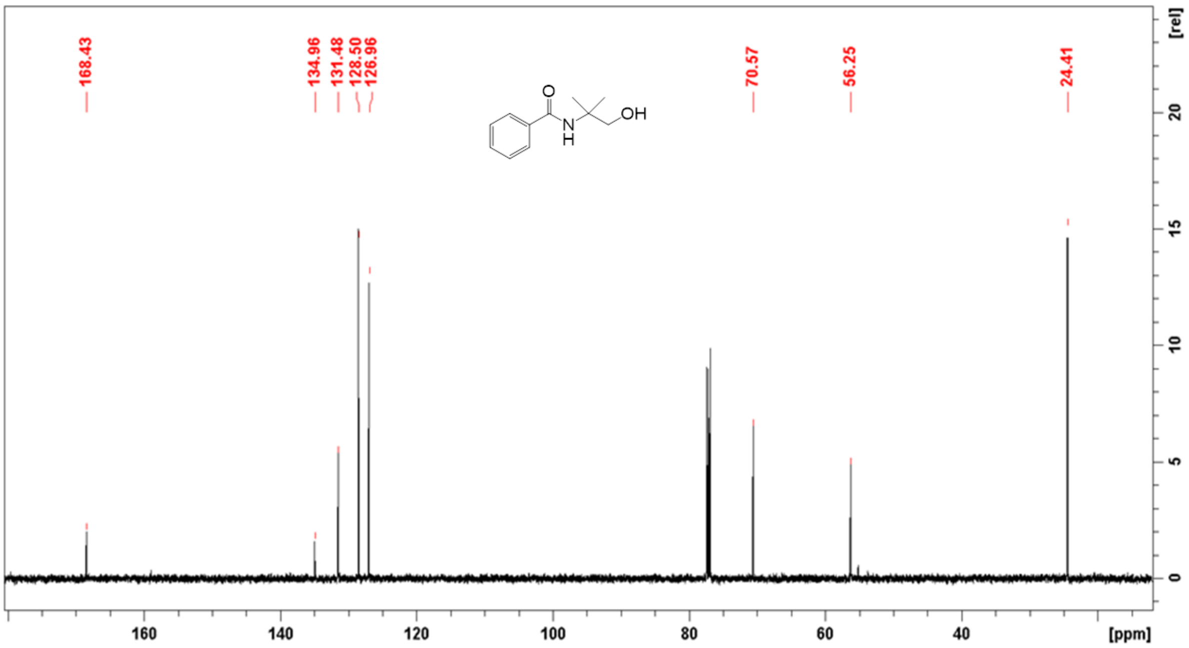

Figure S23. $^{13}C\{^{1}H\}$ NMR spectrum of **3j** (125 MHz, $CDCl_3$)

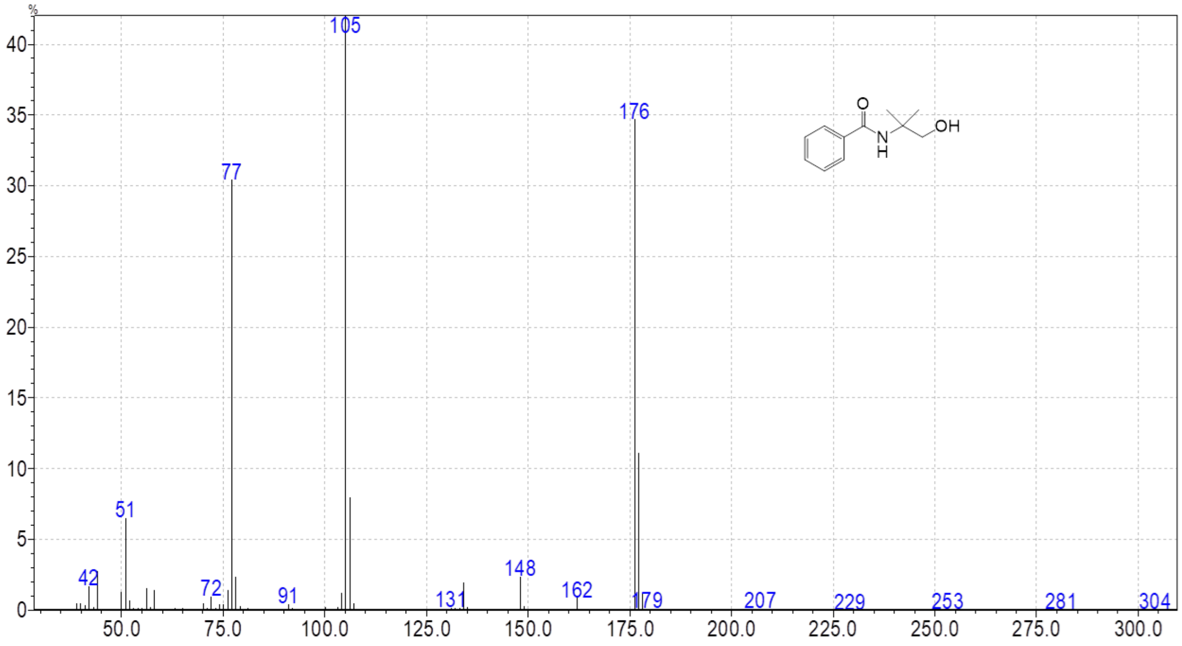

Figure S24. MS spcetrum of **3j** (m/z)

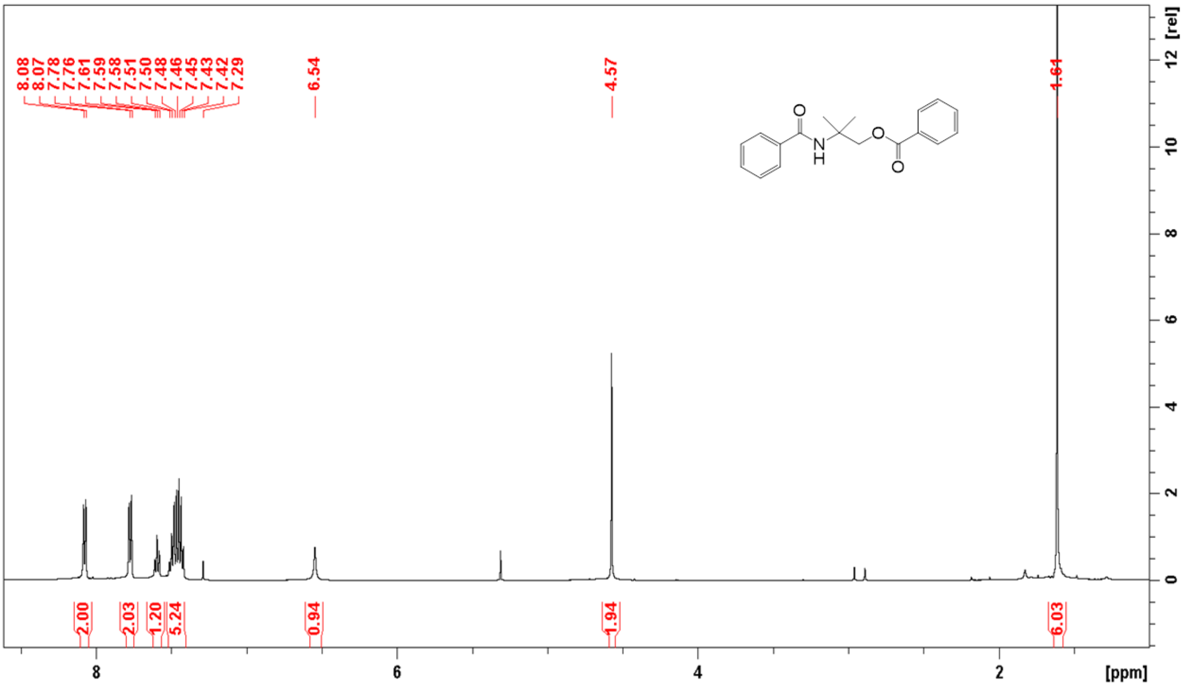

Figure S25. ^{1}H NMR spectrum of **4j** (500 MHz, $CDCl_3$)

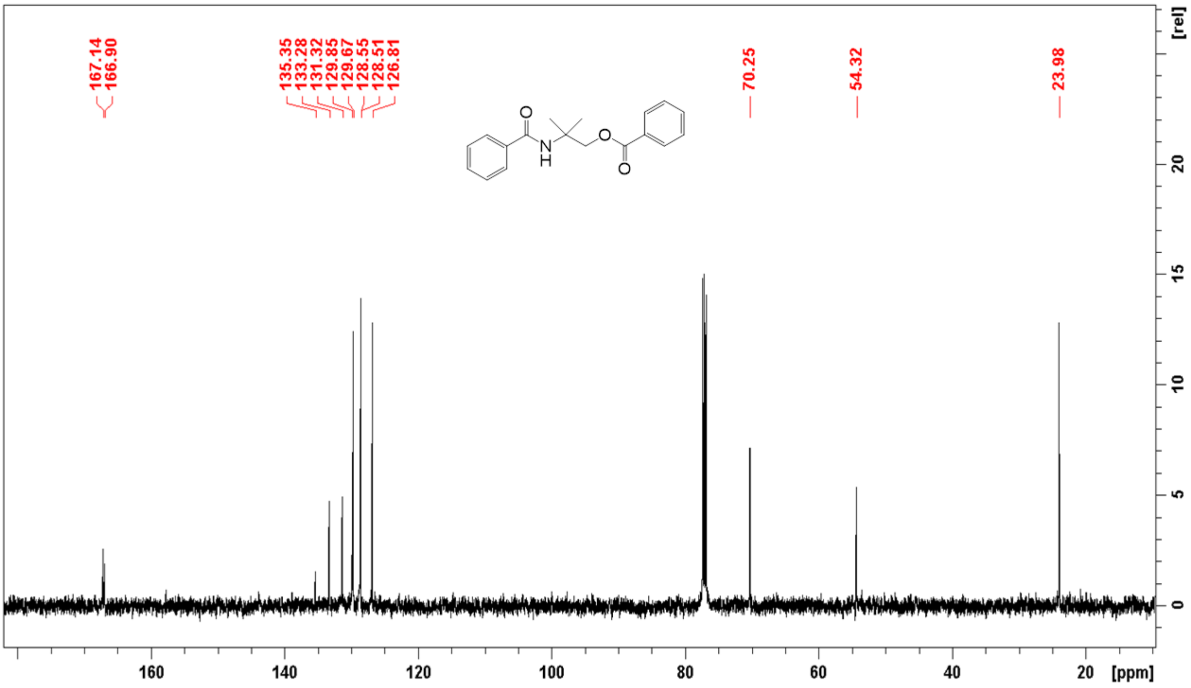

Figure S26. $^{13}C\{^{1}H\}$ NMR spectrum of **4j** (125 MHz, $CDCl_3$)

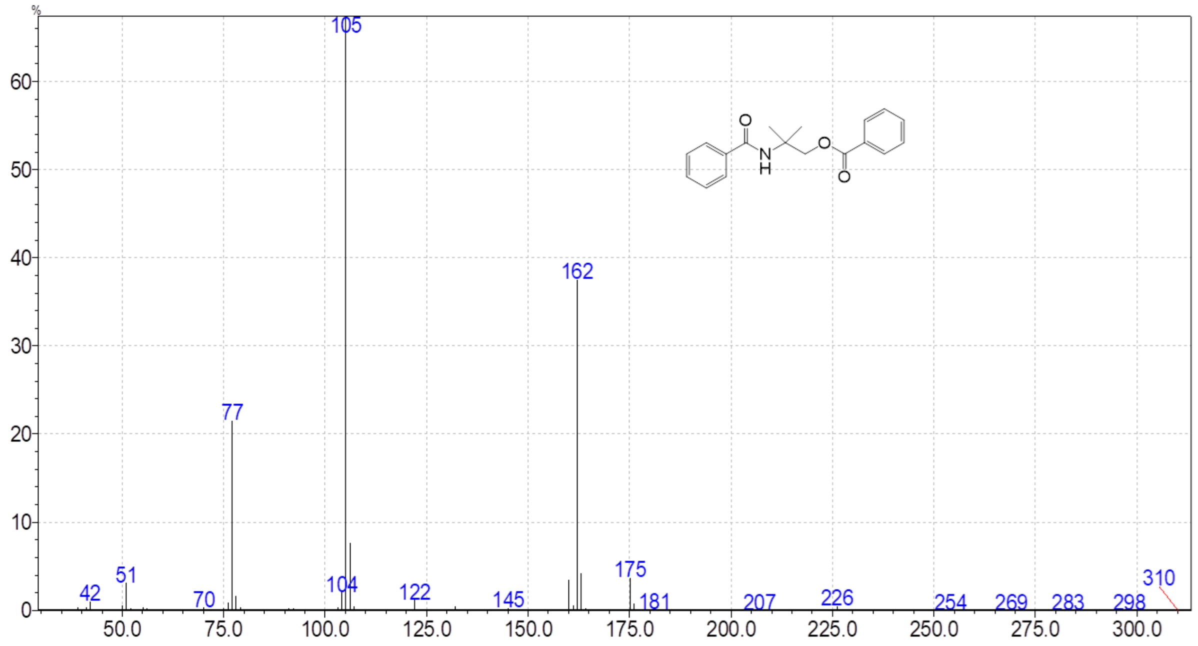

Figure S27. MS spcetrum of **4j** (m/z)

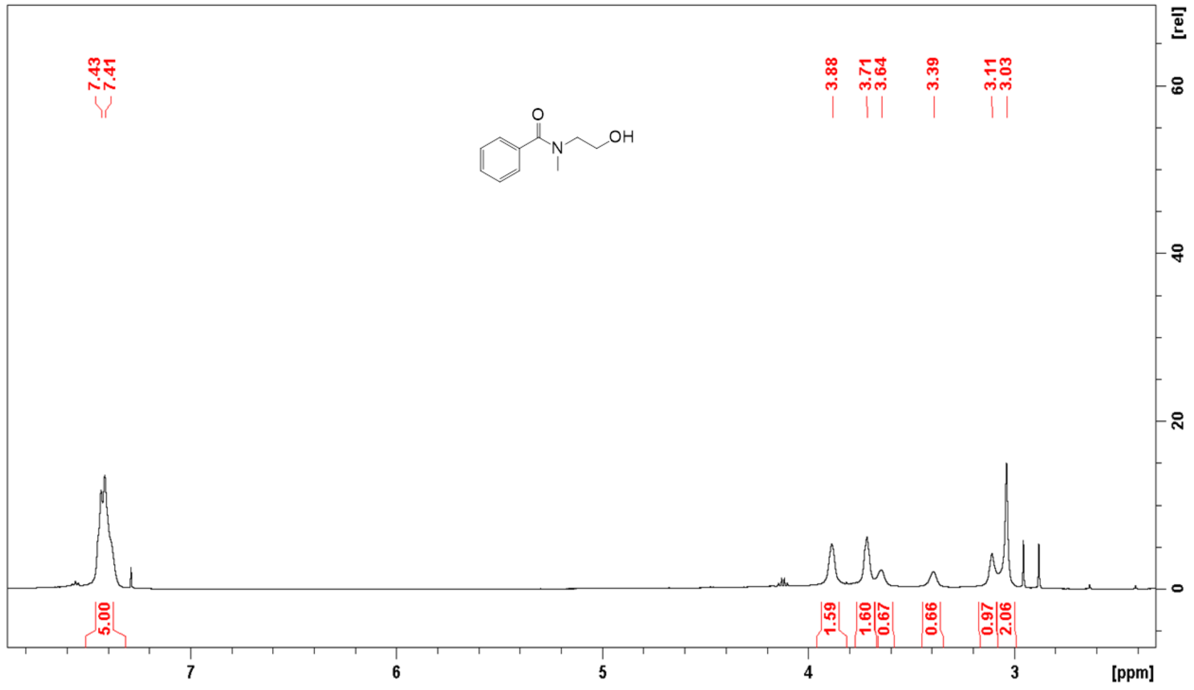

Figure S28. ^{1}H NMR spectrum of **3k** (500 MHz, $CDCl_3$)

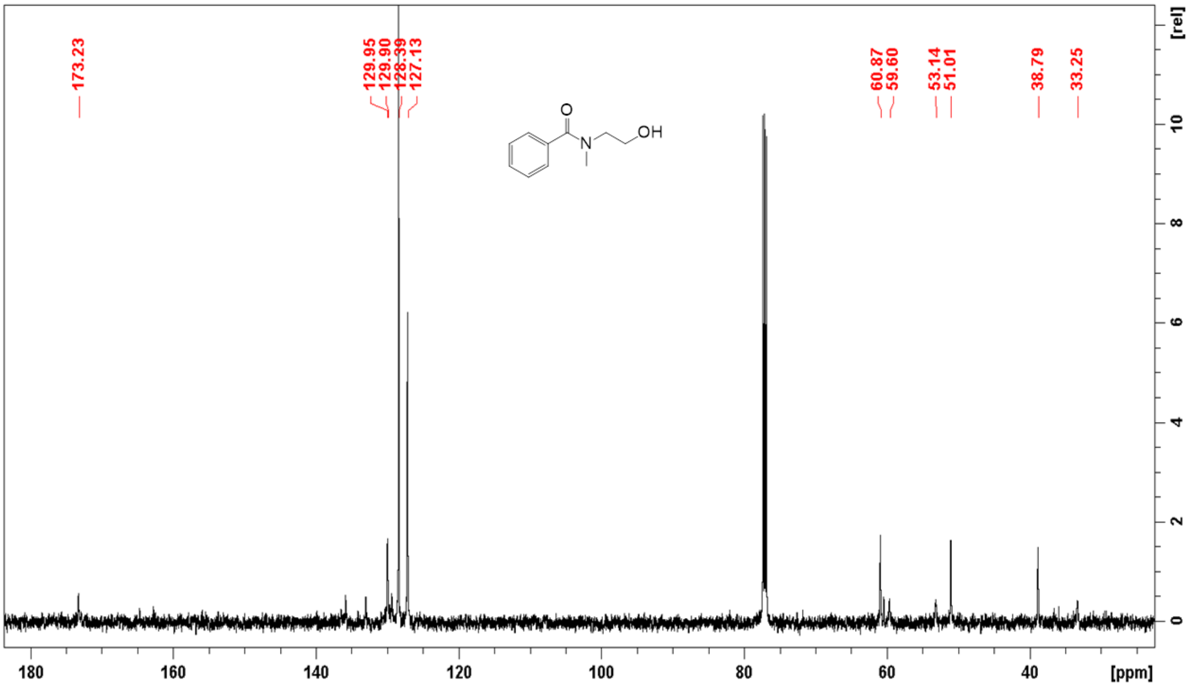

Figure S29. $^{13}C\{^1H\}$ NMR spectrum of **3k** (125 MHz, $CDCl_3$)

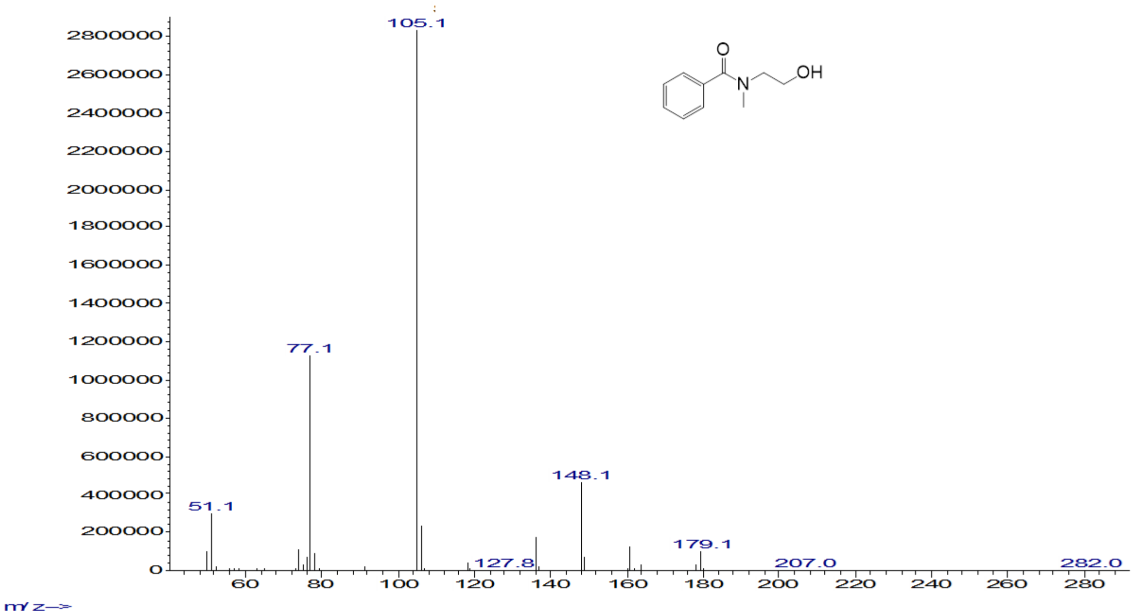

Figure S30. MS spcetrum of **3k** (m/z)

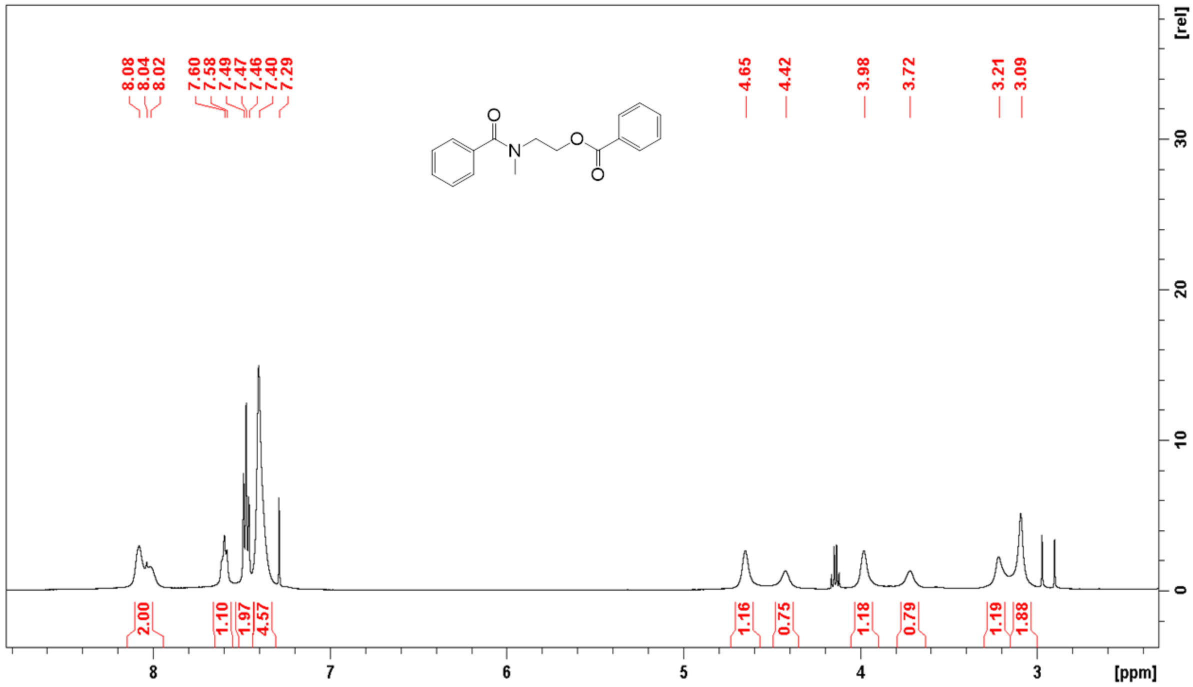

Figure S31. ^{1}H NMR spectrum of **4k** (500 MHz, $CDCl_3$)

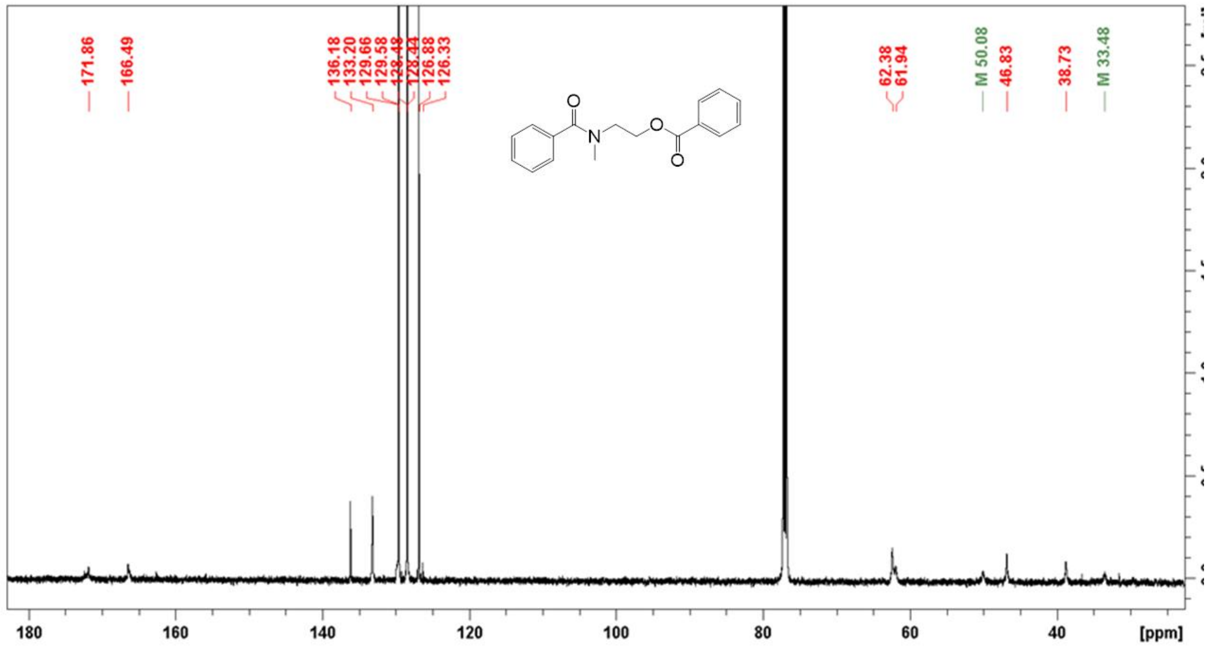

Figure S32. $^{13}C\{^{1}H\}$ NMR spectrum of **4k** (125 MHz, $CDCl_3$)

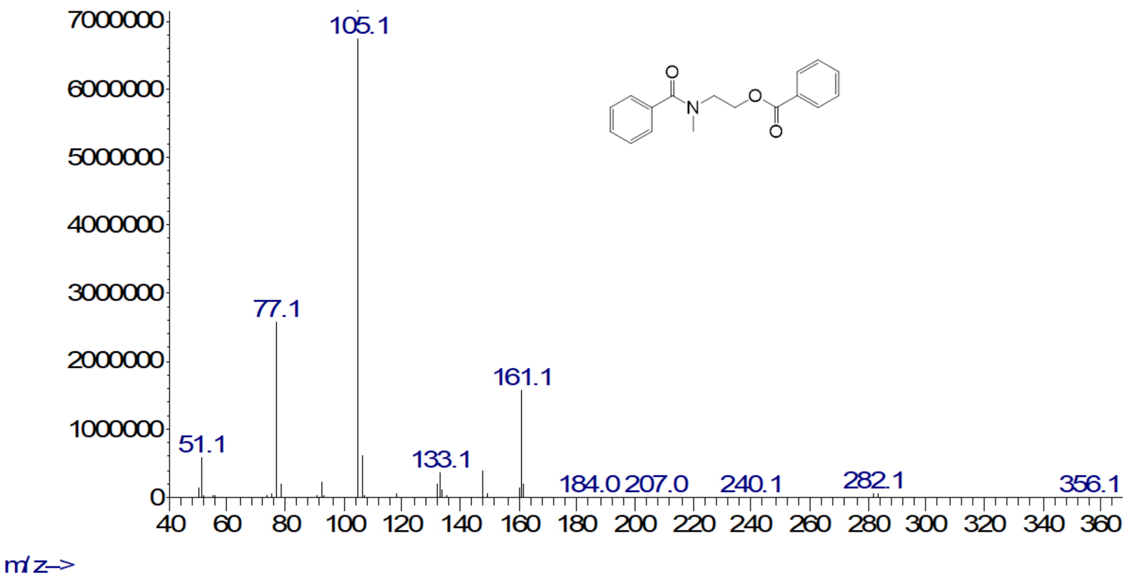

Figure S33. MS spcetrum of **4k** (m/z)

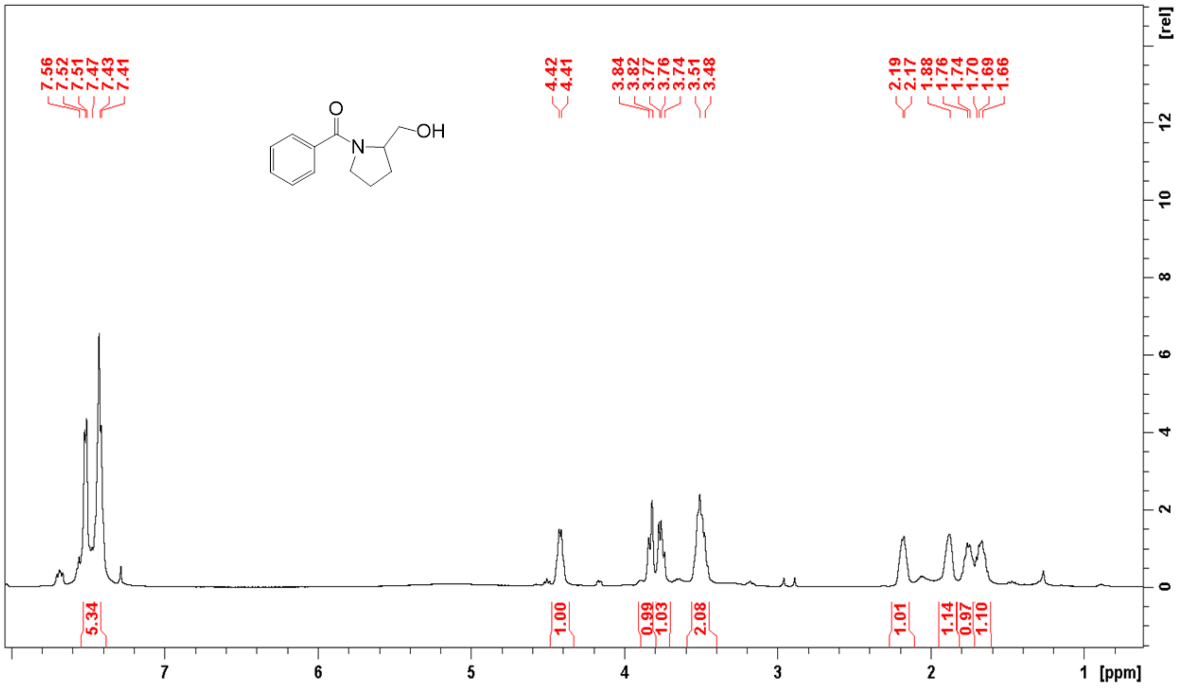

Figure S34. ^{1}H NMR spectrum of **3l** (500 MHz, $CDCl_3$)

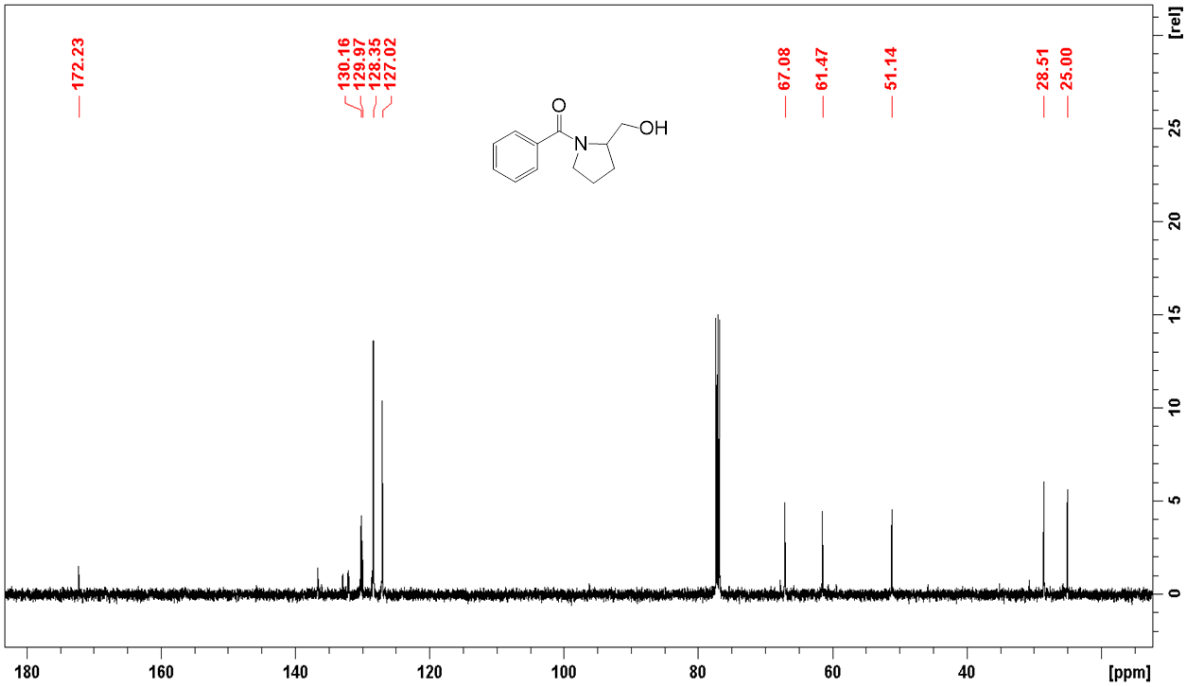

Figure S35. $^{13}C\{^1H\}$ NMR spectrum of **3l** (125 MHz, $CDCl_3$)

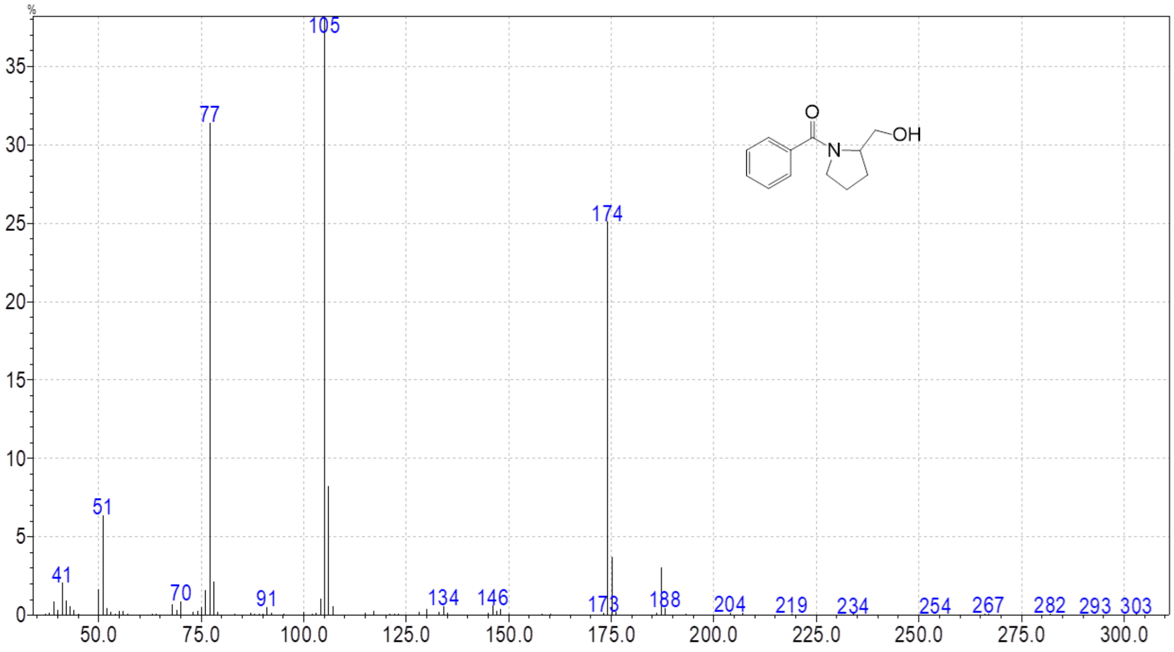

Figure S36. MS spcetrum of **3l** (m/z)

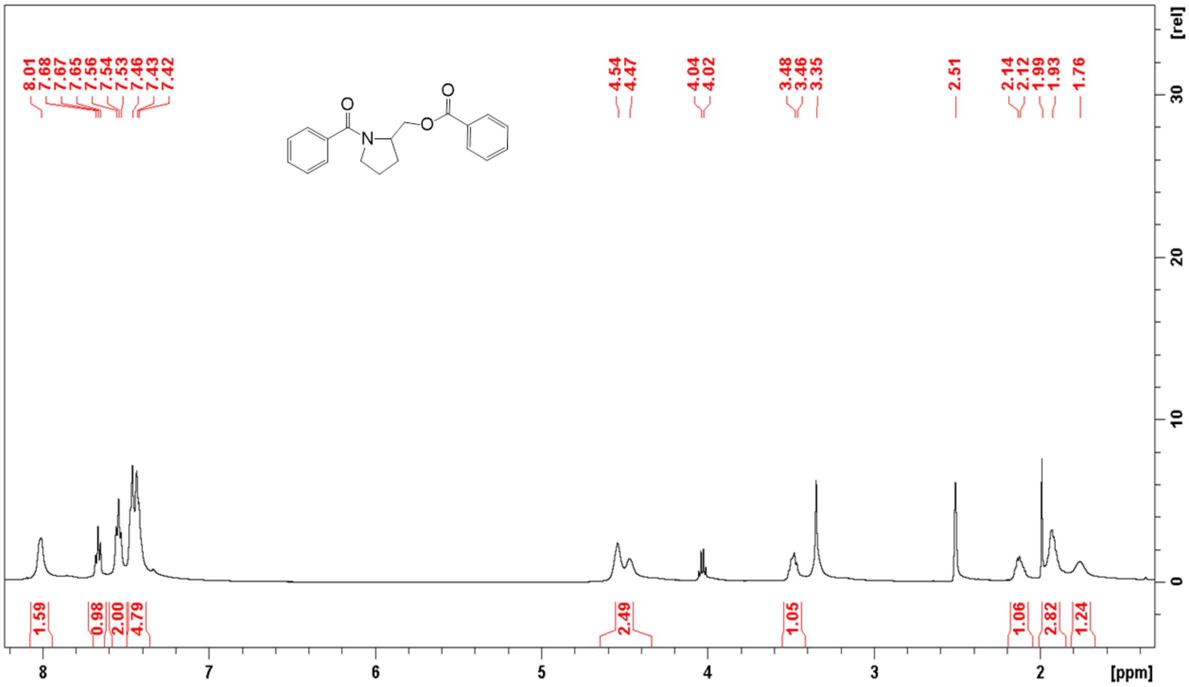

Figure S37. ^{1}H NMR spectrum of **4l** (500 MHz, DMSO-d_6)

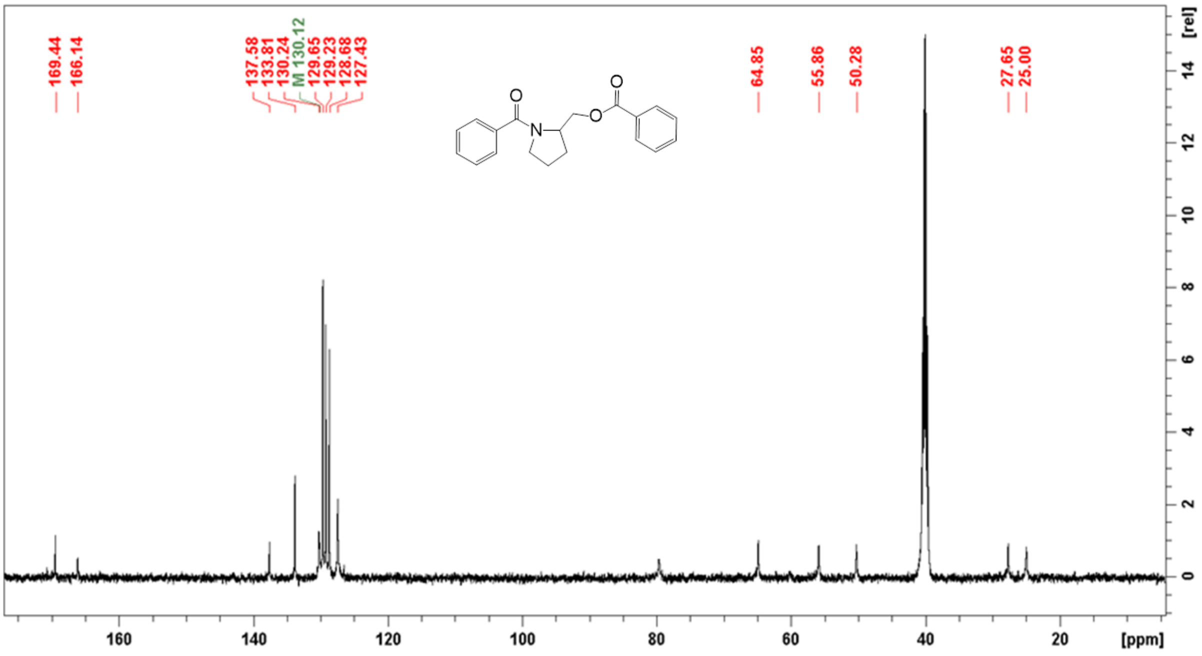

Figure S38. $^{13}C\{^{1}H\}$ NMR spectrum of **4l** (125 MHz, DMSO-d_6)

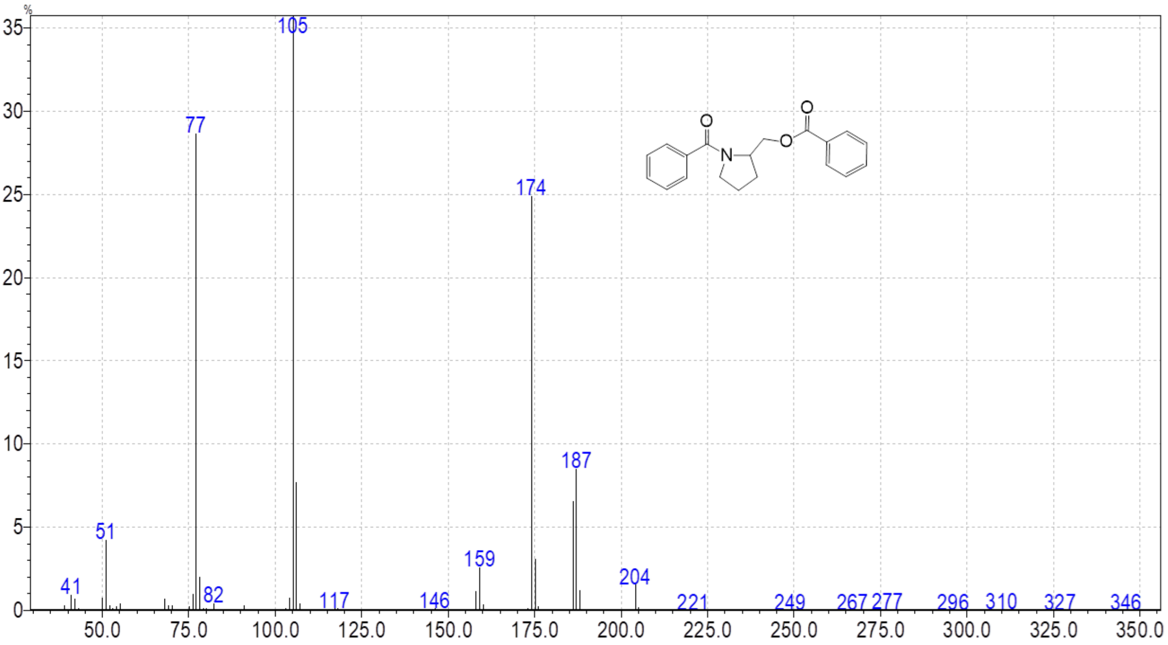

Figure S39. MS spcetrum of **4l** (m/z)

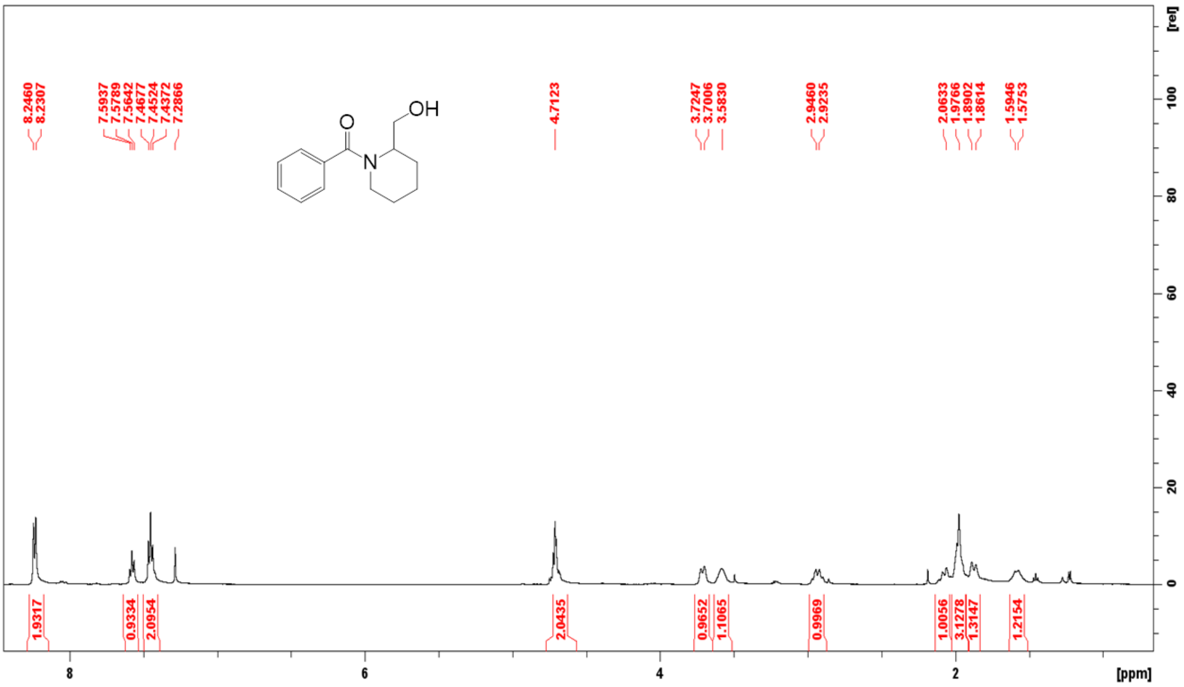

Figure S40. ^{1}H NMR spectrum of **3m** (500 MHz, $CDCl_3$)

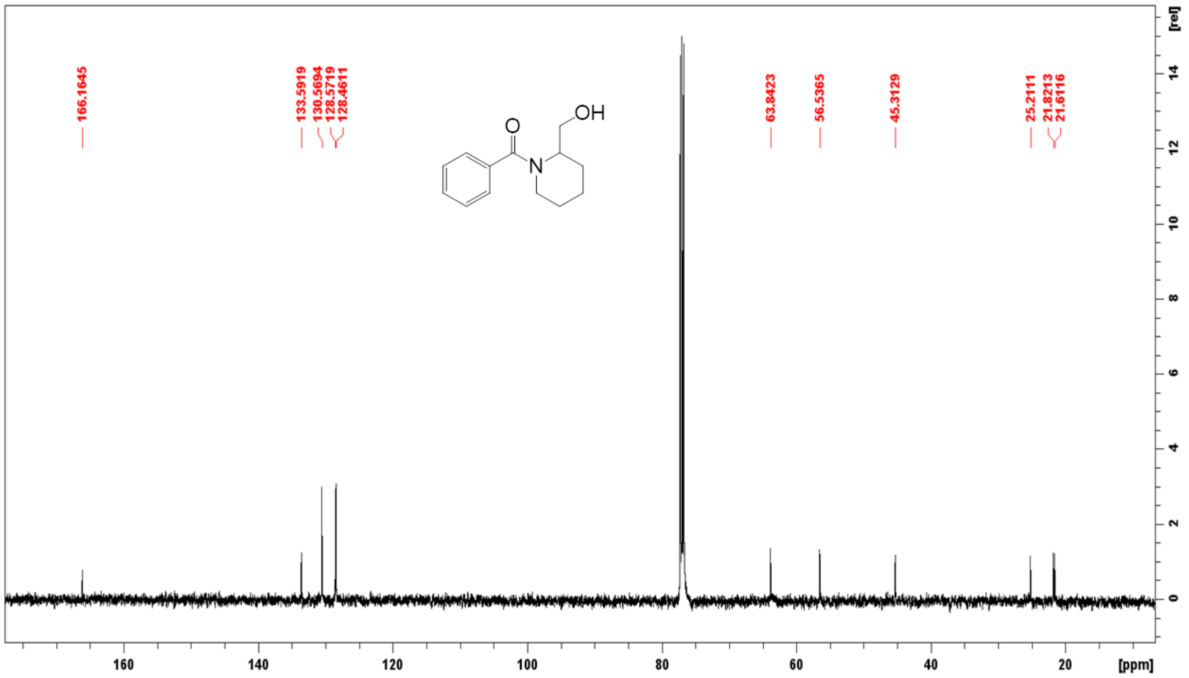

Figure S41. $^{13}C\{^{1}H\}$ NMR spectrum of **3m** (125 MHz, $CDCl_3$)

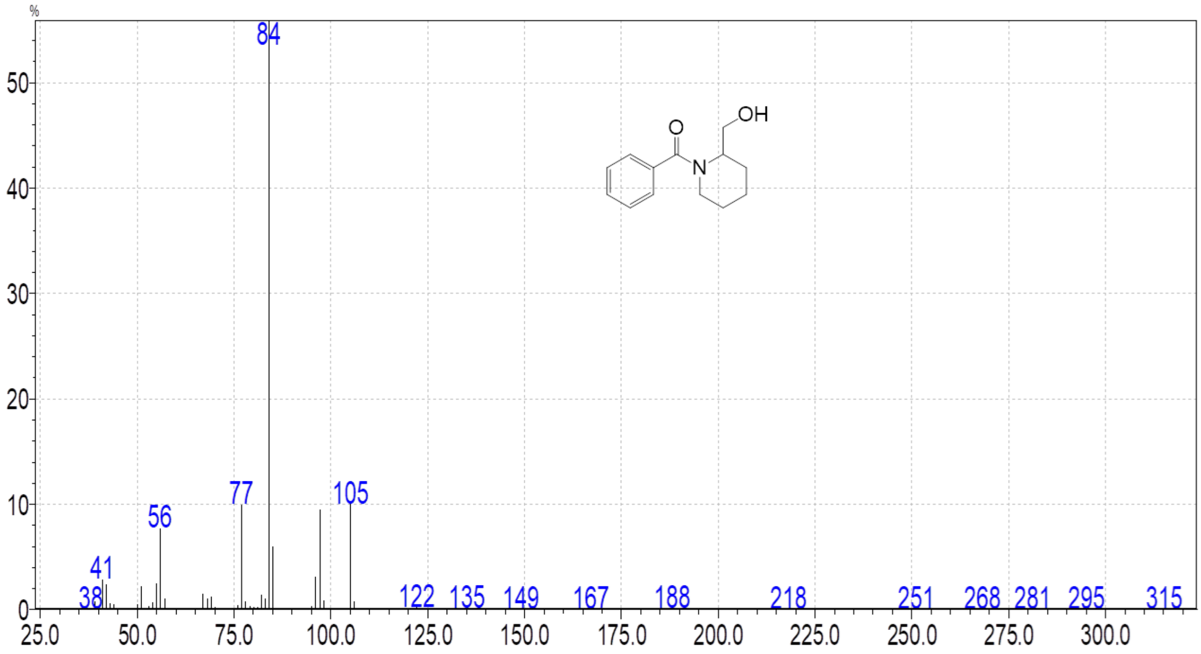

Figure S42. MS spcetrum of **3m** (m/z)

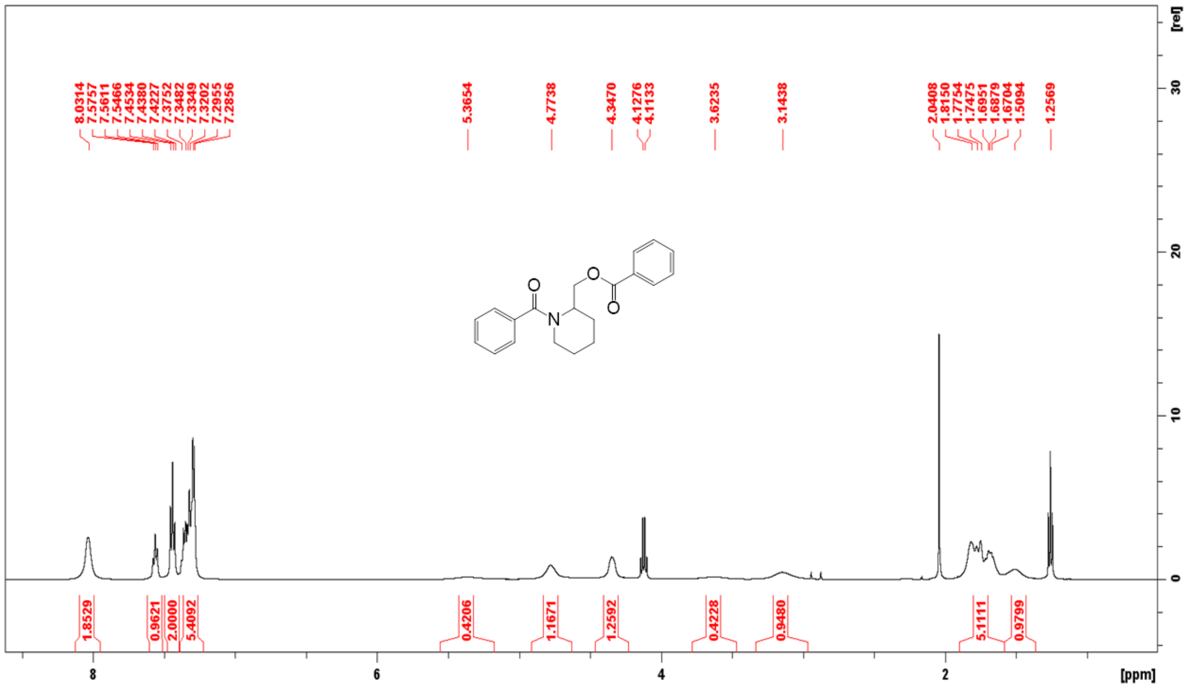

Figure S43. ¹H NMR spectrum of **4m** (500 MHz, $CDCl_3$)

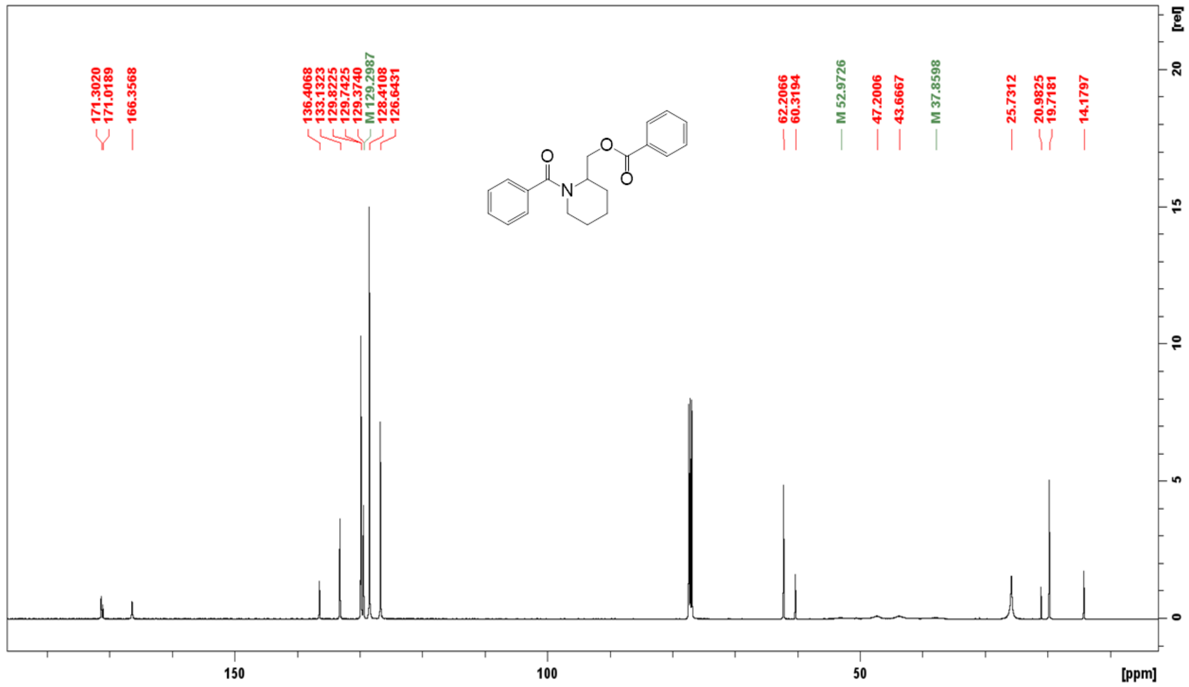

Figure S44. $^{13}C\{^1H\}$ NMR spectrum of **4m** (125 MHz, $CDCl_3$)

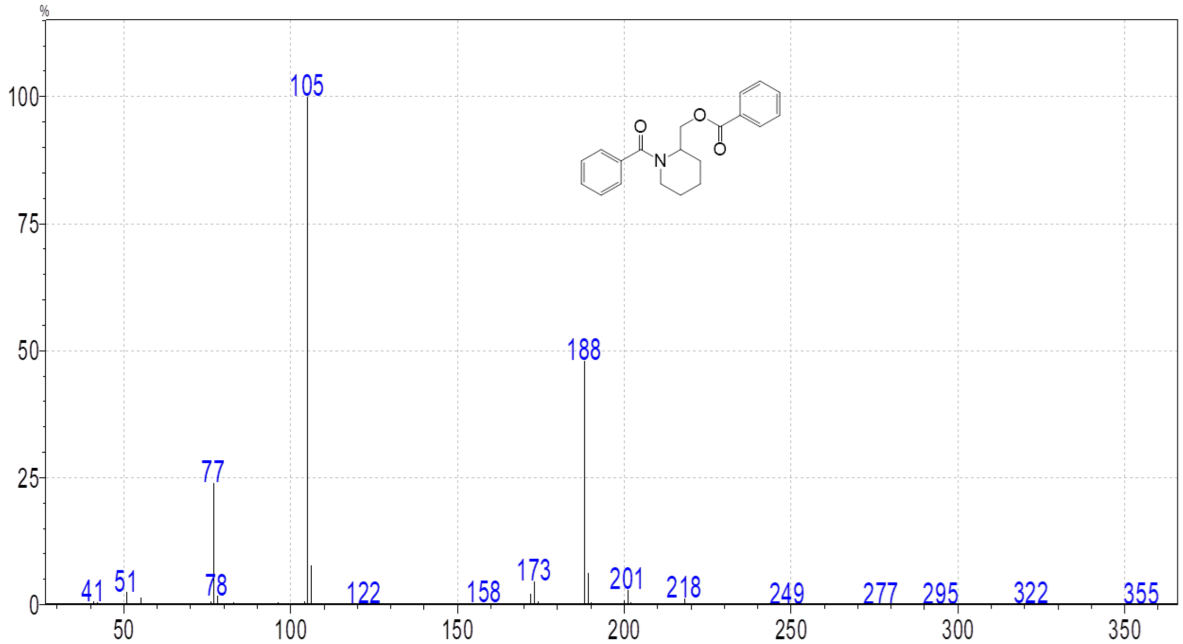

Figure S45. MS spcetrum of **4mn** (m/z)

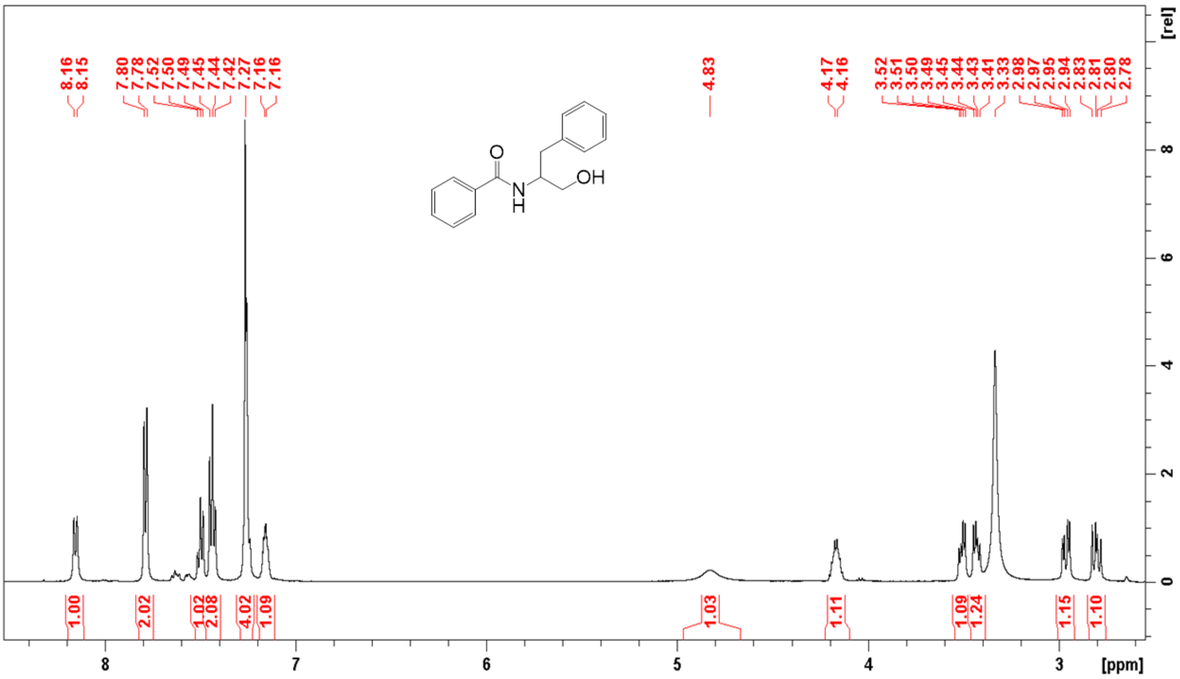

Figure S46. ^{1}H NMR spectrum of **3n** (500 MHz, DMSO-d_6)

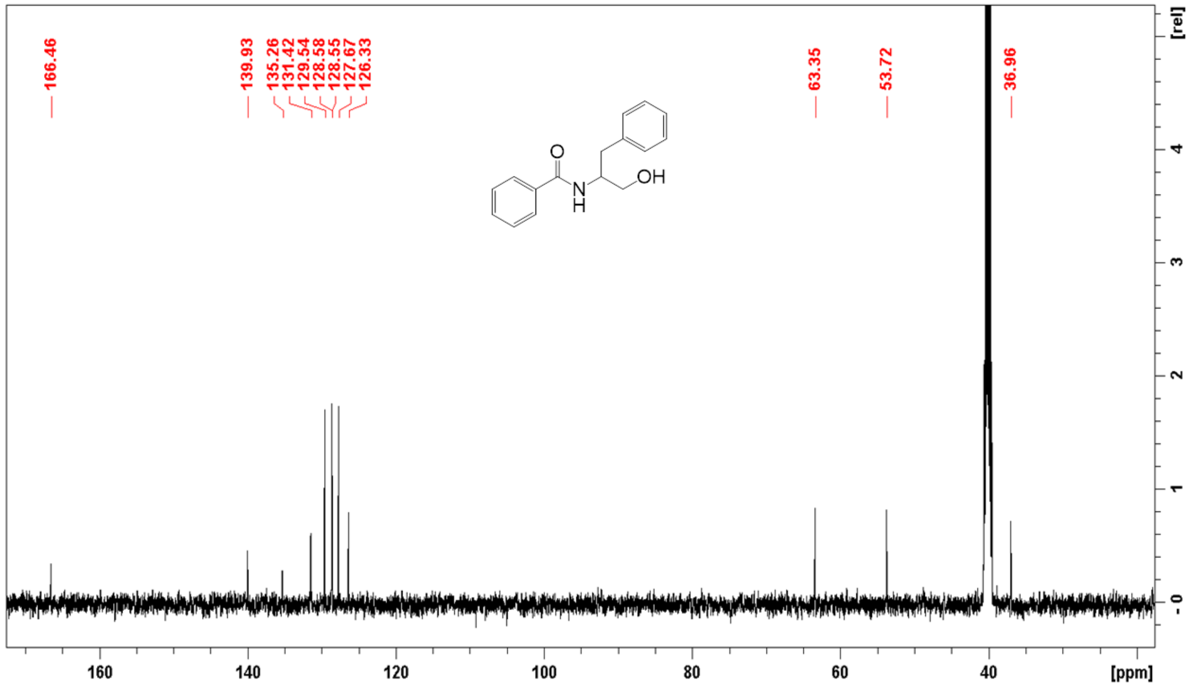

Figure S47. $^{13}C\{^{1}H\}$ NMR spectrum of **3n** (125 MHz, DMSO-d_6)

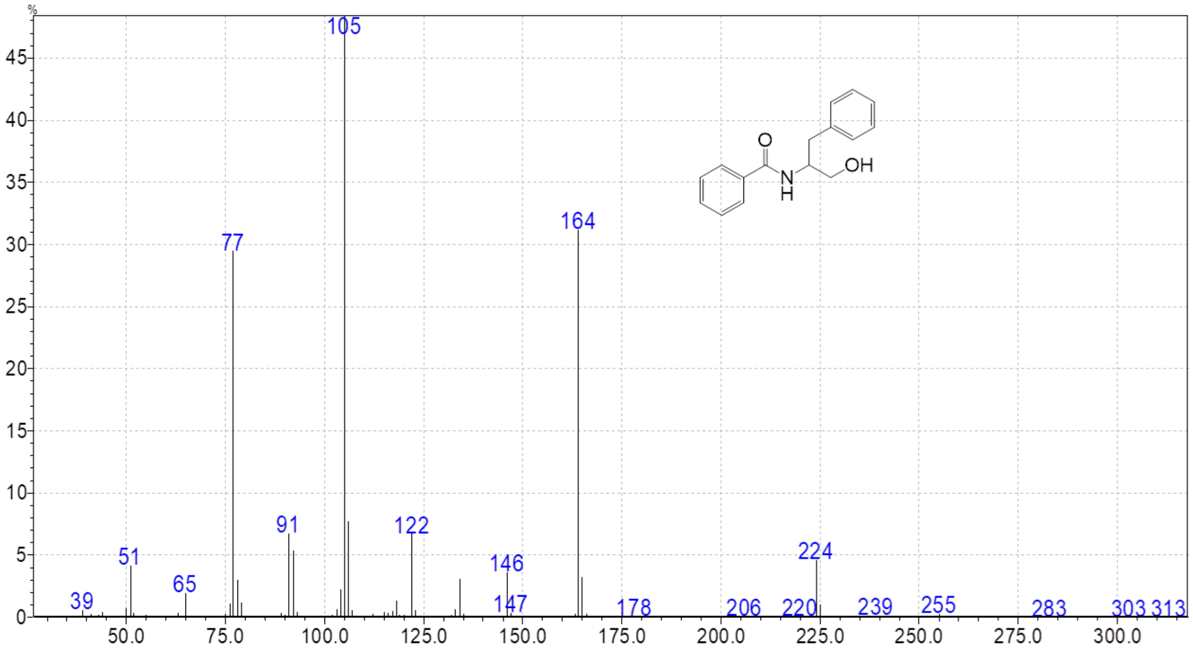

Figure S48. MS spcetrum of **3n** (m/z)

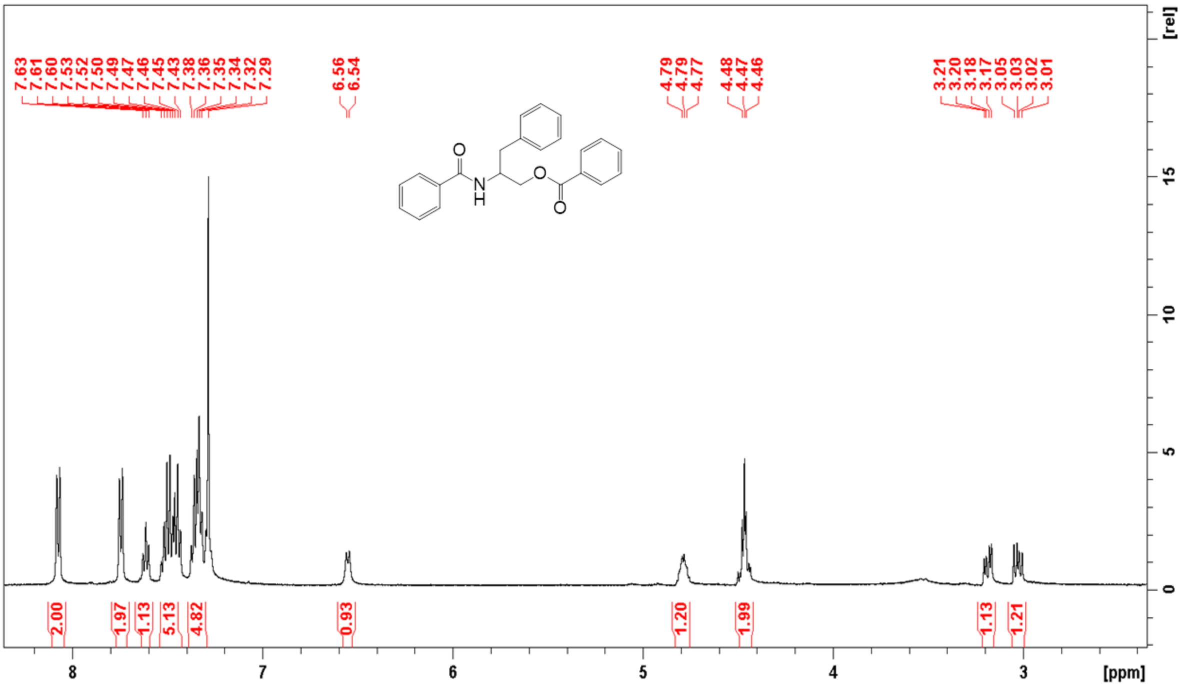

Figure S49. ^{1}H NMR spectrum of **4n** (500 MHz, $CDCl_3$)

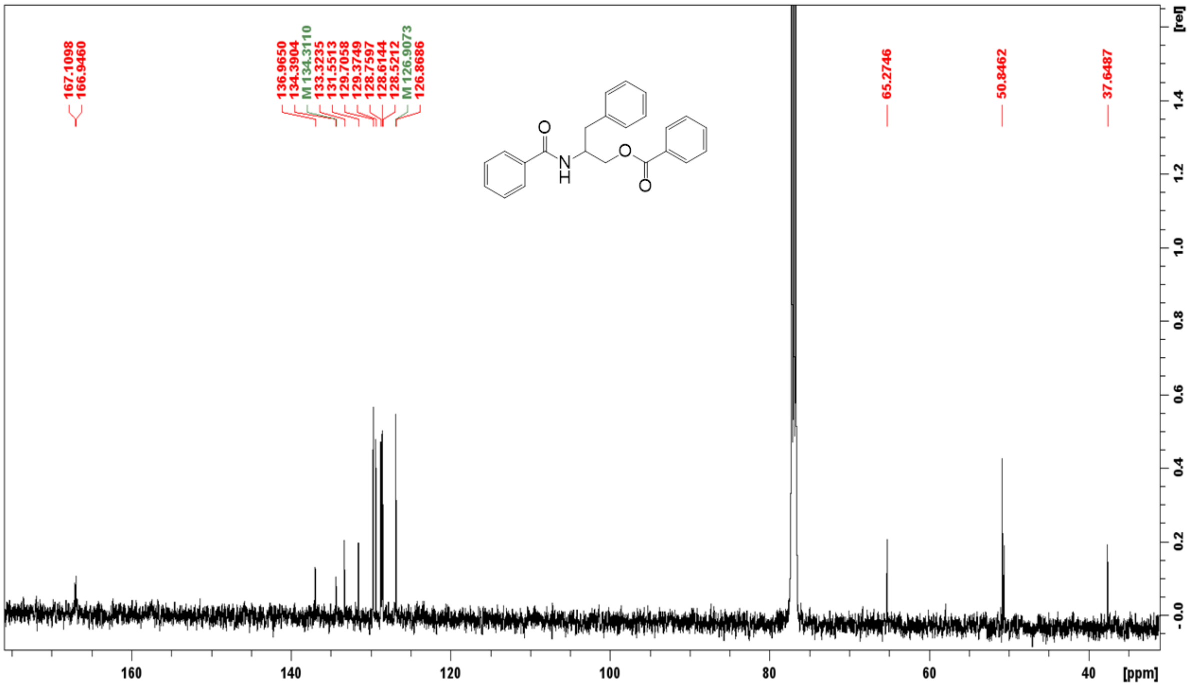

Figure S50. $^{13}C\{^{1}H\}$ NMR spectrum of **4n** (125 MHz, $CDCl_3$)

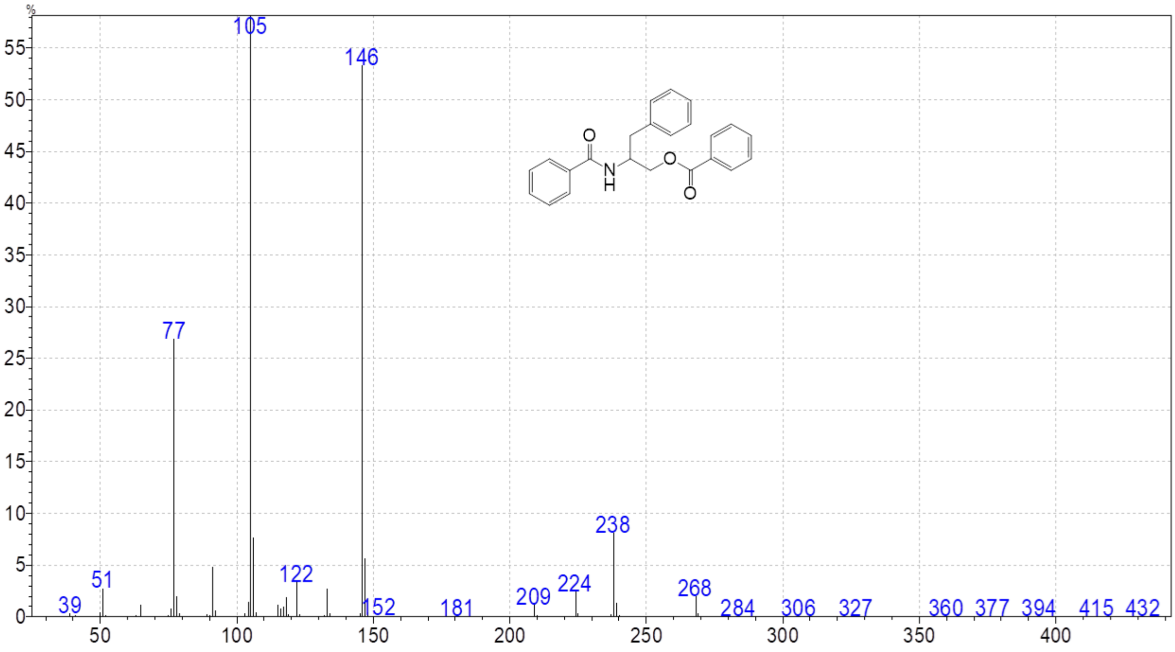

Figure S51. MS spcetrum of **4n** (m/z)

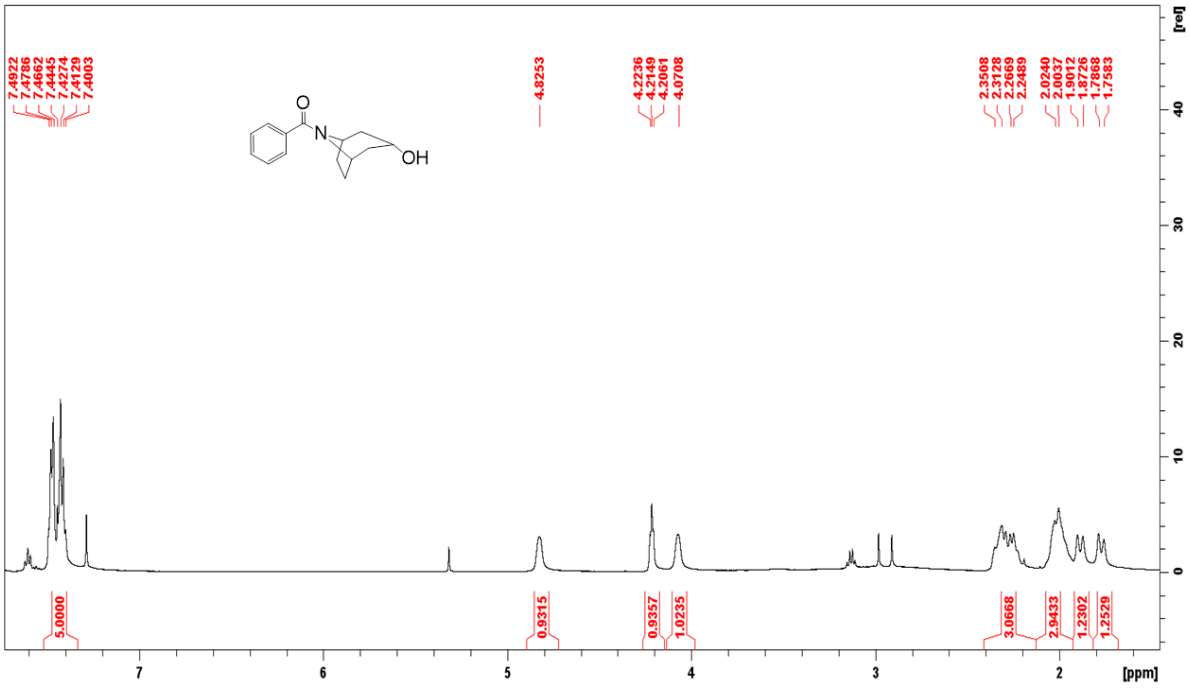

Figure S52. ^{1}H NMR spectrum of **3o** (500 MHz, $CDCl_3$)

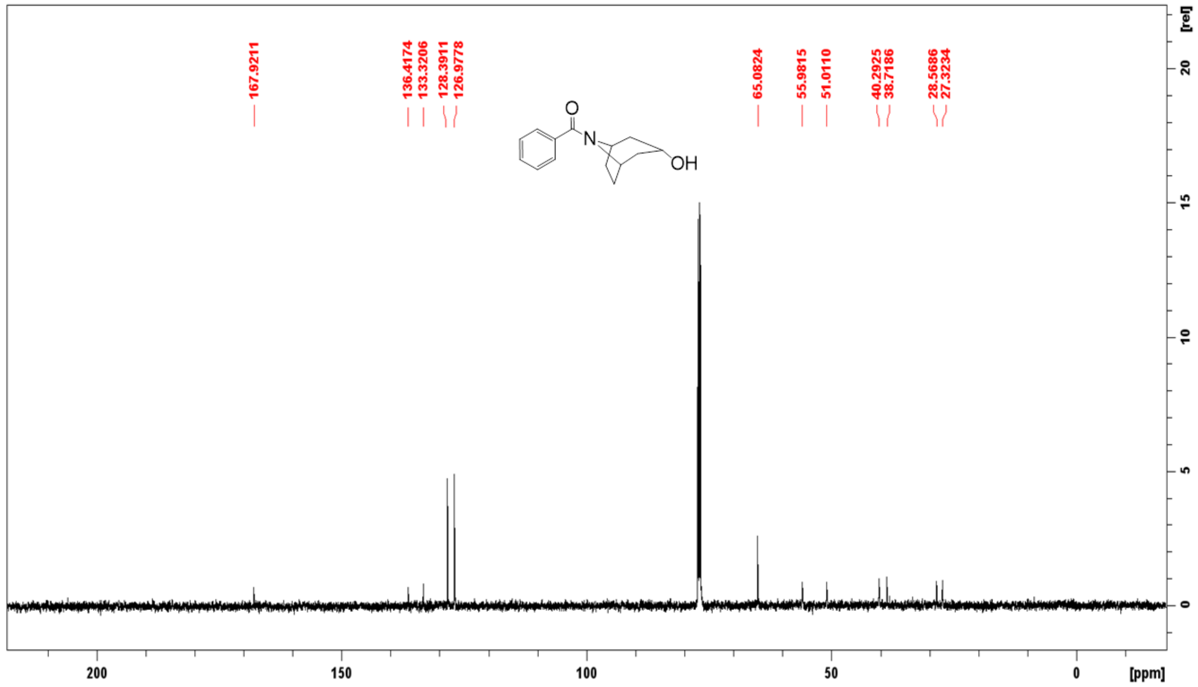

Figure S53. $^{13}C\{^{1}H\}$ NMR spectrum of **3o** (125 MHz, $CDCl_3$)

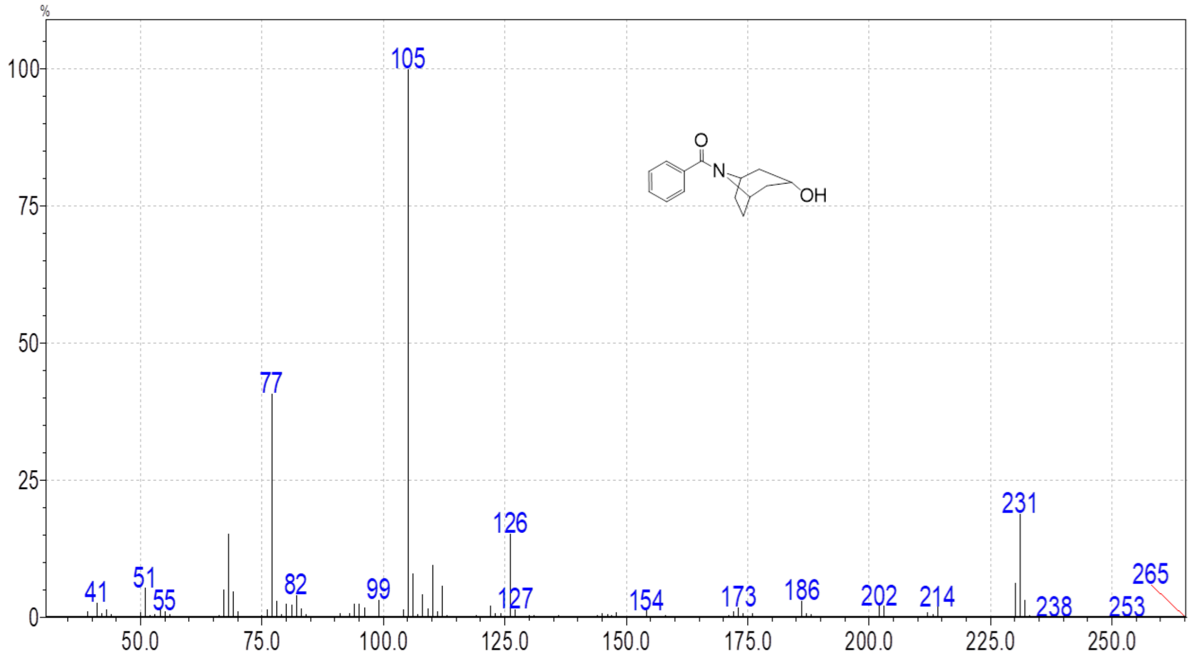

Figure S54. MS spcetrum of **3o** (m/z)

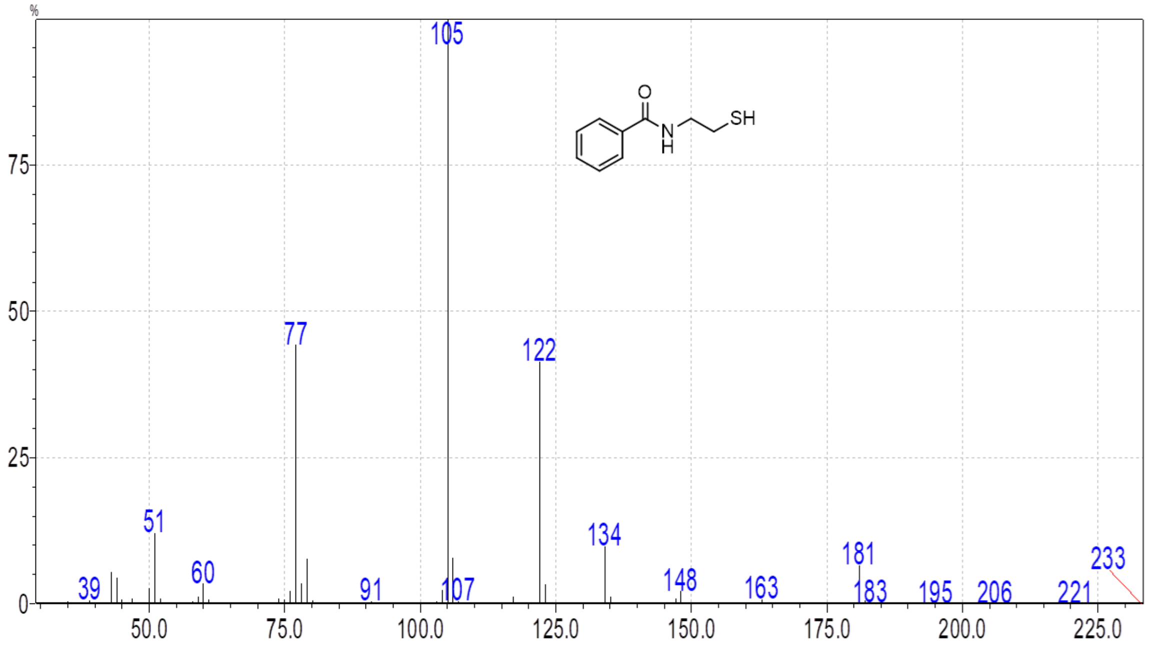

Figure S55. MS spcetrum of **3p** (m/z)

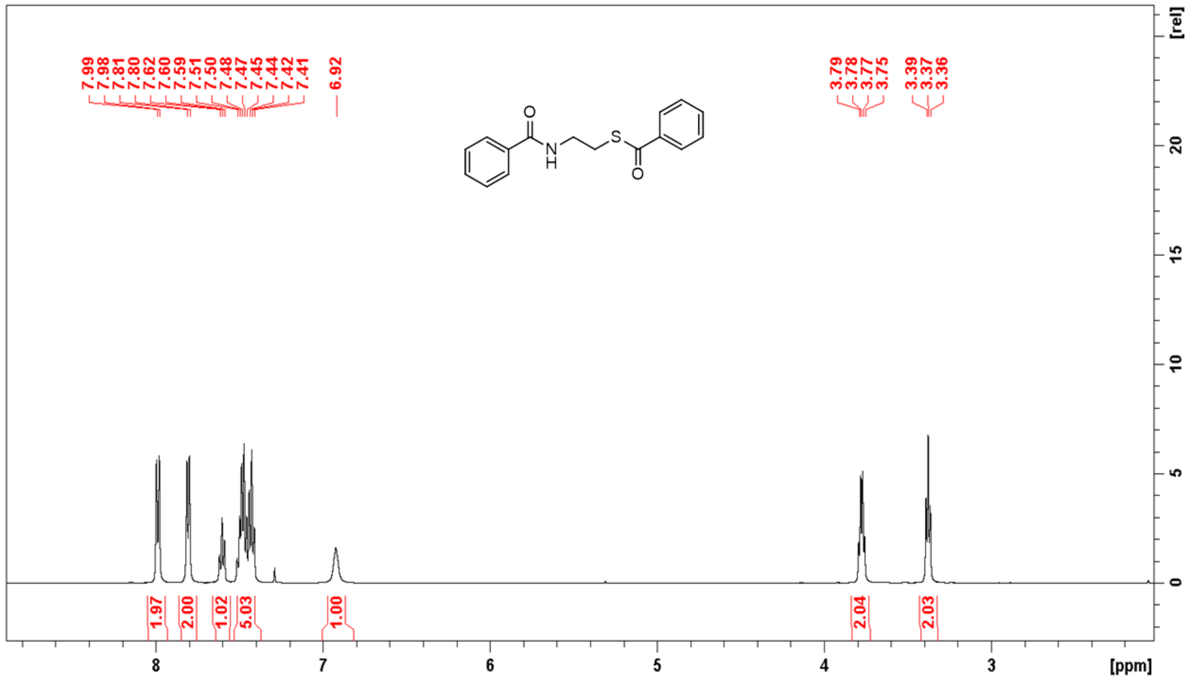

Figure S56. ^{1}H NMR spectrum of **4p** (500 MHz, $CDCl_3$)

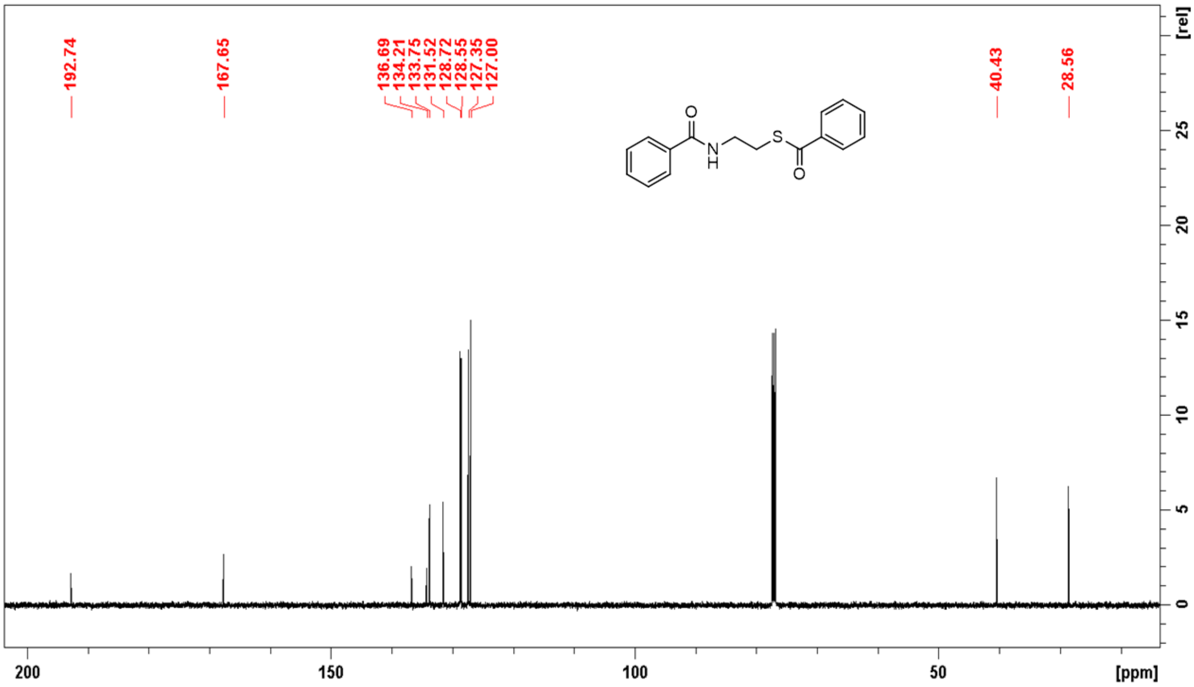

Figure S57. $^{13}C\{^{1}H\}$ NMR spectrum of **4p** (125 MHz, $CDCl_3$)

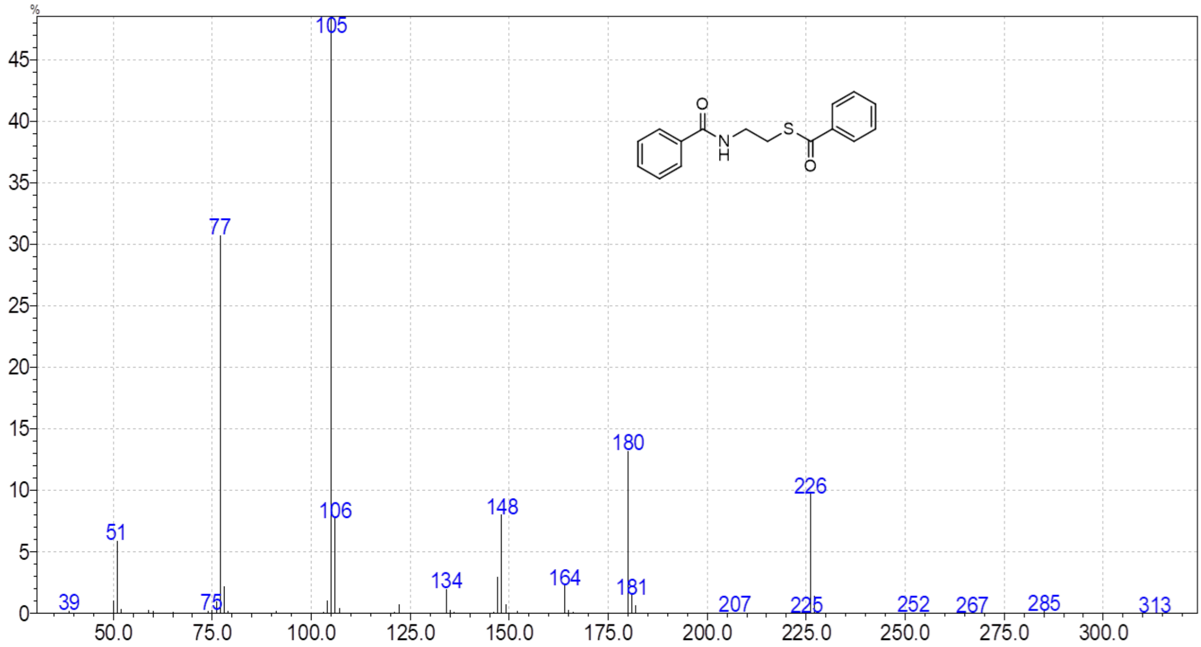

Figure S58. MS spcetrum of **4p** (m/z)

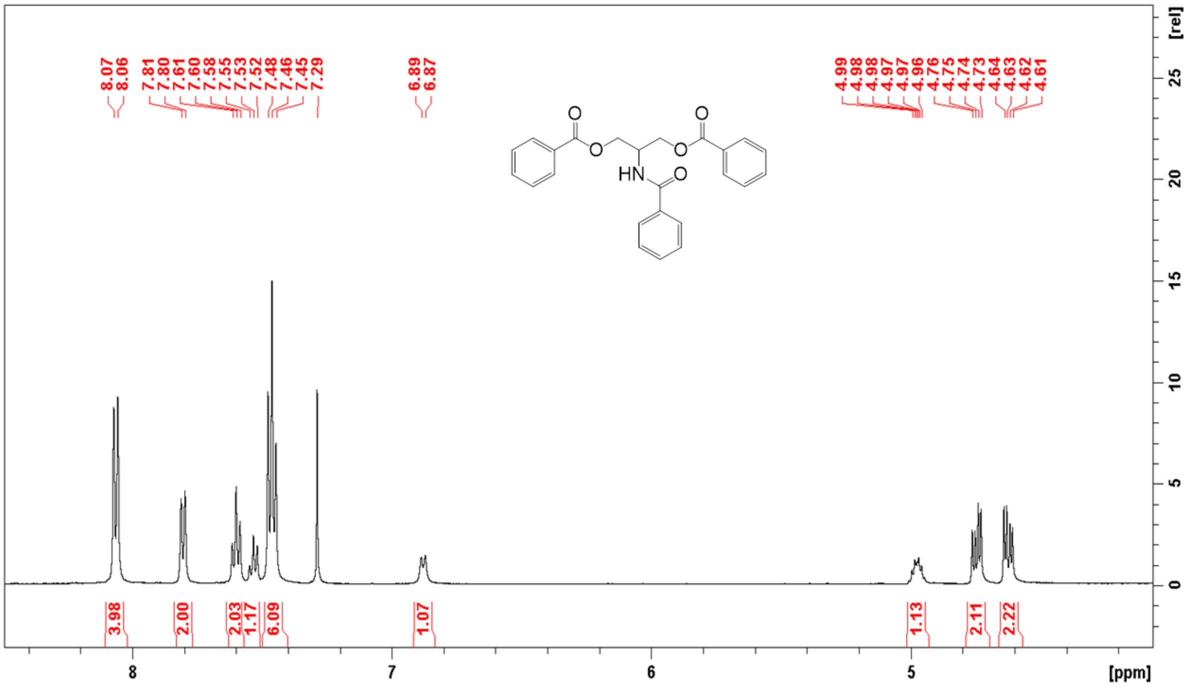

Figure S59. ^{1}H NMR spectrum of **3q** (500 MHz, $CDCl_3$)

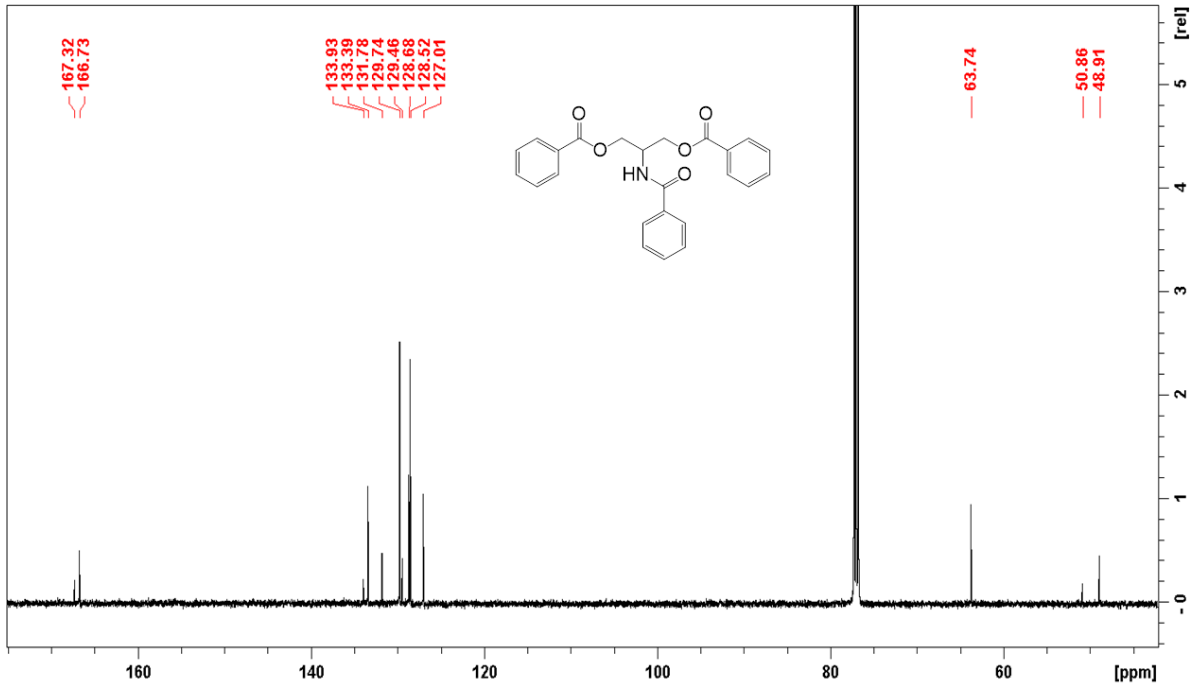

Figure S60. $^{13}C\{^{1}H\}$ NMR spectrum of **3q** (125 MHz, $CDCl_3$)

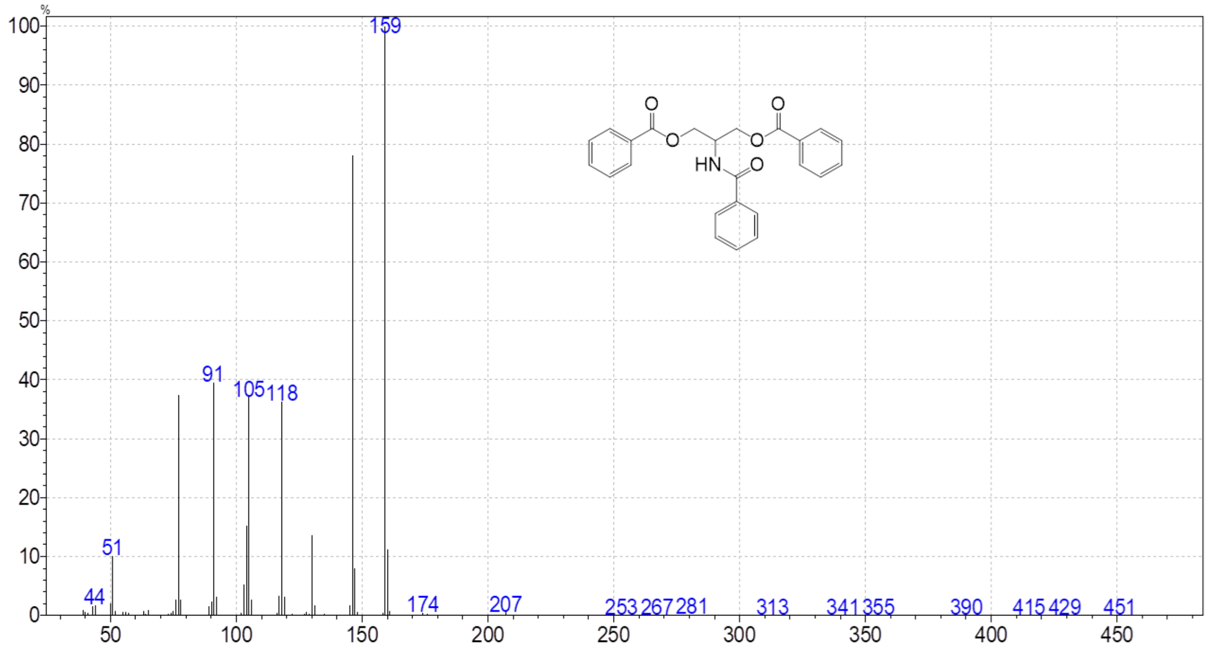

Figure S61. MS spcetrum of **3q** (m/z)

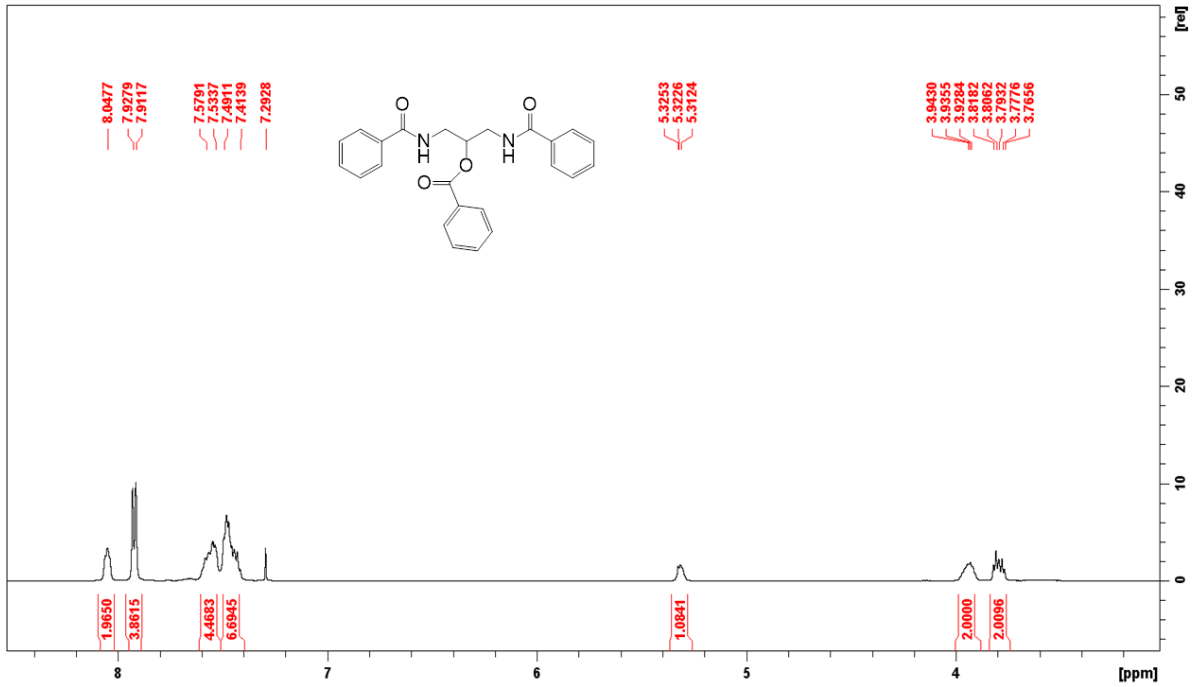

Figure S62. ^{1}H NMR spectrum of **3r** (500 MHz, $CDCl_3$)

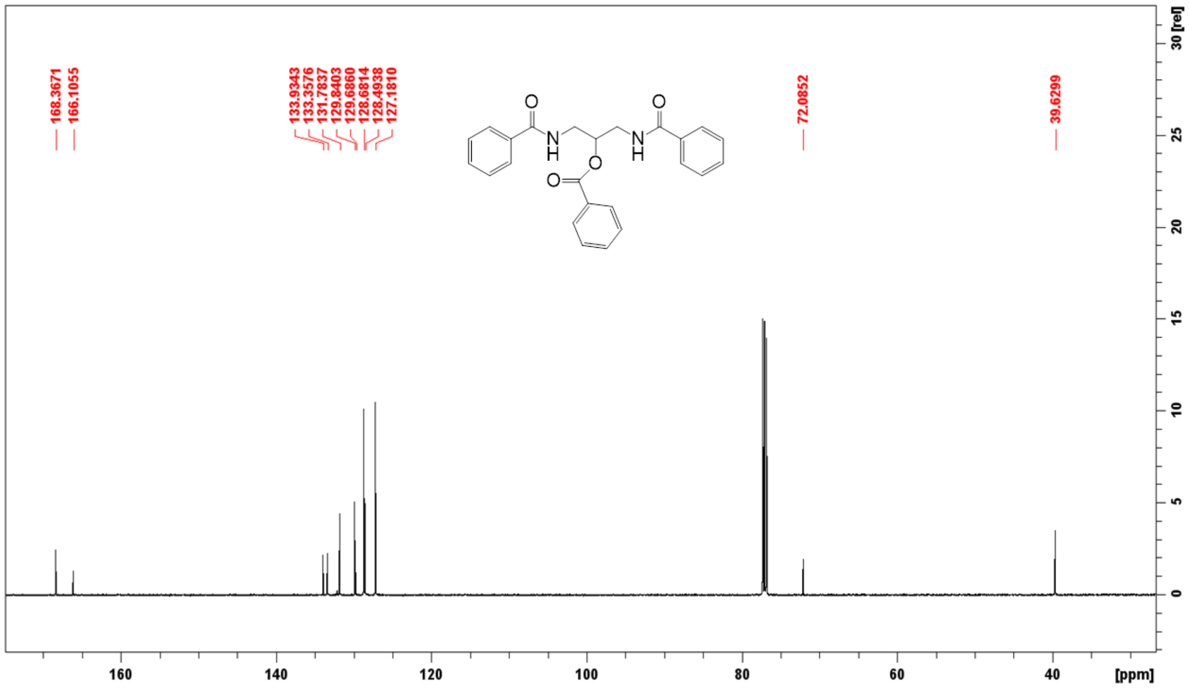

Figure S63. $^{13}C\{^{1}H\}$ NMR spectrum of **3r** (125 MHz, $CDCl_3$)

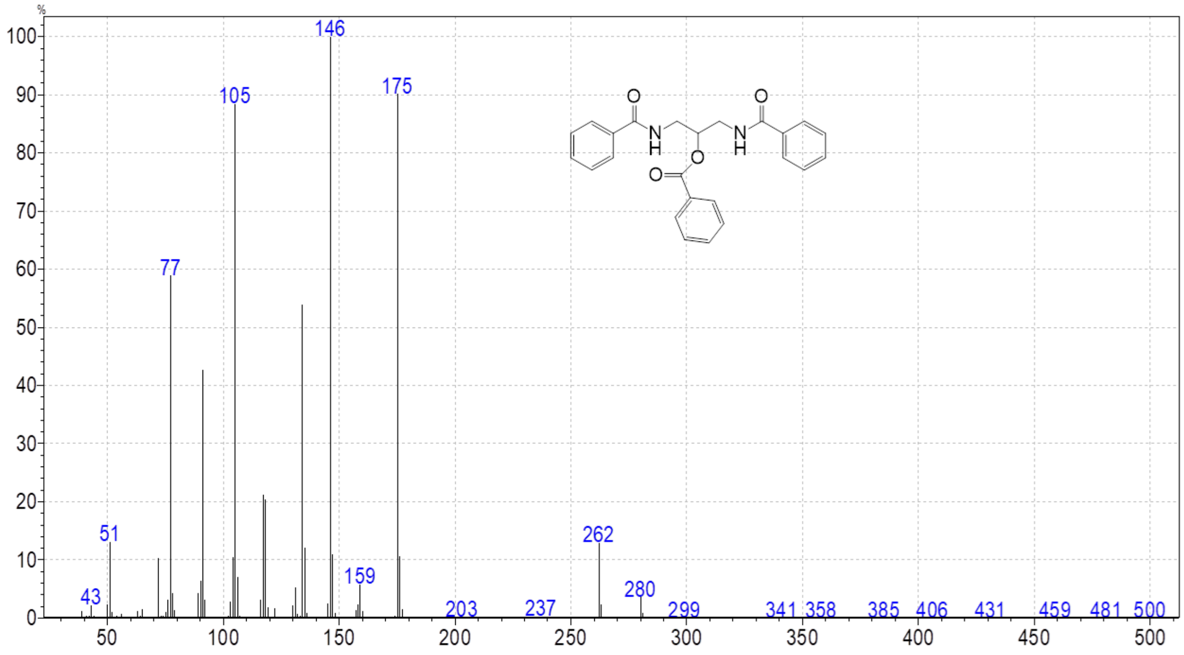

Figure S64. MS spcetrum of **3r** (m/z)
